# Supplementary material for: Mechanistic manifold in a hemoprotein-catalyzed cyclopropanation reaction with diazoketone
Source: Nat Commun. 2023 Dec 2;14:7985. doi: 10.1038/s41467-023-43559-7 (PMC10693563; doi:10.1038/s41467-023-43559-7)
Supplement: Supplementary file 1 — Supplementary Information [file 41467_2023_43559_MOESM1_ESM.pdf]

Supplementary Information

Mechanistic Manifold in a Hemoprotein-Catalyzed Cyclopropanation Reaction with Diazoketone

Donggeon Nam[a,ǂ], John-Paul Bacik[b,ǂ], Rahul L. Khade[c,ǂ], Maria Camila Aguilera[a], Yang Wei[c], Juan D. Villada[a,e], Michael L. Neidig[d,*], Yong Zhang[c,*], Nozomi Ando[b,*], Rudi Fasan[a,e*]

[a] *Department of Chemistry, University of Rochester, Rochester, NY 14627, United States*

[b] *Department of Chemistry and Chemical Biology, Cornell University, Ithaca, NY 14850, United States*

[c] *Department of Chemistry and Chemical Biology, Stevens Institute of Technology, Hoboken, NJ 07030, United States*

[d] *Inorganic Chemistry Laboratory, Department of Chemistry, University of Oxford, South Parks Road, Oxford OX1 3QR, United Kingdom*

[e] *Current affiliation: Department of Chemistry and Biochemistry, University of Texas at Dallas, Richardson, TX 75080, United States.*

ǂ These authors contributed equally to this work.

* Corresponding authors. Email: rudi.fasan@utdallas.edu; nozomi.ando@cornell.edu; yong.zhang@stevens.edu; michael.neidig@chem.ox.ac.uk

Supplementary Figure 1. UV-vis absorption spectra of Mb(H64G,V68A) in the ferric and ferrous form and as free protein and imidazole bound form.

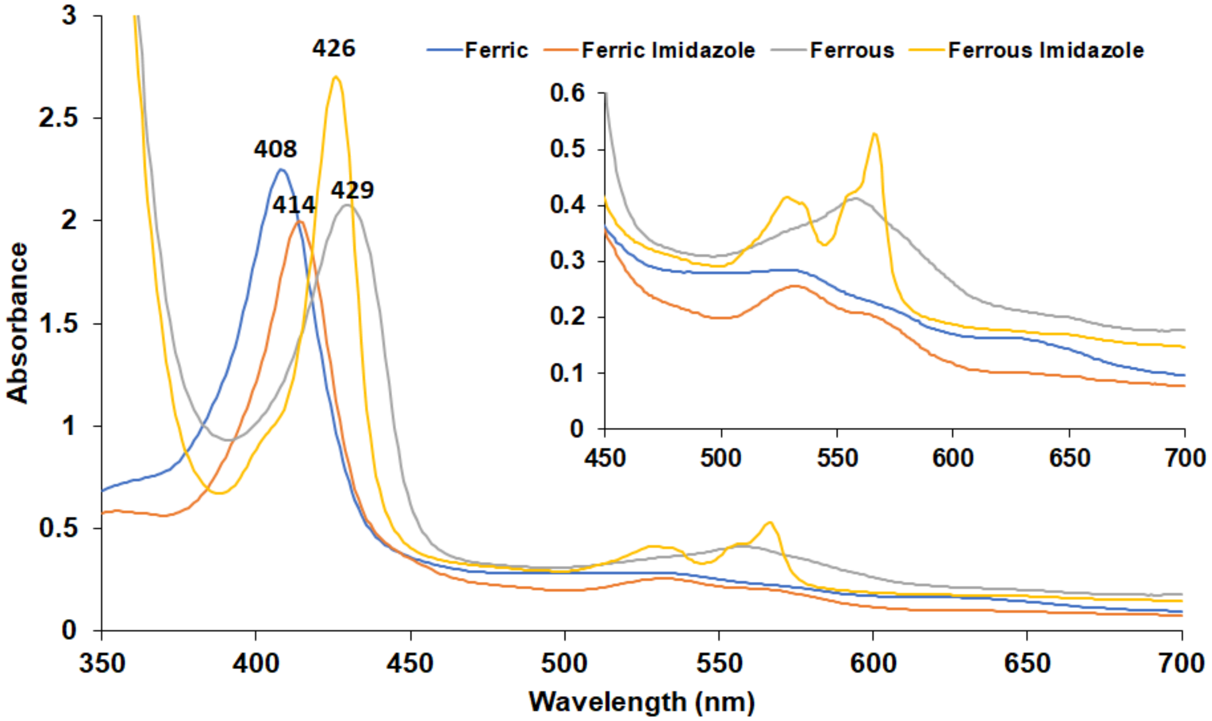

Supplementary Figure 2. Imidazole binding curves for (A) wild-type sperm whale myoglobin (Mb) and (B) Mb(H64G,V68A) in their ferric and ferrous states. Experimental conditions: 10 µM protein in potassium phosphate (50 mM, pH 7), added with 10 mM $Na_2S_2O_4$ (ferrous form only). Binding isotherms were measured based on the change in absorbance at 428 nm (ferrous Mb) or 420 nm (ferric) in difference spectra upon titration of imidazole (0-1 mM for ferrous protein; 0-0.8 mM ferric protein). Error bars represent standard deviation of three separate trials.

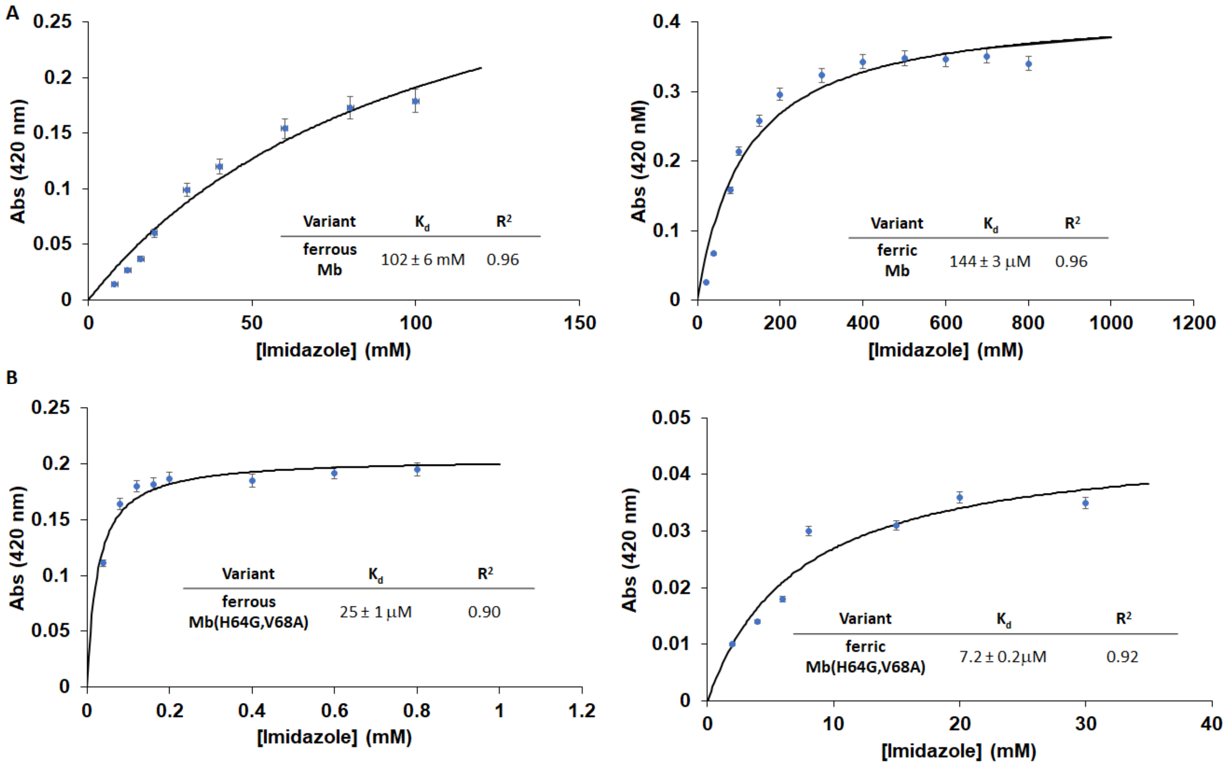

Supplementary Figure 3. Stopped-flow analysis of reduction of Mb(H64G,V68A) with sodium dithionite. (A) UV-vis absorption spectra of the reaction of ferric Mb(H64G,V68A) with $Na_2S_2O_4$ (5 mM) observed every 0.2 s for 30 s (red line: 0.2 s and blue line: 30 s) and (B) a plot of apparent rate constant against the $Na_2S_2O_4$ concentrations. Error bars represent standard deviation of three separate trials.

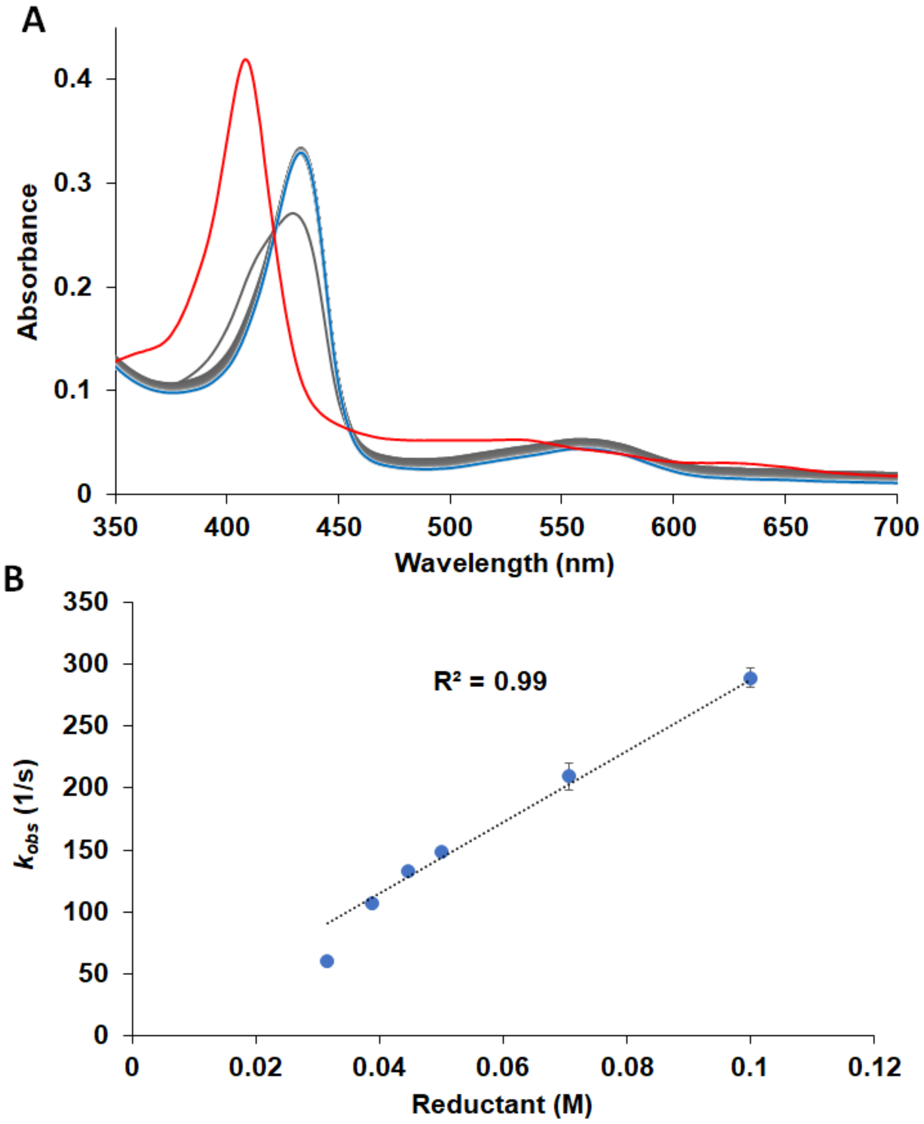

Supplementary Figure 4. Stopped-flow UV-Vis analysis of Mb(H64V,V68A) reaction with BDK in the presence of increasing amounts of styrene (3-20 mM). (**a**) Scheme describing competition between reaction of **Mb-IPC** intermediate with styrene to give ferrous protein and cyclopropanation product vs. conversion of **Mb-IPC** into **Mb-cIII** (N-alkylation). (**b**) Kinetic analysis of a reaction with 20 μM ferrous Mb(H64G,V68A) (pre-incubated with 10 mM $Na_2S_2O_4$), 1 mM BDK, and 3 to 20 mM styrene, as determined via measuring absorbance at 425 nm, every 0.024 s over four minutes. The plot illustrates a variation of the relative ratio of ferrous Mb (= as product of the cyclopropanation reaction) vs **Mb-cIII** depending on the concentrations of styrene. As the styrene concentration is increased, formation of ferrous Mb (and thus cyclopropanation) prevails over **Mb-cIII** formation. Full spectra are shown in **Figure 4c-d**.

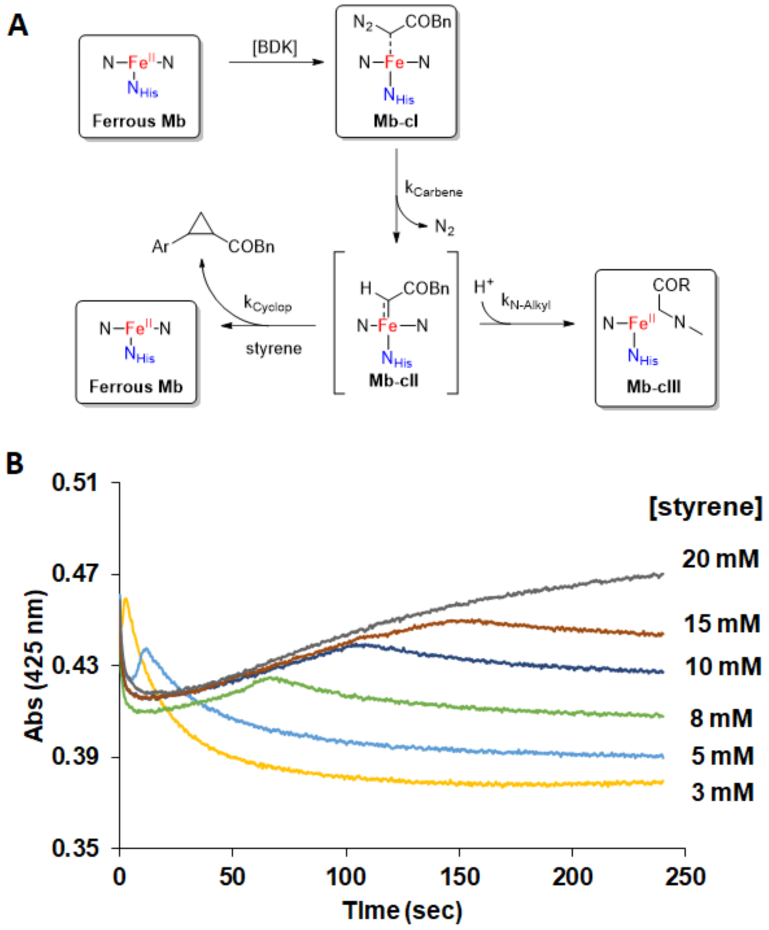

Supplementary Figure 5. Regeneration of Mb(H64G,V68A) from Mb-cIII as monitored via UV-Vis spectroscopy. UV-vis absorption spectra of the reaction of ferrous 3 mM Mb(H64G,V68A) in the presence of 10 mM BDK and 30 mM $Na_2S_2O_4$ over 4 hours. An aliquot of the reaction mixture is diluted in 50 mM KPi buffer before data measurement. These experiments were performed with **Mb-imi** to facilitate detection of the regenerated imidazole-bound ferrous protein based on its characteristic Q bands.

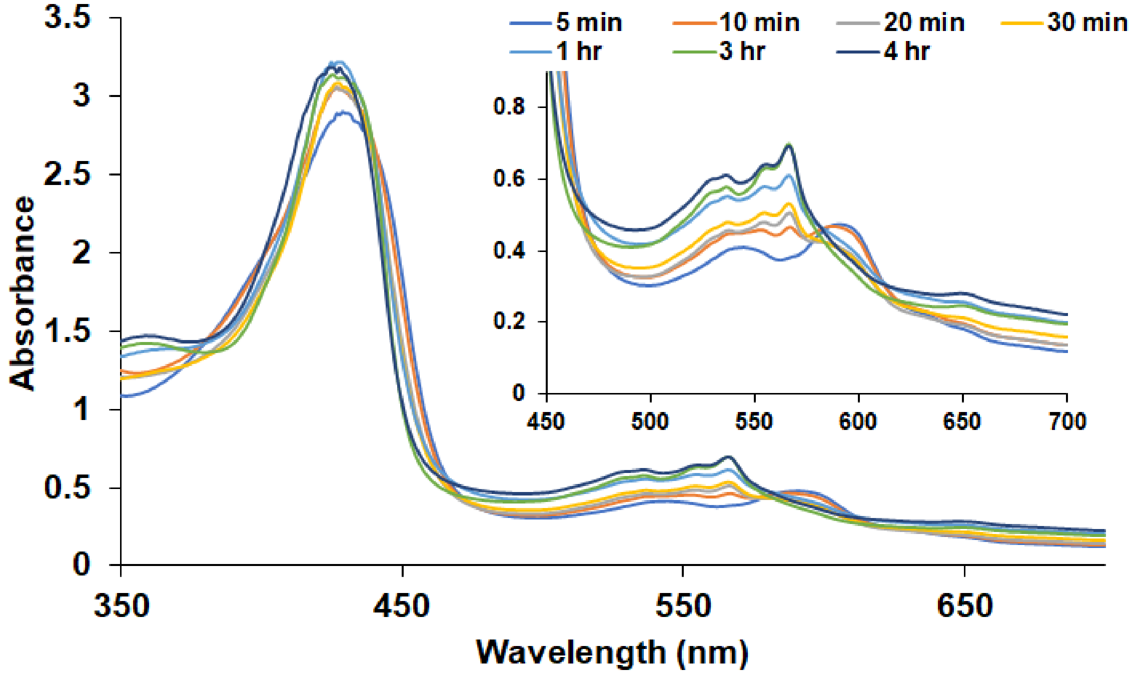

Supplementary Figure 6. A) Time-course analysis of Mb(H64G,V68A)-catalyzed cyclopropanation of styrene with BDK (**2**). Reaction conditions: 10 µM enzyme, 10 mM styrene, 2.5 mM diazoketone, 10 mM $Na_2S_2O_4$ in 50 mM sodium borate buffer (pH 9.0) with 10% EtOH, room temperature, anaerobic atmosphere. Error bars represent standard deviation of three separated trials **B)** Plots of initial rates of product formation for Mb(H64G,V68A)-catalyzed cyclopropanation of styrene with BDK (**2**) at different concentrations of imidazole. Reaction conditions: 10 µM enzyme, 10 mM styrene, 2.5 mM diazoketone 2, 10 mM $Na_2S_2O_4$ in 50 mM sodium borate buffer (pH 9.0) with 10% EtOH, room temperature, anaerobic atmosphere. Reactions were quenched with 50 µL 3M HCl at 0.5 min and 1 min. As reported in Nam *et al.*[1]

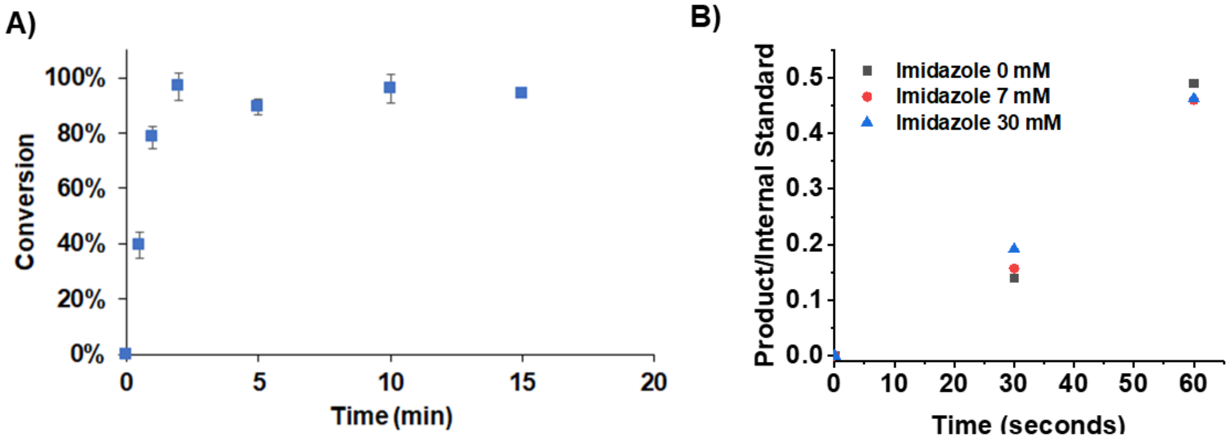

Supplementary Figure 7. Kinetic experiments via Mössbauer spectroscopy. Experimental conditions: 3 mM Fe^{57}-labeled Mb(H64G,V68A) in the presence of 30 mM $Na_2S_2O_4$ and 10 mM BDK. Sample were freeze-quenched at time zero (before BDK addition) and at 0.5 min, 3 min, 5 min, 15 min and 4 hours after addition of BDK. Species A and B corresponds to ferrous **Mb-imi** and **Mb-cIII**, respectively, and their relative amount is indicated. These experiments show nearly quantitative conversion of the ferrous Mb into **Mb-cIII** within three minutes after exposure to BDK, followed by regeneration of the ferrous protein over the subsequent 30-60 minutes.

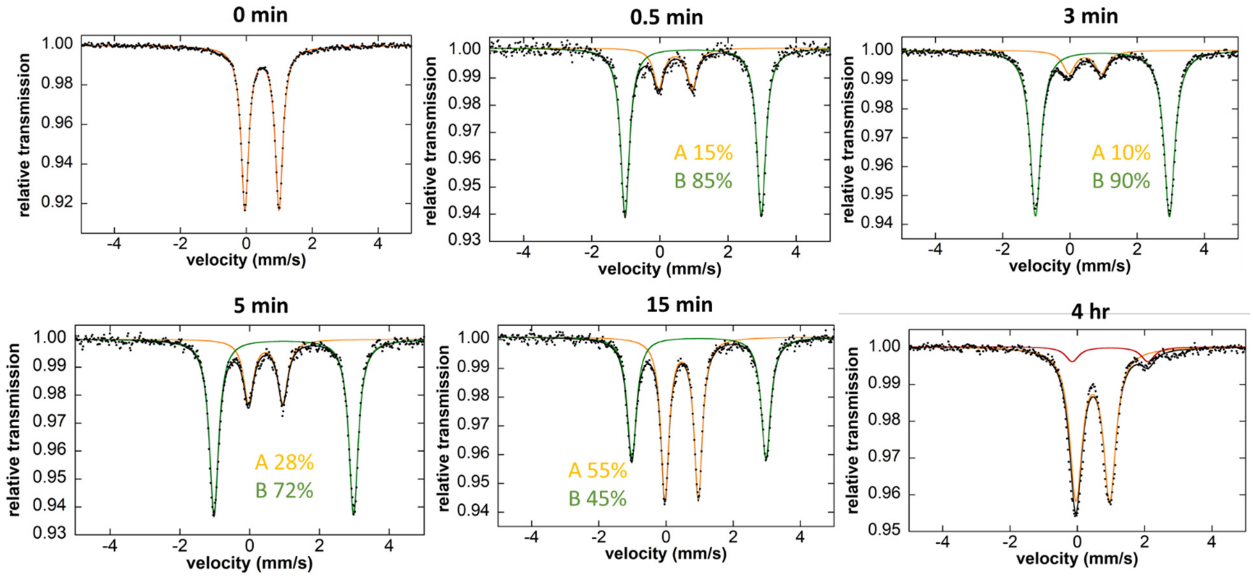

Supplementary Figure 8. Solution- and Mössbauer-based experiments to probe the cyclopropanation activity of **Mb-cIII**. (**a**) Experimental conditions: 3 mM Mb(H64G,V68A), 30 mM $Na_2S_2O_4$, 10 mM BDK, 20 mM styrene added at 0 or 2 minutes, overnight. When adding styrene after pre-incubation of Mb with BDK for two minutes, a ~25% decrease in yield is observed, compared to the reaction with no pre-incubation with BDK. This indicates that about 2.5 mM of BDK was consumed to generate **Mb-cIII** but was not utilized to produce cyclopropane **3**. (**b**) Experimental conditions: 3 mM Mb, 30 mM $Na_2S_2O_4$, 10 mM BDK, 0 or 20 mM styrene added at 15 seconds. 0.5 min total reaction time. Species A and B correspond to ferrous **Mb-imi** and **Mb-cIII**, respectively, and their relative amount is indicated. No effect of styrene on **Mb-cIII** formation was observed. Both experiments agree in indicating that **Mb-cIII** is unreactive toward styrene cyclopropanation.

A

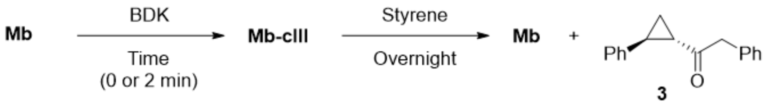

Entry	Time	Yield 3
1	0 minutes	92%
2	2 minutes	76%

B

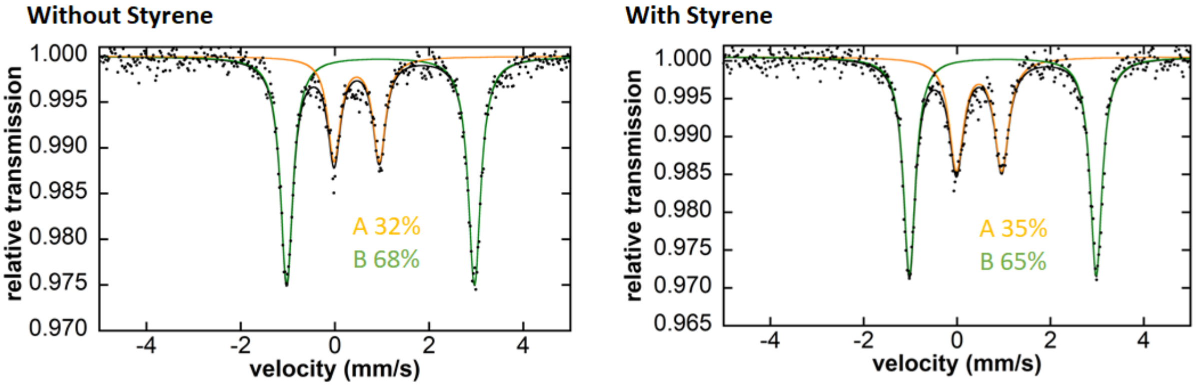

Supplementary Figure 9. Alternative reactions in Mb-catalyzed carbene transfer with BDK. Chemical structures and GC traces of side products generated from reaction of Mb(H64G,V68A) with BDK. Reaction conditions: 3 mM Mb(H64G,V68A), 30 mM $Na_2S_2O_4$, 100 mM BDK, in 50 mM potassium phosphate buffer (pH 7) containing (A) 10% (v/v) ethanol, (B) 10% (v/v) methanol, (C) 10% (v/v) isopropanol, or (D) 10% (v/v) DMSO as co-solvent, 12 hrs, room temperature, anaerobic conditions.

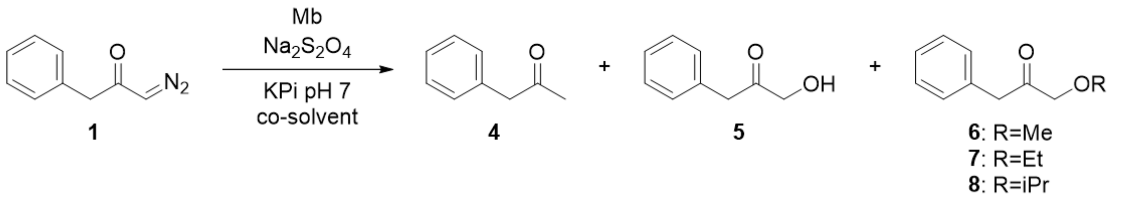

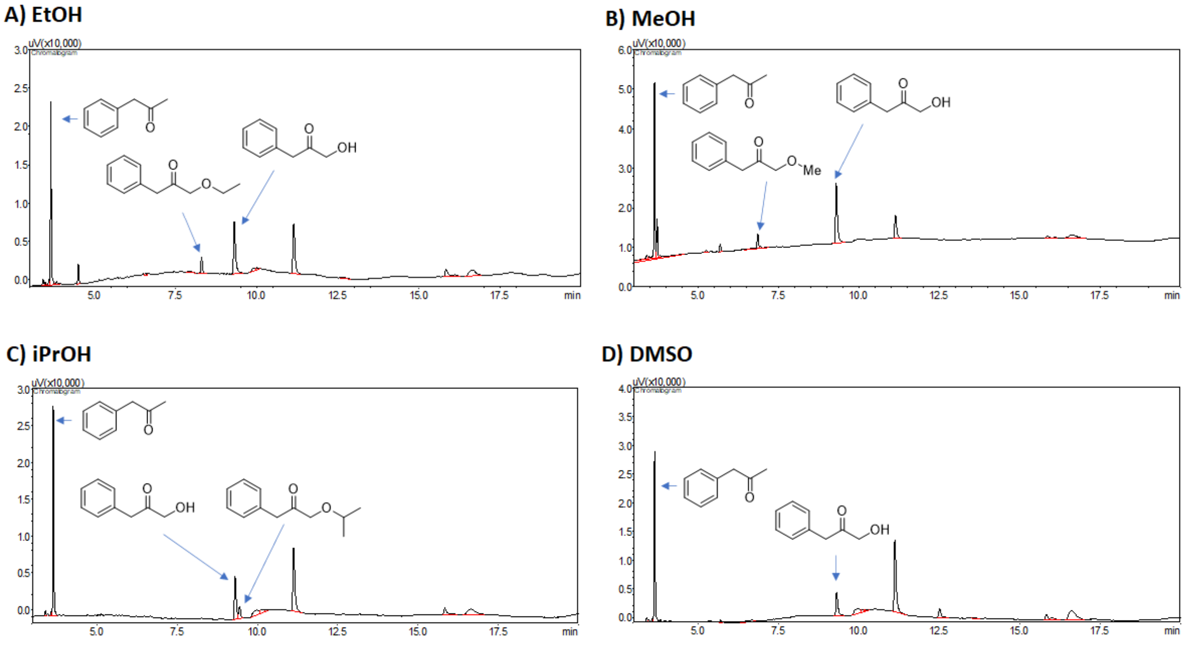

Supplementary Figure 10. Heme extraction experiments. (A) MALDI-TOF MS analysis of heme B and adducts thereof extracted with acetone/HCl from Mb(H64G,V68A) before and after reaction of DPK (0.5 and 3 min). Reaction conditions: 400 μM protein in 50 mM KPi buffer (pH 7), 4 mM $Na_2S_2O_4$, 2 mM BDK. Heme B is detected as $[M]^+$ ion, as reported previously.[2] (B) Chemical structures of heme B, demetalated N-alkylated heme B, and bridged heme-carbene complex. Assignment of heme B and heme B adducts in the MS spectra is supported by matching of the observed isotopic distribution of these species with the expected ones based on molecular formula.

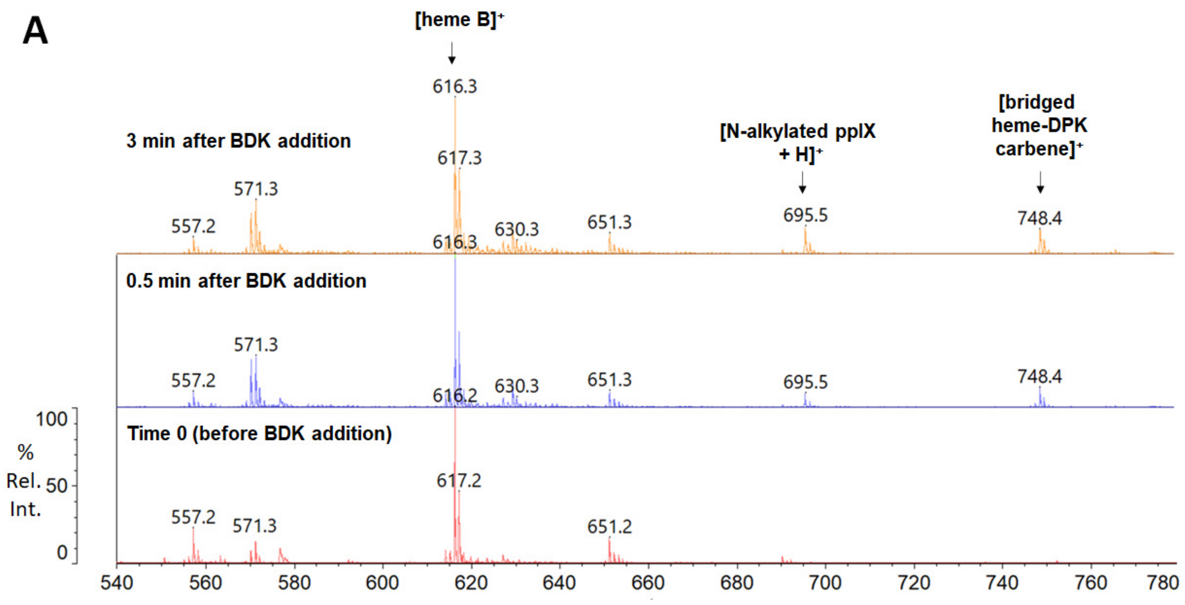

B

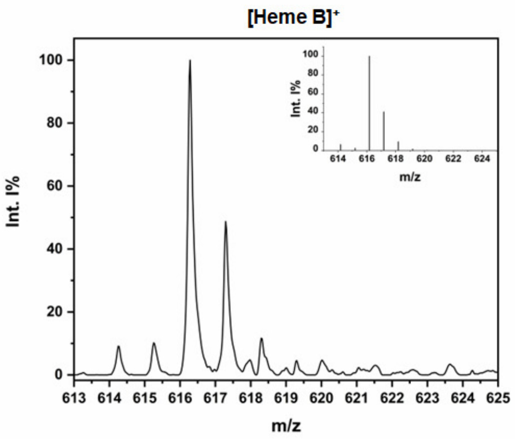

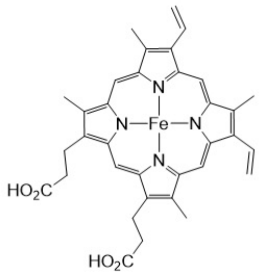

Heme B
MW: 616.50
m/z: 616.18 (100.0%), 617.18 (39.6%), 618.18 (9.2%), 614.18 (6.4%), 615.19 (2.4%), 617.17 (1.5%)

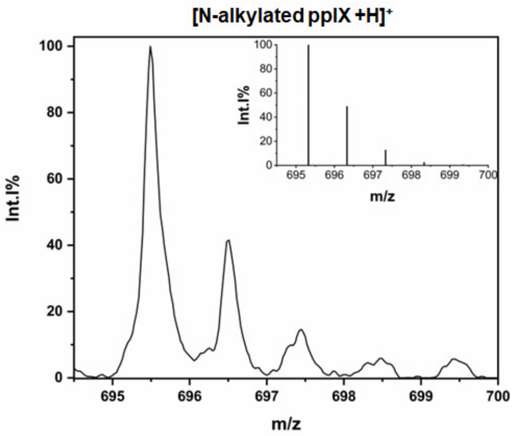

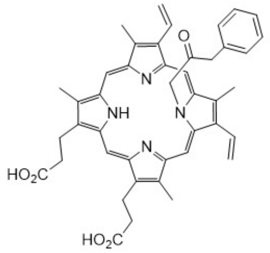

N-alkylated protoporphyrin IX (ppIX)
MW: 694.83
m/z: 694.32 (100.0%), 695.32 (47.2%), 696.32 (12.4%), 697.33 (1.6%), 695.31 (1.5%)

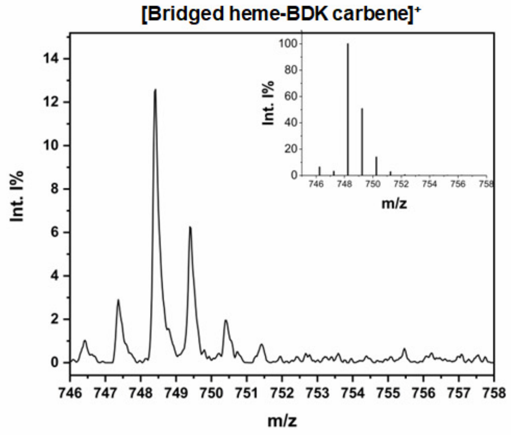

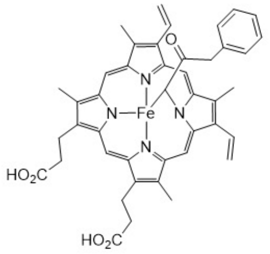

Bridged heme-BDK carbene
MW: 748.66
m/z: 748.23 (100.0%), 749.24 (49.5%), 750.24 (13.7%), 746.24 (6.4%), 747.24 (3.1%), 751.24 (2.7%), 749.23 (1.5%)

Supplementary Figure 11. Optimized conformations for **^{1}Int$_{IPC}$**. **a**) ^{1}Int$_{IPC}$ with benzene ring perpendicular to porphyrin, and **b**) ^{1}Int$_{IPC}$ with benzene ring parallel to porphyrin. Atom color scheme: Fe, black; O, red; C, cyan; N, blue; and H, gray.

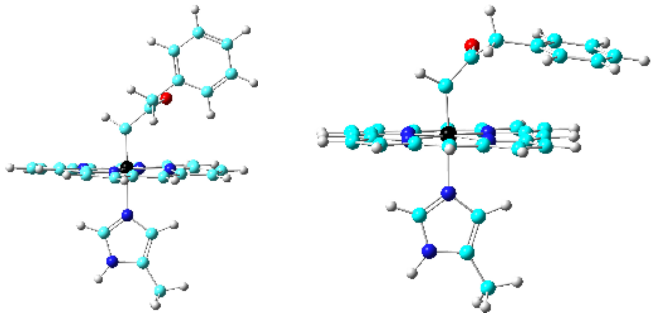

Supplementary Figure 12. Gibbs free energy diagram for heme-catalyzed IPC formation and styrene cyclopropanation with benzyl diazoketone (BDK) with the most favorable spin state for each species. The inserts include diagrams of selected geometric parameters at transition states (in black) and changes from their precursors (in blue), as well as atomic charge changes from precursors to transition states (in black) and charge transfers (in blue) as indicated by arrows. Atom color scheme: Fe, black; O, red; C, cyan; N, blue; and H, gray. In **TS_{CP}**, the small change in the styrene double bond length of 0.016 Å shows early transition state feature. In addition, the different distances between the carbene's carbon and the two carbons in the styrene double bond (2.337 vs. 2.647 Å) indicate a concerted but nonsynchronous character for carbene insertion into the olefin.

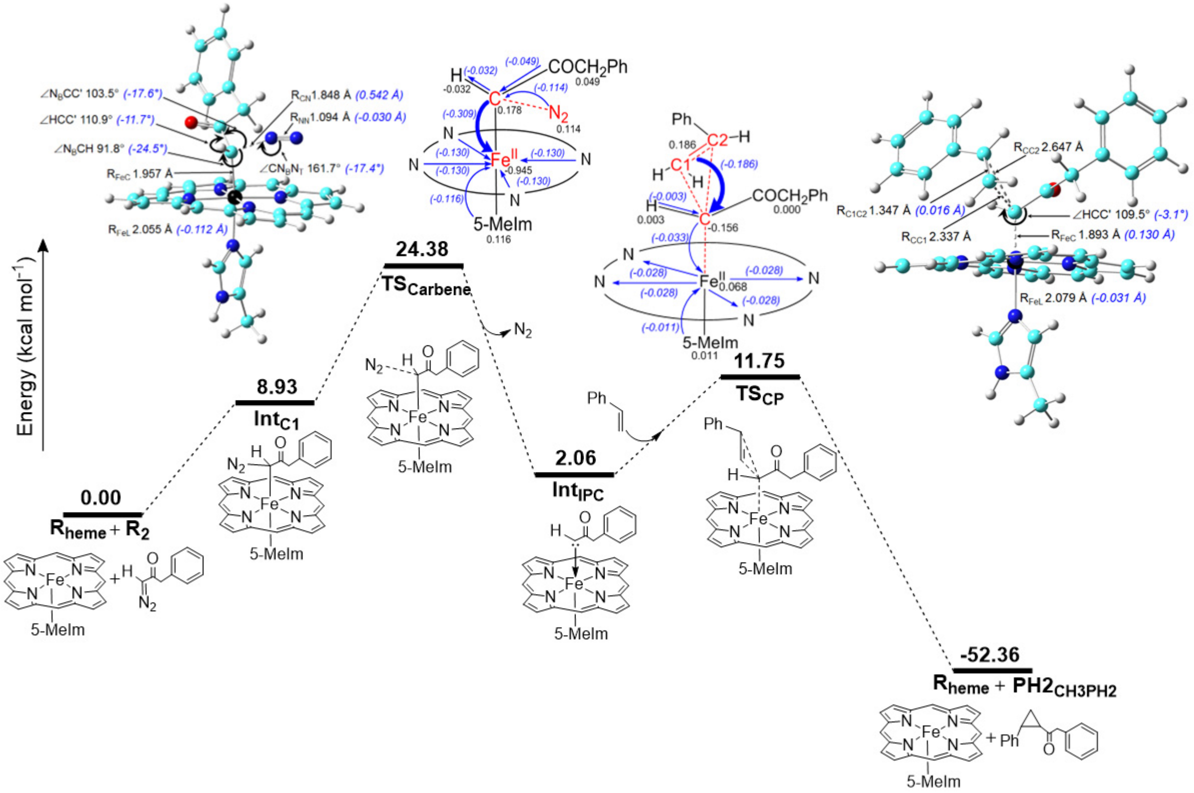

Supplementary Figure 13. Intrinsic reaction coordinate (IRC) results for (**A**) the transition state **TS$_{Carbene}$** for IPC formation from the single C-bound heme-BDK complex, and **B**) the transition state **TS$_{CP}$** corresponding to the styrene cyclopropanation step by IPC.

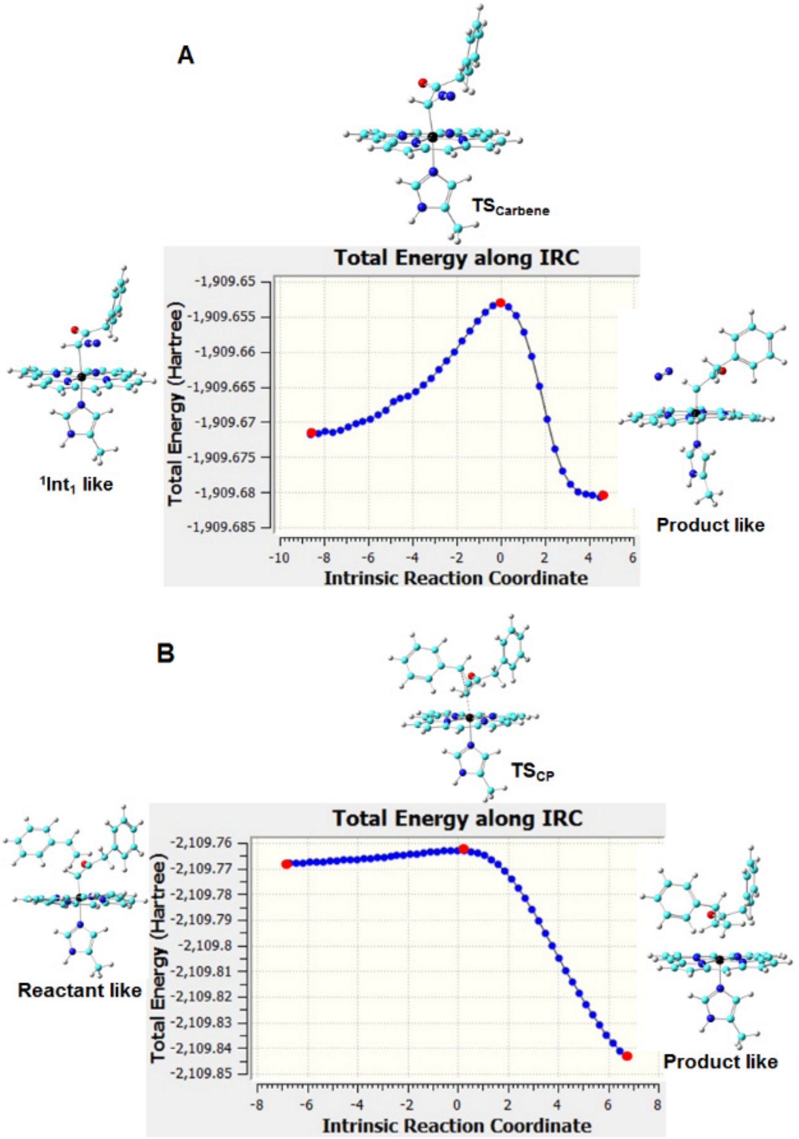

Supplementary Table 1. Mb-catalyzed cyclopropanation of diazoketones in the presence of varying imidazole concentrations. Reaction Conditions: 20 µM Mb(H64G,V68A), 10 mM $Na_2S_2O_4$, 20 mM styrene, 5 mM BDK, 3 hr, 50 mM NaBB pH 9, 10% EtOH.

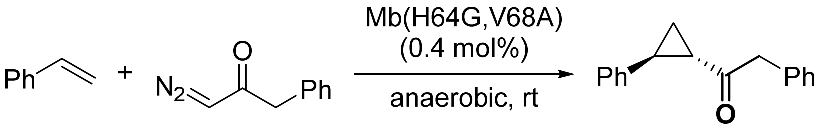

Entry	Imidazole (mM)	Conversion
**1**	0	>99%
**2**	7	97%
**3**	30	>99%
**4**	200	>99%
**5**	1,000	>99%

Supplementary Table 2. Experimental and calculated Mössbauer parameters.[a] Calculated parameters best matching the experimental data are highlighted.

Mössbauer Species	Model	S		δ_{Fe} (mm/s)	ΔE_Q (mm/s)	χ2	Z
**A**			Expt	**0.47**	**1.00**		
	[Fe^{II}(Por)(His)]	2	Calc	0.92	3.13	45.87	0.00
	[Fe^{II}(Por)(His)(Im)]	**0**	**Calc**	**0.61**	**0.84**	**2.14**	**0.12**
		1	Calc	0.80	-1.53	46.67	0.00
		2	Calc	0.95	2.60	37.73	0.00
	[Fe^{II}(Por)(His)(H_2O)]	2	Calc	0.95	2.95	44.64	0.00
		1	Calc	0.78	-1.46	43.43	0.00
		0	Calc	0.67	1.36	4.80	0.01
	[Fe^{III}(Por)(His)(H_2O)]	5/2	Calc	0.43	1.17	0.32	0.72
	[Fe^{II}(Por)(His)(HO^-)]	2	Calc	1.06	3.87	81.28	0.00
		1	Calc	0.60	0.78	1.99	0.14
		0	Calc	0.58	0.48	2.74	0.06
	[Fe^{III}(Por)(His)(HO^-)]	5/2	Calc	0.47	-0.69	15.87	0.00
		3/2	Calc	0.40	1.55	2.18	0.11
		½	Calc	0.29	-2.32	64.54	0.00
**B**			Expt	**0.97**	**4.00**		
	N-alkylated heme complex	**2**	**Calc**	**0.99**	**4.33**	**0.65**	**0.52**
		1	Calc	0.83	-1.80	188.89	0.00
		0	Calc	0.75	1.53	38.83	0.00
	Bridging heme-carbene	2	Calc	0.93	3.75	0.51	0.60
		1	Calc	0.67	-1.53	179.08	0.00
		0	Calc	0.63	1.19	55.66	0.00
	$P_{N\text{-}AlkylatedOH^-}$	2	Calc	1.02	3.37	110.32	0.00
		1	Calc	0.70	2.09	37.35	0.00
		0	Calc	0.67	0.60	22.55	0.00

a) relative energies of spin states of species not reported in other supplementary tables are shown below:

Compound	S	ΔE (kcal/mol)	ΔE_{ZPE} (kcal/mol)	ΔH (kcal/mol)	ΔG (kcal/mol)
[Fe^{II}(Por)(His)(Im)]	0	0.00	0.00	0.00	0.00
	1	12.30	10.67	11.42	7.73
	2	7.98	5.28	6.23	2.05
[Fe^{II}(Por)(His)(H_2O)]	2	0.00	0.00	0.00	0.00
	1	3.39	4.56	4.37	4.67
	0	-3.56	-0.37	-1.59	3.16
[Fe^{II}(Por)(His)(HO^-)]	2	0.00	0.00	0.00	0.00
	1	6.47	7.97	7.57	8.84
	0	-2.68	0.26	-0.75	3.54
[Fe^{III}(Por)(His)(HO^-)]	1/2	0.00	0.00	0.00	0.00
	3/2	16.17	14.80	15.10	13.28
	5/2	8.37	5.44	6.35	2.86
**$P_{N\text{-}AlkylatedOH^-}$**	2	0.00	0.00	0.00	0.00
	1	10.23	11.70	11.02	13.40
	0	6.71	9.08	8.15	12.03

Supplementary Table 3. Cyclopropanation reactions in the presence or absence of free-radical spin trapping reagent. [a] With or without 10 equivalents of DMPO relative to diazoketone. [b] Yields determined by SFC using calibration curves with authentic standards. [c] Reaction conditions: 20 mM styrene, 5 mM BDK (**2**), 10 mM $Na_2S_2O_4$, 20 µM purified protein Mb(H64G,V68A) in sodium borate buffer (50 mM, pH 9) with 10% ethanol, RT, 12 hours. [d] Reaction conditions: 80 mM styrene, 20 mM BDK (**2**), 40 mM $Na_2S_2O_4$, 1 mM Fe(TPP)Cl in toluene with 10% H_2O. [e] Reaction Conditions: 80 mM styrene, 20 mM BDK (**2**), 0.4 mM Co(TPP) in dichloromethane. [f] Diastereomers not detected

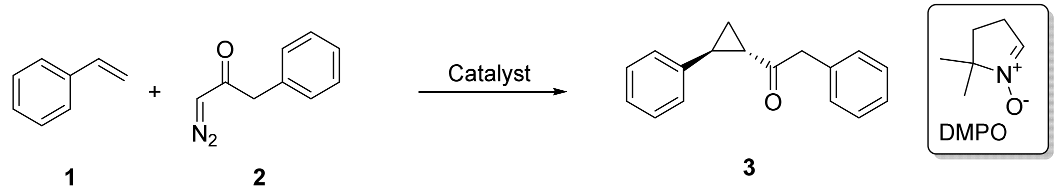

**Entry**	**Catalyst**	**DMPO**[a]	**Yield**[b]	**% *de*trans**	**% *ee***
1	Mb(H64G,V68A)	Yes	56%[c]	99%	99%
2	Mb(H64G,V68A)	No	62%[c]	99%	99%
3	Fe(TPP)Cl	Yes	50%[d]	78%	0%
4	Fe(TPP)Cl	No	36%[d]	77%	0%
5	Co(TPP)	Yes	2%[e]	N/A[f]	N/A
6	Co(TPP)	No	5%[e]	N/A[f]	N/A

Supplementary Table 4. Biocatalytic and chemocatalytic cyclopropanation reactions with *cis*-*β*-deutero-styrene and BDK. [a] Isolated yields. [b] Based on ^{2}H NMR spectrum. [c] Reaction Conditions: 11 mM styrene, 10 mM BDK (**2**), 10 mM $Na_2S_2O_4$, 20 μM purified protein Mb(H64G,V68A) in sodium borate buffer (50 mM, pH 9) with 10% ethanol, RT, 12 hours. [d] Reaction Conditions: 11 mM styrene, 10 mM BDK (**2**), 20 mM $Na_2S_2O_4$, 0.5 mM Fe(TPP)Cl in toluene with 10% water. [e] Reaction Conditions: 11 mM styrene, 10 mM BDK (**2**), 0.2 mM Co(TPP) in DCM. [f] Reaction conditions: 11 mM styrene, 10 mM BDK (**2**), 10 mM $Na_2S_2O_4$, 60 μM Hemin in sodium borate buffer (50 mM, pH 9) with 10% ethanol.

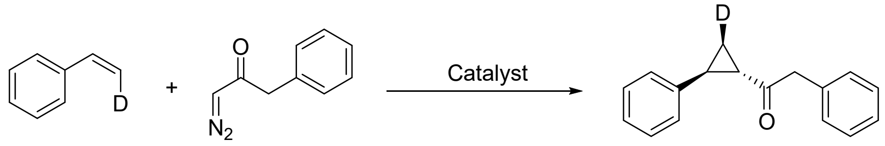

***cis*-*d*-1** **2** ***d*-3**

Entry	Catalyst	Yield[a]	Ratio[b]	% Isomerization
1	Mb (H64G, V68A)	32%[c]	1:0	0%
2	Fe(TPP)Cl	23%[d]	1:0	0%
3	Co(TPP)	8%[e]	1:0	0%
4	Hemin	8%[f]	1:0	0%

Supplementary Results

Computational Studies – Additional Results and Discussion

Mössbauer property calculations. To help characterize the electronic structure of the protein species isolated in the Mössbauer experiments and as general reference, a quantum chemical investigation of different heme complexes in different spin states was conducted using a previously established method which has provided accurate predictions for >50 experimental systems[3-17] (see **Computational Methods**). For assignment purposes, the structural models were further evaluated by reduced χ^2 analysis and Bayesian probability (or Z-surface) technique[17,18] using experimental values for both Mössbauer isomer shift and quadrupole splitting parameters (see **Computational Methods**). A small χ^2 value indicates a small deviation from experiment and a large Z value means a high probability of the model corresponding to the experimental system.

As summarized in **Supplementary Table 2**, for Mössbauer species **A**, which was obtained experimentally from a sample of **Mb-imi** treated with sodium dithionite (**Figure 2**), both five-coordinate and six-coordinate iron porphyrin complexes with the sixth ligand being H_2O, HO^-, and Im were studied. The calculated systems cover both ferrous and ferric oxidation states. Except for [Fe^{II}(Por)(His)] and [Fe^{III}(Por)(His)(H_2O)] which has experimentally determined spin states of S=2 and 5/2 respectively,[6,7] all other systems were studied with all possible spin states (i.e. 0, 1, and 2 for ferrous complexes and ½, 3/2, 5/2 for ferric complexes). These results showed an excellent agreement between the experimental values and the calculated Mössbauer isomer shift and quadrupole splitting parameters for the singlet (S=0) [Fe^{II}(Por)(His)(Im)] complex, which was also found to be the ground state (see Supplementary Table 2 footnote). A good match also corresponded to [Fe^{III}(Por)(His)(H_2O)] (S=5/2), but this assignment was ruled out based on the UV-vis analysis of the protein sample recorded prior to flash-freezing for Mössbauer analysis,

which indicated quantitative reduction of the protein under the applied conditions (**Supplementary Figure 3**). Although the ferrous and ferric complexes of hydroxide axial ligands with intermediate spin states also have similar prediction errors, these intermediate states are of the highest energies among their possible spin states (Supplementary Table 2 footnote)

For species **B**, corresponding to the trapped green species as shown in **Figure 2**, structural models of both the N-alkylated heme complex and the bridging heme-carbene with S = 0, 1, and 2 were investigated. As shown in **Supplementary Table 2**, both complexes in their quintet state (S=2), which corresponds to the lowest energy spin state, exhibit a best match between their calculated Mössbauer parameters and the experimental values. These two models have similar χ^2 and Z data (**Supplementary Table 2**). An HO^- coordinated N-alkylated heme complex ($\mathbf{P_{N\text{-}AlkylatedOH\text{-}}}$) was also studied but show no match with experimental value. Thus, assignment of Mössbauer species B to **Mb-cIII** was made on the basis of these data along with our crystallographic data and the matching UV-vis spectra (Q band region) for this species as determined in solution and *in crystallo* (**Figure 2**).

Alternative heme-bound carbene formation pathways in the presence of diazoketone. In addition to heme-carbene (IPC) formation via the C-coordinated diazo intermediate (**Supplementary Figure 12**), other possible pathways were analyzed for formation of the heme-bound carbene intermediate.

Uncatalyzed carbene formation pathway

For reference, the Gibbs free energy profile for the uncatalyzed free carbene formation from BDK was calculated. These calculations indicated that, in the absence of the heme catalyst, the Gibbs free energy of activation ($\Delta G^{\ddagger}$) for free carbene formation via nitrogen extrusion is 34.40

kcal/mol (Figure 2), which is 10.0 kcal/mol higher than in the presence of the heme catalyst. In addition, calculated energy of the free carbene is 25.3 kcal/mol which is higher than that of the IPC (**Supplementary Figure 14**) by ~23 kcal/mol. So, both kinetic and thermodynamic results strongly support the role of the heme catalyst in facilitating carbene formation.

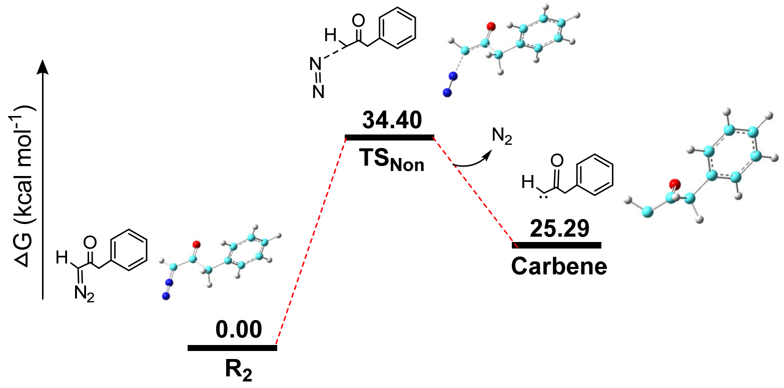

Supplementary Figure 14. Gibbs free energy diagram for the uncatalyzed pathway for free carbene formation.

Heme carbene formation via N-coordination pathway

As summarized in **Supplementary Figure 15**, a reaction pathway for IPC formation proceeding via heme binding of diazoketone (BDK) through a terminal-N coordination mode was investigated. For the first intermediate **Int$_{N1}$**, although its quintet is 11.21 kcal/mol lower in energy than the singlet (**Supplementary Table 11**), only singlet state shows a reasonable coordination bond between Fe and the terminal N atom in diazoketone (Nt) with 1.962 Å (**Supplementary Figure 15**). In contrast, the same distance is about 6Å in the case of the quintet form, indicating no interaction between the diazoketone compound and the heme iron center (**Supplementary Figure 15**). This suggests that this pathway first undergoes a spin crossover from the reactant's

quintet to the singlet coordinated mode, ^{1}**Int$_{N1}$**, which is higher in energy by 8.55 kcal/mol. After the carbene formation transition state (**TS$_N$**), C-N_b (C is carbene's carbon and N_b is bridging nitrogen in diazoketone) bond breaks and carbene group is released to generate **Int$_{N2}$**. Spin state calculations show that ^{1}**Int$_{N2}$** is 7.03 kcal/mol higher in energy than its quintet state (**Supplementary Table 11**). However, as for **Int$_{N1}$**, only the singlet state of **Int$_{N2}$** maintains a coordination bond with the heme iron ($R_{Fe\cdots Nt}$ = 1.973 Å, **Figure 15**), whereas a much larger distance ($R_{Fe\cdots Nt}$ = 3.526 Å) and thus weaker iron binding interaction is observed for the quintet state in quintet. So, it can be derived that once ^{1}**Int$_{N2}$** is formed, it will release ~7 kcal/mol energy to produce the loosely bound ^{5}**Int$_{N2}$**, which then decomposes to regenerate quintet **R$_{heme}$** and release the free carbene. The free carbene can subsequently rebind with heme center to yield **Int$_{IPC}$**. Since only singlet **Int$_{N1}$** and **Int$_{N2}$** have coordination bonds, these results suggest that the N-coordinated transition state for carbene formation, **TS$_N$**, also occurs at S=0, which shall be higher than 36.38 kcal/mol, i.e., the higher energy of ^{1}**Int$_{N2}$**. Thus, the energy barrier associated with **TS$_N$** (i.e., carbene formation via N-coordination) is higher than that calculated for the uncatalyzed pathway described in **Supplementary Figure 14**. Compared to the $\Delta G^{\ddagger}$ of 24.38 kcal/mol for heme carbene formation via the C-coordination mode in **Supplementary Figure 12**, even the energy for the quintet intermediate ^{5}**Int$_{N2}$** remain ~ 5 kcal/mol higher (**Supplementary Table 12**). In addition to the higher energy cost, this pathway also involves two additional steps compared to the heme carbene formation mechanism via C-coordination. Overall, these results indicate that the heme carbene formation via the N-coordination pathway is not favorable compared to the pathway proceeding via C-coordination (**Supplementary Figure 12**).

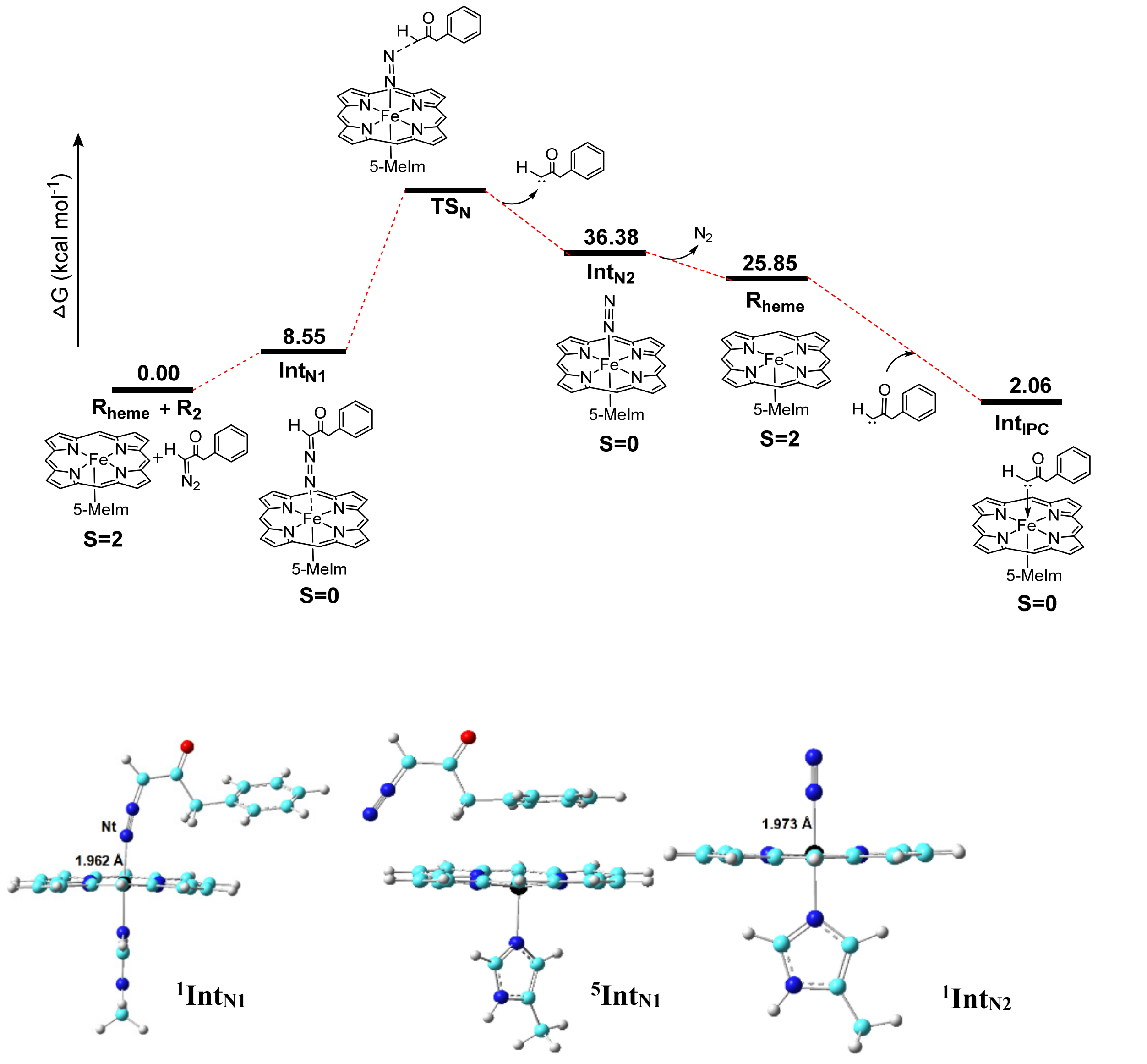

Supplementary Figure 15. Gibbs free energy diagram for the heme-bound carbene formation pathway with the N-coordination of diazoketone and optimized structures for key species in this pathway.

Heme carbene formation via O-coordination pathway

A pathway for heme carbene proceeding via heme coordination by the carbonyl group of the diazoketone was also investigated (**Supplementary Figure 16-S17)**. In contrast with the C-coordination and N-coordination modes, the O-coordinated intermediate **Int$_{O1}$** was found to keep

the Fe-O coordination in both singlet and quintet state, as indicated by calculated Fe···O distances of 2.068 and 2.458 Å, respectively (**Supplementary Figure 16A-B**). While these interactions are weak, they are similar to that for ^{1}**Int$_{C1}$** (Fe···C distance: 2.425 Å) in the C-coordination pathway (**Supplementary Table 8**). As in the C- and N-coordination intermediates, the quintet state ^{5}**Int$_{O1}$** is more favorable its quintet state ^{1}**Int$_{O1}$**, being 3.73 kcal/mol lower energy (**Supplementary Table 13**). So, this pathway is also posed to undergo spin crossover from the reactant's quintet to the singlet coordinated mode, ^{1}**Int$_{O1}$**, which lies only 4.89 kcal/mol higher in energy compared to the reactants. After the carbene formation transition state (**TS$_{O}$**), C-N$_{b}$ bond breaks and free carbene is released to generate **Int$_{O2}$**. For this intermediate, unusual results appeared in initial trials. For instance, calculations with the initial single Fe^{II} feature led to the formation of a three-membered ring involving carbene C atom and C=O group. A more extensive examination using both the initial Fe^{II} and Fe^{III} features, with both ferromagnetic and anti-ferromagnetic spin interactions with the carbene part for the overall S = 0, 1, 2, was carried out in order to identify stable forms for O-coordinated **Int$_{O2}$** as well as **Int$_{O1}$**.

Interestingly, for **Int$_{O1}$**, all trials ended up with the same Fe^{II} feature and structures as in the first round of calculations for singlet and quintet **Int$_{O1}$**, and the triplet **Int$_{O1}$** also retains the Fe^{II} feature (spin density $\rho_{\alpha\beta}^{Fe}$ = 2.011 e) but has no Fe-O coordination bond (Fe…O distance: 2.771 Å, see **Figure S16C**). For **Int$_{O2}$**, however, this search found the following structures with Fe-O coordination: 1) for the singlet state, the open-shell state with basically Fe^{III} (S=1/2) anti-ferromagnetically coupled with C (S=-1/2) (**Supplementary Table 13**) has Fe-O bond length of 1.847 Å (**Supplementary Figure 16D**); 2) for the triplet state, among the two optimized structures, the one with the Fe^{III} feature (S=1/2, ferromagnetically coupled with carbene radical) has the lowest energy (-1.73 kcal/mol, compared to singlet, **Supplementary Table 13** and

Supplementary Figure 16E), while the one with the Fe^{II} feature (S=0) and triplet carbene has the highest energy (3.05 kcal/mol relative singlet, **Supplementary Table 13** and **Supplementary Figure 16F**); 3) for the quintet state, both optimized structures have the ferric feature, with anti-ferromagnetic and ferromagnetic couplings to carbene respectively and corresponding Fe-O bond lengths of 1.884 and 1.949 Å (**Figure S16G-H**). Overall, it is concluded that this intermediate has many Fe^{III}-based states and the most favorable one is the triplet state with the Fe^{III} radical and carbene radical ferromagnetically coupled to it. This is not entirely surprising since heme coordination of the electronegative O atom after the departure of the N_2 moiety, could promote electron transfer from the original Fe^{II} center of $\mathbf{Int_{O1}}$ to the carbene moiety, resulting in this electronic configuration. This favorable electronic feature results in $\mathbf{Int_{O2}}$ having a ΔG of 22.25 kcal/mol (**Supplementary Figure 17**), which is 14 kcal/mol more stable than the second intermediate $^{1}\mathbf{Int_{N2}}$ in the N-coordination pathway ($\Delta G(^{1}\mathbf{Int_{N2}})$ = 36.38 kcal/mol, **Supplementary Figure 15**).

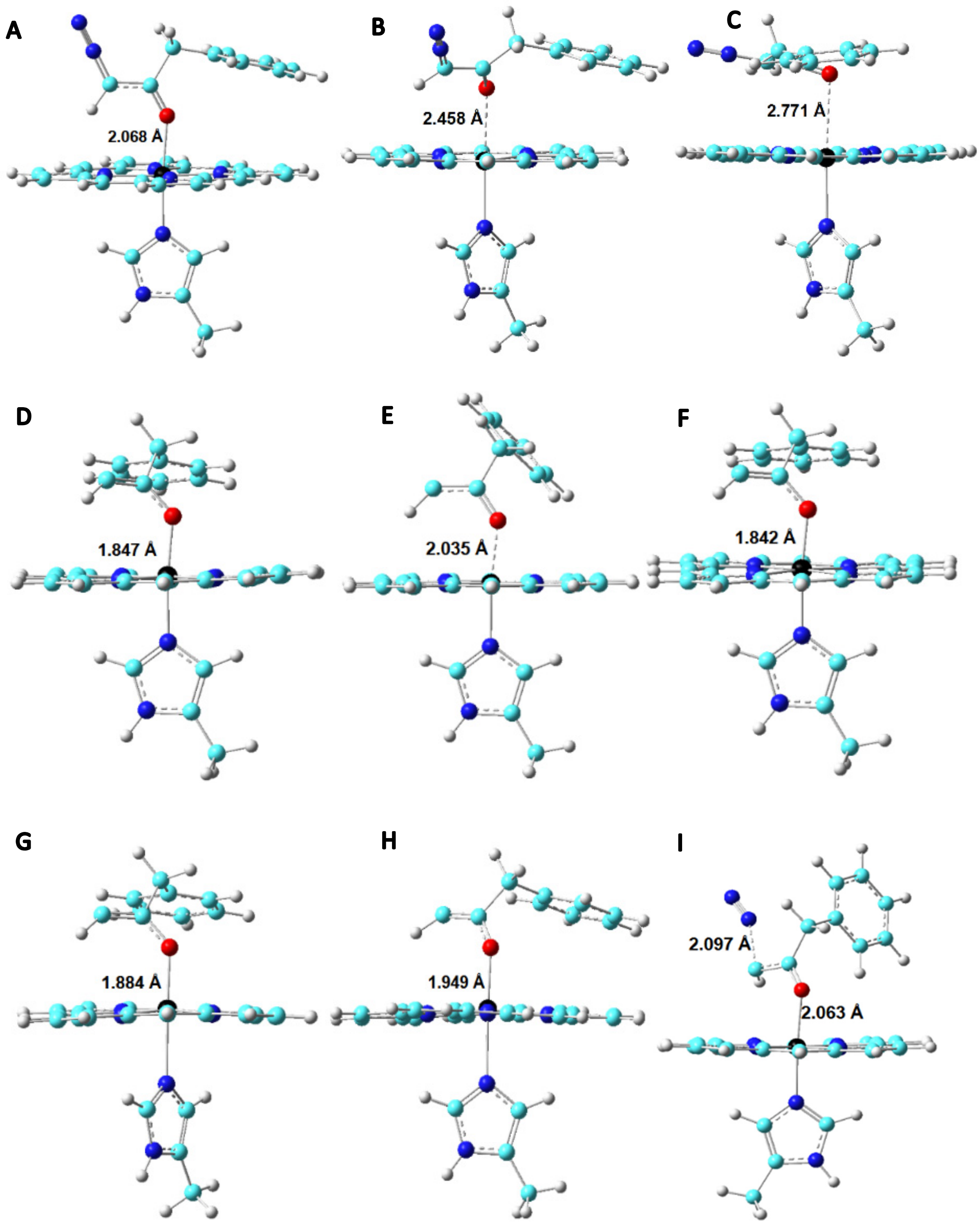
A
2.068 Å
B
2.458 Å
C
2.771 Å
D
1.847 Å
E
2.035 Å
F
1.842 Å
G
1.884 Å
H
1.949 Å
I
2.097 Å
2.063 Å

Supplementary Figure 16. Optimized intermediates for the heme carbene formation pathway via O-coordination: **A)** Int_{O1} (S=0); **B)** Int_{O1} (S=2); **C)** Int_{O1} (S=1); **D)** Int_{O2} (S=0); **E)** Int_{O2} (S=1); **F)** another Int_{O2} (S=1); **G)** Int_{O2} (S=2); **H)** another Int_{O2} (S=2); **I)** TS_O (S=0).

Since (1) there is the electronic state change from singlet for $\mathbf{Int_{O1}}$ to biradical triplet for $\mathbf{Int_{O2}}$; (2) $\mathbf{Int_{O1}}$ does not have any sSupplementary Table tate with the radical feature, and (3) $\mathbf{Int_{O2}}$ does not have any sSupplementary Table tate with the non-radical feature (**Supplementary Table 13**), there may be additional steps than just a single $\mathbf{TS_O}$ to connect these two intermediates and to account for both geometric and electronic state changes. For example, after the singlet $\mathbf{TS_O}$ from singlet $\mathbf{Int_{O1}}$, a spin crossover from singlet to biradical triplet is required to reach the stable $\mathbf{Int_{O2}}$ intermediate. For this scenario, the singlet $\mathbf{TS_O}$ has been successfully located at a $\Delta G^{\ddagger}$ of 40.37 kcal/mol (**Supplementary Table 14**), as illustrated in **Supplementary Figure 16I**. The alternative scenario involving a spin crossover from singlet to biradical triplet for $\mathbf{Int_{O1}}$ first and then formation of a biradical $\mathbf{TS_O}$ to reach the stable $\mathbf{Int_{O2}}$ with the same spin feature seems unlikely due to failure in locating biradical triplet $\mathbf{Int_{O1}}$ and $\mathbf{TS_O}$ after several attempts. The $\Delta G^{\ddagger}$ of 40.37 kcal/mol for the pathway proceeding via singlet $\mathbf{TS_O}$ is much higher than the barrier for the C-coordination heme carbene formation pathway (24.38 kcal/mol). In addition, the subsequent step involving decomposition of $\mathbf{Int_{O2}}$ to regenerate the ground state $\mathbf{R_{heme}}$ and release the free carbene, which can subsequently rebind to the heme center to yield $\mathbf{Int_{IPC}}$, is ca. 3 kcal/mol uphill in energy (**Supplementary Figure 17**). Overall, these unfavorable energies make it less kinetically favorable than heme carbene formation process via the C-coordination pathway.

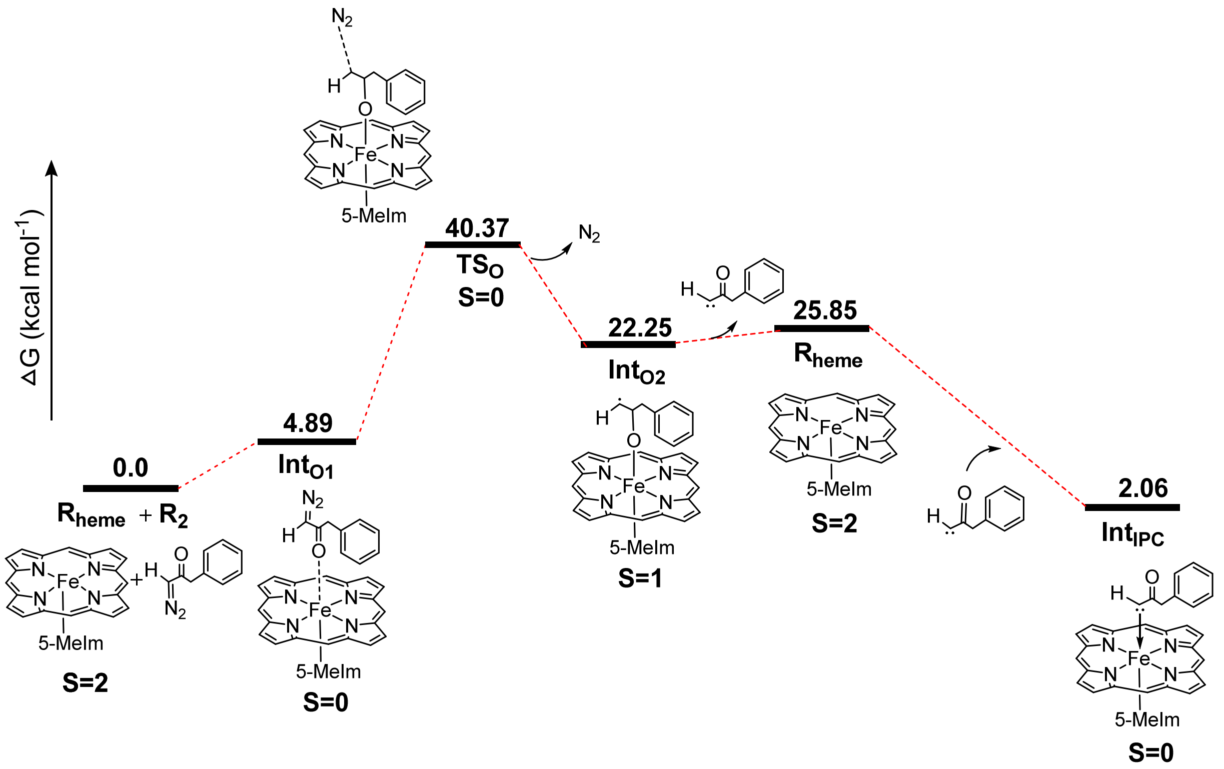

Supplementary Figure 17. Gibbs free energy diagram for the heme carbene formation pathway with the O-coordination of diazoketone.

Bridging heme-carbene and N-alkylated heme complexes. To investigate potential pathways for formation of **Mb-cIII** (here modeled as $\mathbf{P_{N\text{-}alkylation}}$) from the heme-carbene intermediate ($\mathbf{Int_{IPC}}$), we began by optimizing a bridging Fe-C-N heme-carbene complex with a singlet, triplet, and quintet state. As shown in **Supplementary Figure 18**, all the spin states were found to be able to adopt the Fe-C-N bridging carbene configuration, with a relatively longer C–Fe bond and shorter C–N bond lengths going from S=2 to S=0. The quintet has the lowest energy and has the most significant heme plane distortion due to the shortest C–N bond and thus strongest perturbation to the porphyrin structure by the bridging carbene group. Interestingly, the computed energies for these complexes are all lower than that of the terminally bound carbene

Int$_{IPC}$ (**Supplementary Table 15**), showing a thermodynamically favorable process for the structural change from **Int$_{IPC}$**.

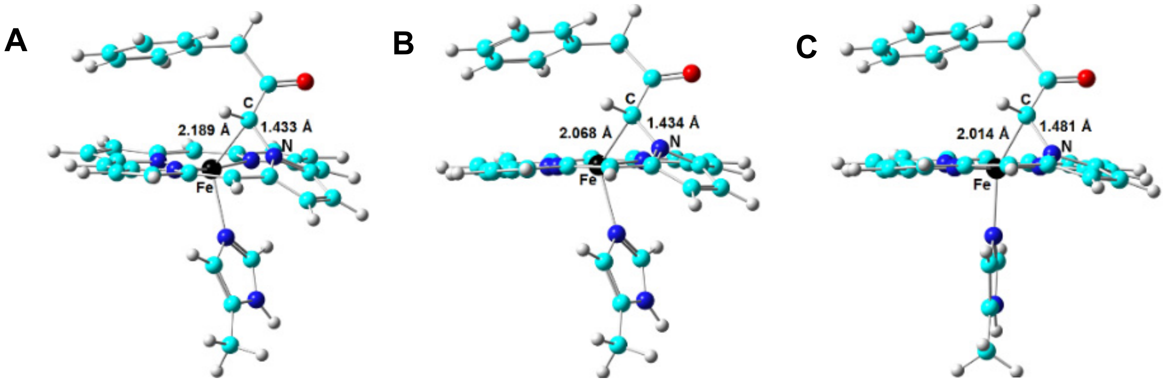

Supplementary Figure 18. Optimized structures for the bridging heme carbene complexes with BDK-derived carbenes corresponding to S=2 (**A**), S=1 (**B**), and S=0 (**C**).

Next, we optimized the N-alkylation product (**P$_{N\text{-alkylation}}$**) in the singlet, triplet, and quintet state. Fe^{III} (S=1/2, 3/2, 5/2) anti-ferromagnetically coupled with carbene (S = ±1/2) were also considered but all of them turn out to become Fe^{II} after optimization. The final optimized structures are shown in **Supplementary Figure 19** for S=2, 1, 0 respectively. As for the bridging heme-carbene complex (**Supplementary Figure 18**), the quintet state has the lowest energy and it exhibits the most significant distortion of the heme plane due to the shortest C–N bond and the longest Fe–N distance among the different spin states and thus the strongest perturbation to the porphyrin structure by the [CH_2CO_2Et] group (**Supplementary Table 16**). Among all spin states, this quintet structure is also closest to the structure of the heme complex in **Mb-cIII** as determined via X-ray crystallography (**Figure 2**).

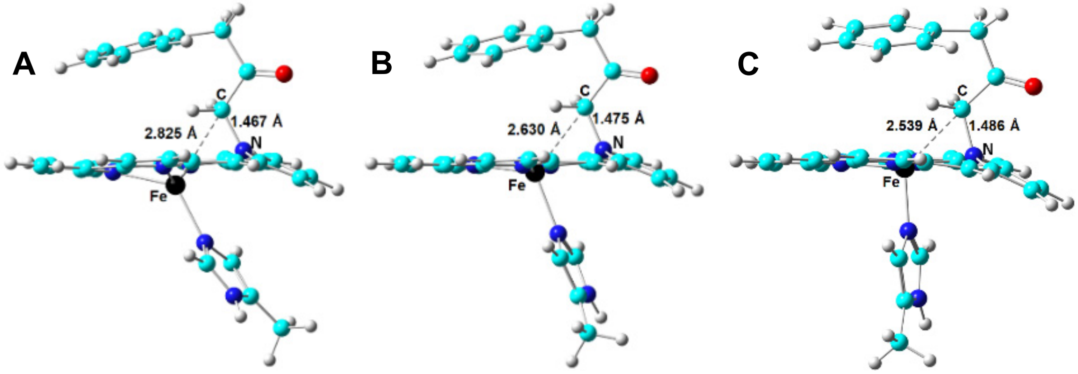

Supplementary Figure 19. Optimized structures for N-alkylated heme-carbene complexes corresponding to S=2 (**A**), S=1 (**B**), and S=0 (**C**).

The conversion of terminal iron porphyrin complex (**Int$_{\mathbf{IPC}}$**) to the bridging heme-carbene complex (**Int$_{\mathbf{IPC\text{-}Bridging}}$**) was also studied. The singlet transition state (**TS$_{\mathbf{Bridging}}$**) is significantly more favorable than the quintet (**Supplementary Table 17**), which is consistent with a previous study on a related iron porphyrin carbene complex with EDA as carbene donor.[19] As shown in **Supplementary Figure 20** and **Supplementary Table 18**, the energy barrier for formation of the bridging heme-carbene complex from the terminal IPC is $\Delta G^{\ddagger}$ 4.07 kcal/mol, suggesting a facile conversion from the former to the latter. After reacting with a proton source such as H_3O^+, the **Int$_{\mathbf{IPC\text{-}Bridging}}$** species can undergo protonation to give the **Mb-cIII** complex (modeled as **P$_{\mathbf{N\text{-}alkylation}}$**). The path leading from **Int$_{\mathbf{IPC}}$** to **Mb-cIII** is thermodynamically very favorable (ΔG = -89.4 kcal/mol) and such strong thermodynamic driving force make the protonation process from **Int$_{\mathbf{IPC\text{-}Bridging}}$** to **Mb-cIII** barrierless via first the protonated and water bound intermediate **Int$_{\mathbf{N\text{-}AlkylationOH2}}$** and then water dissociated **P$_{\mathbf{N\text{-}Alkylation}}$**, which undergoes a spin state change to its favorable quintet, see **Supplementary Figure 20**. Altogether, these calculations indicated that formation of the N-alkylated heme complex, corresponding to the experimentally characterized

protein complex **Mb-cIII**, is kinetically feasible and thermodynamically favorable starting from the heme-carbene (IPC) complex.

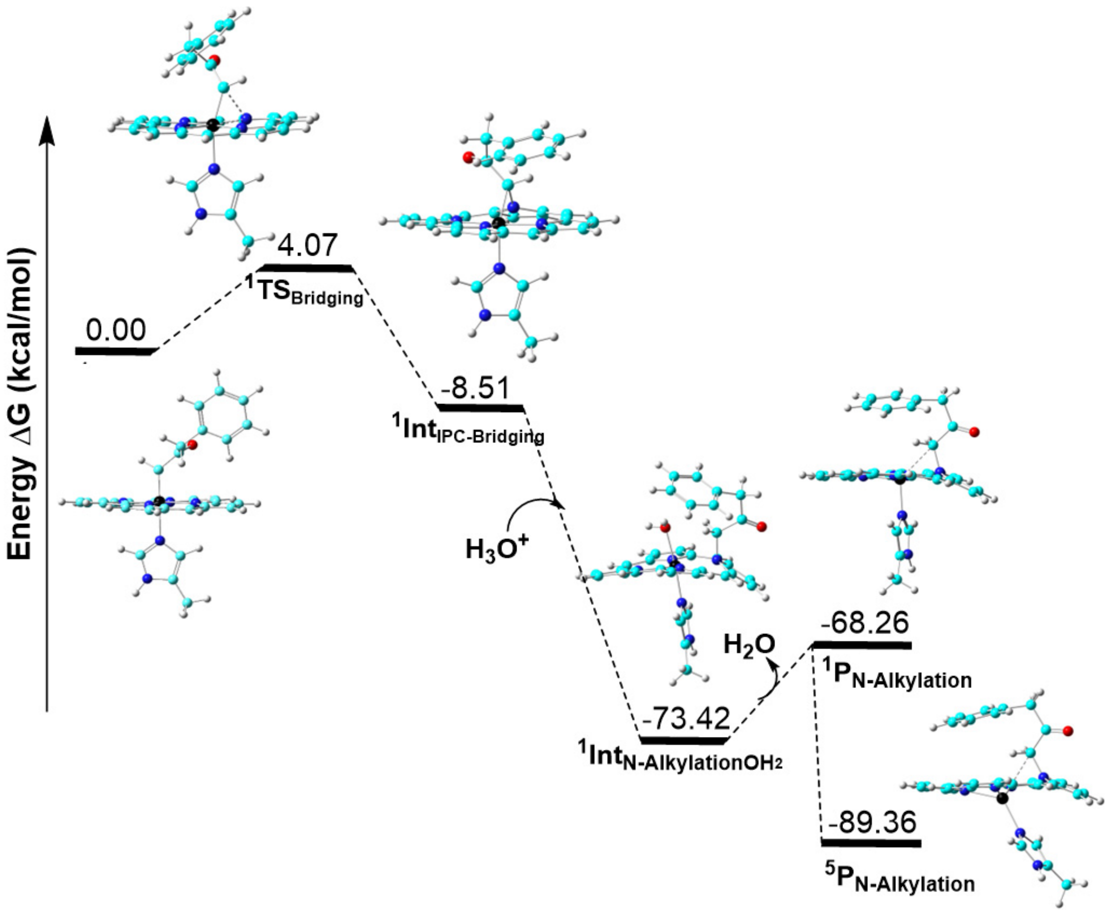

Supplementary Figure 20. Gibbs free energy diagram for the N-alkylated product formation pathway from terminal iron porphyrin carbene.

Like the N-alkylated heme complex, the bridging heme-carbene was found to be unable to affect the cyclopropanation reaction, since all attempts to optimize the corresponding transition state with either concerted or stepwise mechanisms ended up with dissociated styrene and the bridging carbene. This is probably a result of (i) the short C–N bond and the bent pyrrole ring in the bridging carbene group (**Supplementary Figure 18**), which is likely to prevent substrate

approaching due to steric hindrance with the porphyrin; and (ii) the carbene C atom being bonded with four atoms to achieve full valence, which makes it unreactive toward attack by the olefin. The unfavorable cyclopropanation reactivity of the bridging heme-carbene complex as determined here is consistent with previous results on similar metalloporphyrin complexes.[20]

Supplementary Tables with Computational Data

Supplementary Table 5. Relative Spin State Energies for Species in the Productive C-coordination Pathway (unit: Spin Densities in e and Energies in kcal/mol)

Species	S	$\rho_{\alpha\beta}^{Fe}$	$\rho_{\alpha\beta}^{C}$	$\rho_{\alpha\beta}^{C1}$	$\rho_{\alpha\beta}^{C2}$	$\rho_{\alpha\beta}^{R'}$	$\rho_{\alpha\beta}^{L}$	$\rho_{\alpha\beta}^{Por}$	ΔE	ΔE_{ZPE}	ΔH	ΔG
$^{1}\mathbf{Int_{C1}}$	0	0.000	0.000				0.000	0.000	0.00	0.00	0.00	0.00
$^{5}\mathbf{Int_{C1}}$	2	3.762	-0.013				0.045	0.195	-2.36	-4.65	-3.73	-8.74
$^{1}\mathbf{TS_{Carbene}}$	0	0.000	0.000				0.000	0.000	0.00	0.00	0.00	0.00
$^{5}\mathbf{TS_{Carbene}}$	2	3.772	-0.035				0.032	0.204	13.67	10.48	11.56	7.16
$^{1}\mathbf{Int_{IPC}}$	0	0.000	0.000				0.000	0.000	0.00	0.00	0.00	0.00
$^{5}\mathbf{Int_{IPC}}$	2	3.117	0.485				0.004	0.197	15.89	14.42	14.82	13.20
$^{1}\mathbf{TS_{CP}}$	0	0.000	0.000	0.000	0.000	0.000	0.000	0.000	0.00	0.00	0.00	0.00
$^{5}\mathbf{TS_{CP}}$	2	3.911	-0.240	0.052	-0.079	-0.011	0.035	0.287	15.05	11.76	12.83	8.63

[a] C is carbene's carbon. L is axial ligand 5-MeIm. H is the hydrogen bonded with C in [:$CHCOCH_2Ph$]. C1 is terminal carbon on styrene and C2 is the carbon next to C1 on styrene. R' is the remaining part of styrene without C1 and C2. Superscript on the left of species indicates spin state.

Supplementary Table 6. Absolute Energies of Selected Species in the Studied Reaction Pathways (unit: Hartrees)

Species	E	E_{ZPE}	H	G
$^{5}\mathbf{R_{heme}}$	-1377.28134	-1376.90483	-1376.87936	-1376.96134
$^{1}\mathbf{R_{2}}$	-532.38095	-532.22696	-532.21557	-532.26519
$^{1}\mathbf{Int_{C1}}$	-1909.68157	-1909.14590	-1909.10981	-1909.21229
$^{1}\mathbf{TS_{Carbene}}$	-1909.65317	-1909.12024	-1909.08403	-1909.18768
$^{1}\mathbf{Int_{IPC}}$	-1800.15434	-1799.62931	-1799.59516	-1799.69512
$^{1}\mathbf{N_{2}}$	-109.51538	-109.50970	-109.50639	-109.52813
$^{1}\mathbf{Styrene}$	-309.59820	-309.46392	-309.45625	-309.49539
$^{1}\mathbf{TS_{CP}}$	-2109.76299	-2109.10086	-2109.05980	-2109.17507
$^{1}\mathbf{P_{CP}}$	-732.55478	-732.27051	-732.25448	-732.31590
$^{1}\mathbf{TS_{non}}$	-532.31904	-532.17027	-532.15784	-532.21037
$^{1}\mathbf{Carbene}$	-422.80239	-422.66116	-422.65123	-422.69677
$^{1}\mathbf{Int_{N1}}$	-1909.68045	-1909.14403	-1909.10804	-1909.21290
$^{1}\mathbf{Int_{N2}}$	-1486.80494	-1486.41659	-1486.38956	-1486.47179
$^{1}\mathbf{Int_{O1}}$	-1909.68405	-1909.14876	-1909.11251	-1909.21873
$^{1}\mathbf{TS_{O}}$	-1909.62509	-1909.09380	-1909.05713	-1909.16219
$^{3}\mathbf{Int_{O2}}$	-1800.12297	-1799.59830	-1799.56441	-1799.66294
$^{1}\mathbf{TS_{Bridging}}$	-1800.15021	-1799.62536	-1799.59224	-1799.68865
$^{1}\mathbf{Int_{IPC-Bridging}}$	-1800.17391	-1799.64628	-1799.61314	-1799.70869
$^{5}\mathbf{Int_{IPC-Bridging}}$	-1800.18693	-1799.66257	-1799.62833	-1799.72896
$\mathbf{H_2O}$	-76.41545	-76.39368	-76.38990	-76.41196
$\mathbf{H_3O^+}$	-76.78806	-76.75214	-76.74831	-76.77125
$^{1}\mathbf{Int_{N-AlkylatedOH2}}$	-1877.08578	-1876.51788	-1876.48202	-1876.58338
$^{1}\mathbf{P_{N-Alkylation}}$	-1800.64076	-1800.09954	-1800.06592	-1800.16320
$^{5}\mathbf{TS_{N-Alkylated}}$	-1876.58503	-1876.03996	-1876.00368	-1876.10991

Supplementary Table 7. Relative Energies of Species in the Productive C-coordination Reaction Pathway (unit: kcal/mol)

Species	ΔE	ΔE_{ZPE}	ΔH	ΔG
^{5}**R$_{heme}$** +^{1}**R$_2$**	0.00	0.00	0.00	0.00
^{5}**Int$_{C1}$**	-14.46	-13.50	-13.07	0.19
^{1}**Int$_{C1}$**	-12.10	-8.85	-9.34	8.93
^{1}**TS$_{Carbene}$**	5.72	7.25	6.84	24.38
^{1}**Int$_{IPC}$**+^{1}**N$_2$**	-4.66	-4.53	-4.16	2.06
^{1}**TS$_{CP}$**	-11.22	-9.31	-9.42	11.75
^{5}**R$_{heme}$**+^{1}**P$_{CP}$**	-57.11	-56.06	-55.88	-52.36

Supplementary Table 8. Selected Geometric Parameters of Species in the Productive C-coordination Reaction Pathway [a]

Species	R_{FeC}	R_{FeL}	R_{CH}	$R_{C1C'}$	R_{CN_b}	$R_{N_bN_t}$	R_{CC1}	R_{CC2}	R_{C1C2}	$\angle_{HCC'}$	$\angle_{CN_bN_t}$
^{5}**R$_{heme}$**		2.167									
^{1}**R$_2$**			1.080	1.530	1.306	1.123				122.7	179.0
^{5}**Int$_{C1}$**	3.097	2.163	1.080	1.457	1.307	1.122				122.6	178.8
^{1}**Int$_{C1}$**	2.425	1.996	1.082	1.477	1.327	1.114				119.5	176.9
^{1}**TS$_{Carbene}$**	1.957	2.055	1.093	1.492	1.848	1.094				110.9	161.7
^{1}**Int$_{IPC}$**	1.757	2.130	1.099	1.476						112.5	
^{1}**N$_2$**						1.092					
^{1}**Styrene**									1.331		
^{1}**TS$_{CP}$**	1.893	2.079	1.092	1.494			2.337	2.647	1.347	109.5	
^{1}**P$_{CP}$**			1.084	1.488			1.509	1.531	1.495	112.2	

[a] Units of bond lengths and angles are Å and degrees, respectively. C' is carbonyl carbon. H is the hydrogen bonded with carbene in [:$CHCOCH_2Ph$]. N_b and N_t are terminal and bridging nitrogen atoms in the diazoketone. C1 is the terminal carbon on styrene and C2 is the carbon next to C1 on styrene.

Supplementary Table 9. Atomic Charges of Species in the Productive C-coordination Reaction Pathway (unit: *e*)

Species	Q_{Fe}	Q_C	Q_H	$Q_{C'}$	Q_{N_b}	Q_{N_t}	Q_{C1}	Q_{C2}	$Q_{R'}$	Q_L	Q_{Por}
^{5}**R$_{heme}$**	1.118									0.158	-1.276
^{1}**R$_2$**		-0.306	0.251	-0.072	0.096	0.032					
^{5}**Int$_{C1}$**	1.090	-0.317	0.267	-0.071	0.111	0.036				0.162	-1.278
^{1}**Int$_{C1}$**	0.403	-0.355	0.287	-0.016	0.146	0.090				0.302	-0.856
^{1}**TS$_{Carbene}$**	0.172	-0.128	0.219	-0.022	0.098	0.143				0.273	-0.754
^{1}**Int$_{IPC}$**	0.092	0.097	0.195	-0.045						0.245	-0.584
^{1}**N$_2$**					0.000	0.000					
^{1}**Styrene**							-0.349	-0.200	0.549		
^{1}**TS$_{CP}$**	0.163	-0.071	0.202	-0.047			-0.346	-0.139	0.670	0.266	-0.699
^{1}**P$_{CP}$**		-0.304	0.233	-0.001			-0.364	-0.207	0.643		

Supplementary Table 10. Relative Energies of Species in the Non-catalyzed Pathway (unit: kcal/mol)

Species	ΔE	ΔE_{ZPE}	ΔH	ΔG
^{1}**R$_2$**	0.00	0.00	0.00	0.00
^{1}**TS$_{non}$**	38.85	35.58	36.23	34.40
^{1}**N$_2$**+^{1}**Carbene**	39.64	35.21	36.36	25.29

Supplementary Table 11. Key Geometric Parameter, Spin Densities, and Relative Spin State Energies of N-coordination Intermediates (unit: Bond Length in Å, Spin Densities in e and Energies in kcal/mol)

Species	S	R_{FeNt}	$\rho_{\alpha\beta}^{Fe}$	$\rho_{\alpha\beta}^{C}$	$\rho_{\alpha\beta}^{L}$	$\rho_{\alpha\beta}^{Por}$	ΔE	ΔE_{ZPE}	ΔH	ΔG
**Int_{N1}**	0	1.962	0.000	0.000	0.000	0.000	0.00	0.00	0.00	0.00
	2	6.036	3.758	0.000	0.044	0.197	-6.16	-8.85	-7.88	-11.21
**Int_{N2}**	0	1.973	0.000	0.000	0.000	0.000	0.00	0.00	0.00	0.00
	2	3.489	3.743	0.000	0.043	0.214	1.34	-2.07	-0.48	-8.05

Supplementary Table 12. Relative Energies of Species (unit: kcal/mol) in the N-coordination Pathway

Species	ΔE	ΔE_{ZPE}	ΔH	ΔG
**$^5R_{heme}$ +$^1R_2$**	0.00	0.00	0.00	0.00
**$^1Int_{N1}$**	-11.40	-7.68	-8.23	8.55
**$^1Int_{N2}$+$^1N_2$**	34.48	33.91	33.97	36.38
**$^5R_{heme}$+$^1N_2$+^{1}Carbene**	39.58	35.40	36.49	25.85
**$^1Int_{IPC}$+$^1N_2$**	-4.66	-4.53	-4.16	2.06

Supplementary Table 13. Key Geometric Parameter, Spin Densities, and Relative Spin State Energies of O-coordination Intermediates (unit: Bond Length in Å, Spin Densities in e, and Energies in kcal/mol)

Species	S	R_{FeO}	$\rho_{\alpha\beta}^{Fe}$	$\rho_{\alpha\beta}^{C}$	$\rho_{\alpha\beta}^{L}$	$\rho_{\alpha\beta}^{Por}$	ΔE	ΔE_{ZPE}	ΔH	ΔG
**Int_{O1}**	0	2.068	0.000	0.000	0.000	0.000	0.00	0.00	0.00	0.00
	1	2.771	2.011	-0.001	0.004	-0.053	2.12	1.30	1.86	-0.02
	2	2.458	3.769	0.000	0.032	0.188	0.59	-1.63	-0.80	-3.73
**Int_{O2}**	0	1.847	0.976	-1.023	-0.004	-0.055	0.00	0.00	0.00	0.00
	1	2.035	0.057	1.721	-0.001	0.004	5.42	4.71	4.93	3.05
	1	1.842	0.919	1.115	-0.006	-0.039	-0.49	-0.59	-0.52	-1.73
	2	1.884	4.143	-0.981	0.063	0.457	6.51	4.32	5.09	1.23
	2	1.949	2.741	1.133	0.055	-0.063	7.02	5.56	6.29	2.09

Supplementary Table 14. Relative Energies of Species (unit: kcal/mol) in the O-coordination Pathway

Species	ΔE	ΔE_{ZPE}	ΔH	ΔG
**$^5R_{heme}$ +$^1R_2$**	0.00	0.00	0.00	0.00
**$^1Int_{O1}$**	-13.66	-10.64	-11.03	4.89
**^1TS_O**	23.34	23.84	23.72	40.37
**$^3Int_{O2}$+$^1N_2$**	15.02	14.93	15.14	22.25
**$^5R_{heme}$+$^1N_2$+^{1}Carbene**	39.58	35.40	36.49	25.85
**$^1Int_{IPC}$+$^1N_2$**	-4.66	-4.53	-4.16	2.06

Supplementary Table 15. Selected Geometric Parameters (unit: Bond Length in Å, Angle in degrees), Spin Densities (unit: e) and Relative energies [a] (unit: kcal/mol) of the Bridging Binding Carbene

S	R_{FeC}	$R_{FeN_{His}}$	$R_{FeN_{Por}}$	R_{CN}	$\angle_{FeCN}$	$\rho_{\alpha\beta}{}^{Fe}$	$\rho_{\alpha\beta}{}^{Por}$	$\rho_{\alpha\beta}{}^{Sub}$	ΔE	ΔE_{ZPE}	ΔH	ΔG
2[a]	2.189	2.175	2.440	1.433	81.8	3.649	0.231	0.079	-20.45	-20.87	-20.81	-21.23
1	2.068	2.153	2.507	1.434	89.5	1.939	0.037	-0.008	-18.12	-17.28	-17.65	-16.41
0	2.014	2.013	2.191	1.481	76.0	0.000	0.000	0.000	-12.29	-10.65	-11.28	-8.51

a) with respect to singlet **Int$_{IPC}$**

Supplementary Table 16. Selected geometric parameters (unit: Bond Length in Å, Angle in degrees), spin densities (unit: e) and relative energies [a] (unit: kcal/mol) of the **Mb-cIII** model **P$_{N\text{-}alkylation}$**.

S	R_{FeC}	$R_{FeN}{}^{b}$	$R_{FeN_P}{}^{c}$	$R_{FeHis}{}^{d}$	R_{CN}	$\angle_{FeCN}$	$\angle_{FeNC}$	$\angle_{NpFeNp}{}^{e}$	$\rho_{\alpha\beta}{}^{Fe}$	$\rho_{\alpha\beta}{}^{Por}$	$\rho_{\alpha\beta}{}^{Sub.}$	ΔE	ΔE_{ZPE}	ΔH	ΔG
Expt	*2.747*	*2.483*	*2.080*	*2.105*	*1.453*	*64.1*	*84.2*	*88.9*							
2	2.825	2.459	2.101	2.076	1.467	60.5	88.2	88.0	3.751	0.196	-0.002	0.00	0.00	0.00	0.00
1	2.630	2.327	2.005	2.094	1.475	61.7	84.3	90.8	1.959	-0.008	0.005	12.66	13.44	13.21	13.63
0	2.539	2.185	2.003	1.958	1.486	59.1	85.3	90.9	0.000	0.000	0.000	17.65	18.95	18.39	21.11

a) with respect to quintet N-alkylation product. b) N is the nitrogen bonded with bridging carbene carbon. c) N_P is one of the remaining three nitrogen atoms on the porphyrin ring. R_{FeN_P} is the average distance between Fe and N_P atoms. d) R_{FeHis} is the distance between Fe and the coordinated N atom in His residue (modeled as 5-methylimidazole here). e) $\angle_{NpFeNp}$ is the average of the two neighboring N_p-Fe-N_p angles for the three N_p atoms.

Supplementary Table 17. Key Geometric Parameter, Spin Densities, and Relative Spin State Energies of the Transition State in the Bridging Carbene Formation Pathway (unit: Bond Length in Å, Spin Densities in e and Energies in kcal/mol)

Species	S	R_{FeC}	R_{FeNPor}	$\rho_{\alpha\beta}{}^{Fe}$	$\rho_{\alpha\beta}{}^{C}$	$\rho_{\alpha\beta}{}^{L}$	$\rho_{\alpha\beta}{}^{Por}$	ΔE	ΔE_{ZPE}	ΔH	ΔG
**TS$_{Bridging}$**	0	1.826	2.030	0.000	0.000	0.000	0.000	0.00	0.00	0.00	0.00
	2	1.978	2.066	3.577	0.154	0.036	0.155	16.26	13.39	14.29	10.74

Supplementary Table 18. Relative Energies of Species in the Formation of Bridging Carbene and N-alkylated Product (unit: kcal/mol)

Species	ΔE	ΔE_{ZPE}	ΔH	ΔG
**^{1}Int$_{IPC}$**	0.00	0.00	0.00	0.00
**^{1}TS$_{Bridging}$**	2.59	2.48	1.83	4.07
**^{1}Int$_{IPC\text{-}Bridging}$ (+ OH_3^+)**	-12.29	-10.65	-11.28	-8.51
**^{1}Int$_{N\text{-}AlkylatedOH2}$**	-89.98	-85.61	-86.94	-73.42
**^{1}P$_{N\text{-}Alkylation}$ (+ H_2O)**	-71.43	-70.14	-70.50	-68.26
**^{5}P$_{N\text{-}Alkylation}$**	-89.08	-89.09	-88.89	-89.36

Supplementary Table 19. Selected Geometric Parameters of Species in the Formation of Bridging Carbene and N-alkylated Product [a]

Species	R_{FeC}	R_{FeNPor}	R_{FeL}	R_{CH}	$R_{CC'}$	R_{CO}	$R_{CH'}$	$R_{OH'}$	$\angle_{HCC'}$	$\angle_{CFeL}$	$\angle_{FeCC'O}$
**$^1TS_{Bridging}$**	1.826	2.030	2.049	1.094	1.469				112.5	158.2	-81.3
**$^1Int_{IPC\text{-}Bridging}$**	2.014	2.191	2.013	1.079	1.433				114.3	145.4	-87.2
**H_3O^+**								0.970			
**H_2O**								0.958			
**$^1Int_{N\text{-}AlkylatedOH2}$**	2.824	2.230	2.001	1.082	1.520	2.725	1.091	2.300	108.0	119.5	50.2
**$^1P_{N\text{-}Alkylation}$**	2.539	2.185	1.958	1.093	1.526				107.4	131.6	81.4
**$^5P_{N\text{-}Alkylation}$**	2.825	2.459	2.076	1.090	1.520				108.4	119.5	70.4

[a] Units of bond lengths and angles are Å and degrees, respectively. C' is carbonyl carbon. H is the hydrogen bonded with carbene in [:$CHCOCH_2Ph$]. O is the oxygen of H_2O. H′ is the hydrogen transferred from H_2O to C.

Supplementary Table 20. Atomic Charges of Species in the Formation of Bridging Carbene and N-alkylated Product (unit: *e*) [a]

Species	Q_{Fe}	Q_C	Q_H	Q_{COBn}	Q_L	Q_{Por}
**$^1TS_{Bridging}$**	0.152	-0.046	0.220	-0.056	0.272	-0.543
**$^1Int_{IPC\text{-}Bridging}$**	0.388	-0.327	0.251	-0.166	0.275	-0.422
**$^1Int_{N\text{-}AlkylatedOH2}$**	0.493	-0.293	0.246	0.053	0.298	-0.256
**$^1P_{N\text{-}Alkylation}$**	0.619	-0.314	0.262	0.087	0.322	-0.239
**$^5P_{N\text{-}Alkylation}$**	1.169	-0.329	0.267	0.068	0.191	-0.633

[a]H is the original hydrogen bonded with carbene's carbon.

Supplementary Table 21. Crystallographic data collection, processing, and refinement statistics.

	**Mb-cIII (8ESS)**	**Mb-imi (8ESU)**
**Data Collection[a]**		
Space group	P6	P6
Unit cell (Å)	a= b = 90.7, c = 45.5	a= b = 90.4, c = 45.4
	α = β = 90, γ = 120.0	α = β = 90, γ = 120.0
Wavelength (Å)	0.9795	0.9791
Resolution range (Å)	39.34 – 1.42 (1.42 – 1.40)	78.31 - 1.04 (1.06 – 1.04)
Total observations	851703 (43367)	937425 (24184)
Total unique observations	42175 (2160)	101447 (4642)
R_{merge}	0.069 (1.742)	0.067 (0.504)
R_{pim}	0.016 (0.398)	0.022 (0.234)
$\langle I/\sigma(I)\rangle$	27.2 (2.0)	18.2 (2.7)
$CC_{1/2}$	1.00 (0.714)	0.995 (0.817)
Completeness (%)	100.0 (100.0)	99.6 (92.3)
Multiplicity	20.2 (20.1)	9.2 (5.2)
**Refinement Statistics**		
Resolution range (Å)	39.34 – 1.40	78.31 – 1.04
Reflections (total)	42172	101444
Reflections (test)	2119	5080
Total atoms refined	1493	1604
R_{work}/R_{free}	0.15/0.18	0.13/0.14
RMSD bond lengths (Å)/ angles (°)	0.010/1.022	0.008/1.019
Ramachandran plot favored/allowed (%)	96.7/3.3	98.0/2.0
Average B all atoms (Å)	24.0	15.0
Molprobity all-atom clashscore	1.2	2.6

[a] Values in parentheses for data collection statistics refer to the high-resolution shell.

Supplementary Methods:

Analytical Methods:

Chiral GC Methods. Gas chromatography (GC) analysis were carried out using a Shimadzu GC-2010 gas chromatograph equipped with a FID detector, and a Cyclosil-B column (30 m x 0.25 mm x 0.25 μm film). The following GC methods were used for product analysis for **3, 4, 5, 6, 7, 8**, 1 μL injection, injector temp.: 200 ºC, detector temp: 300 ºC. Gradient for GC method: column temperature set at 140 ºC for 3 min, then to 160 ºC at 1.8 ºC/min, then to 165 ºC at 1.0 ºC/min, then to 245 ºC at 25 ºC/min, 245 ºC hold for 6 min. Total run time was 28.3 min.

**Product**	**t_R (min)**
**3**	23.34
**4**	3.51
**5**	9.19
**6**	6.86
**7**	8.30
**8**	9.44

Synthetic Procedures:

Procedure for synthesis of diazoketone 2. To generate diverse diazoketone products, two step syntheses were carried out from corresponding carboxylic acids, according to the following procedure. To a flame dried round bottom flask, carboxylic acid (1.0 mmol) was dissolved in DCM (1.0 mL) under argon with a drop of dimethylformamide as catalyst. After dropwise addition of thionyl chloride (1.5 equiv.), the reaction was stirred for 2 hours at room temperature. Solvent was removed in vacuo and used in the next step without further purification. Synthesis of diazoketone from acetyl chloride was carried out in Aldrich® diazomethane-generator with System 45™ compatible connection. CAUTION: diazomethane is an extremely sensitive explosive gas and must be handled with great caution at all times. In the outer tube of diazomethane-generator, acetyl chloride (0.3 mmol) was dissolved in Et_2O (3.0 mL) and in the inner tube, diazald (5.7 equiv.) was suspended in carbitol (1.0 mL). Once the reaction mixture was immersed in an ice bath, aqueous KOH (37%, 1.5 mL) was injected dropwise via syringe into the inner tube. After stirring for 3 hours at 0 °C, silicic acid (0.15 g) was added to the inner tube to quench any unreacted diazomethane. Solvent in the outer tube was removed and the reaction mixture was subjected to column chromatography to yield diazoketone **2** as a yellow oil (40 mg, 82% yield).

^{1}H NMR (500 MHz, $CDCl_3$): δ 7.36 (t, 2H, J = 7.1 Hz), 7.29 (d, J = 7.0 Hz, 1H), 7.24 (t, J = 7.1 Hz, 2H), 5.12 (s, 1H), 3.62 (s, 2H). Confirmed with the literature values (*J. Am. Chem. Soc.* **2021**; 143(5) *2221–2231*).

Procedure for preparative enzymatic synthesis of cyclopropane 3. To a round bottom flask, 18 mL of 22 μM myoglobin in argon-purged sodium borate buffer (50 mM, pH 9.0) and 70 mg of

sodium dithionite was added under argon. Reaction was initiated by addition of 1 mL of 400 mM styrene solution in ethanol, followed by addition 1 mL of 100 mM diazoketone solution in ethanol. The reaction was left under magnetic stirring for 16 hours at room temperature under argon. After extraction with DCM (3 x 50 mL), organic layers were combined and dried over $NaSO_4$. Solvent was removed *in vacuo* and the reaction mixture was subjected to column chromatography to yield cyclopropanes **3** as colorless oil (18 mg, 75% yield).

^{1}H NMR (500 MHz, $CDCl_3$): δ 7.34 (t, *J* = 7.3 Hz, 2H), 7.31 – 7.17 (m, 6H), 7.01 (d, *J* = 7.1 Hz, 2H), 3.88 (s, 2H), 2.52 (ddd, *J* = 9.1, 6.6, 4.0 Hz), 2.23 (ddd, *J* = 8.1, 5.2, 4.1 Hz), 1.69 (ddd, *J* = 9.2, 5.2, 4.2 Hz), 1.35 (ddd, *J* = 8.0, 6.6, 4.2 Hz). Confirmed with the literature values (*J. Am. Chem. Soc.* **2021**; 143(5) *2221–2231*).

Procedure for synthesis of 1-phenylacetone 4. Following a reported procedure (*Org. Lett.* 2019, 21, 11, 4039–4043), periodic acid (188 mg, 0.808 mmol, 1.1 eq) was added to MeCN (2 mL) while stirring at room temperature, and the suspension stirred vigorously for 15 min. Then the flask was placed in an ice-bath and 1- phenyl-2-propanol, (103 µL, 0.734 mmol, 1.0 eq) was added. To this cooled solution was added pyridinium chlorochromate (3 mg, 0.04 mmol, 0.05 eq) in MeCN (1 mL), dropwise over 5 min. The resultant creamy yellow suspension was stirred at 0 °C for 1 h and at room temperature for 1 h. Then the reaction mixture was diluted with ethyl acetate (EtOAc, 3 mL) and washed with a mixture of brine/water (1:1, 2 mL). The organic layer was then washed with a saturated solution of Na_2SO_3 (2 mL × 2) and brine (2 mL), dried over anhydrous MgSO4, filtered, and the solvent removed in vacuo to obtain 1-phenylacetone, (70 mg, 0.522 mmol, 71% yield) as a yellow oil.

^1H NMR (400 MHz, $CDCl_3$) δ 7.33 (dd, *J* = 8.1, 6.4 Hz, 2H), 7.30 – 7.24 (m, 1H), 7.20 (d, *J* = 7.0 Hz, 2H), 3.69 (s, 2H), 2.15 (s, 3H). Confirmed with the literature values (*Adv.Synth.Catal.* **2015**, 357, 1125–1130).

General procedure for synthesis of 5, 6, 7, 8. To a 10-mL round bottom flask containing a Teflon-coated magnetic stir bar was added the desired alcohols or water (0.250 mmol, 2.0 eq) and $Rh_2(OAc)_4$ (0.0125 mmol, 0.1 eq). The flask was purged with Ar (g) for 3 minutes, charged with toluene (1 mL) via gas-tight syringe, and placed in an ice-water bath with stirring under Ar (g) pressure. In a separate flame-dried 5-mL flask, BDK (**2**) (0.125 mmol, 1.0 eq) was added and diluted with toluene (1 mL). The starting material solution was added dropwise to the reaction flask. Upon complete addition of the diazo solution, the reaction mixture was stirred for an additional 5 min, then the flask was equipped with a condenser and heated to a reflux (80 °C) while stirring under Ar (g) pressure overnight. The reaction was stopped stirring, the solvent was removed via rotary evaporation and the residue was purified via flash column chromatography using silica gel and a 20% EtOAc in hexanes isocratic solvent system. The purified compounds were confirmed using ^1H NMR and ^{13}C NMR.

1-hydroxy-3-phenylpropan-2-one (5)

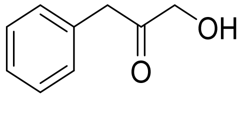

Following the procedure above, ketone (**5**) was isolated as colorless oil (3 mg, 16%). ^1H NMR (500 MHz, $CDCl_3$) δ 7.38 – 7.33 (m, 2H), 7.32 – 7.29 (m, 1H), 7.25 – 7.20 (m, 2H), 4.29 (s, 2H),

3.73 (s, 2H). ^{13}C NMR (126 MHz, $CDCl_3$) δ 133.4, 130.0, 129.7, 128.2, 68.4, 46.6. Carbonyl carbon not detected.

1-methoxy-3-phenylpropan-2-one (6)

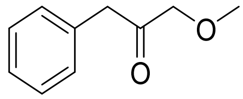

Following the procedure above, ketone (**6**) was isolated as colorless oil (5 mg, 25%). ^{1}H NMR (500 MHz, $CDCl_3$) δ 7.33 – 7.30 (m, 2H), 7.28 – 7.23 (m, 1H), 7.23 – 7.18 (m, 2H), 4.04 (s, 2H), 3.74 (s, 2H), 3.37 (s, 3H). ^{13}C NMR (126 MHz, $CDCl_3$) δ 206.6, 134.1, 130.2, 129.5, 127.9, 60.0, 46.9, 30.4.

1-ethoxy-3-phenylpropan-2-one (7)

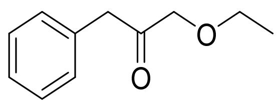

Following the procedure above, ketone (**7**) was isolated as colorless oil (4 mg, 17 %). ^{1}H NMR (500 MHz, $CDCl_3$) δ 7.31 (dd, J = 7.5, 7.5 Hz, 2H), 7.28 – 7.23 (m, 1H), 7.21 (d, J = 7.5 Hz, 2H), 4.07 (s, 2H), 3.75 (s, 2H), 3.50 (q, J = 7.0 Hz, 2H), 1.22 (t, J = 7.0 Hz, 3H). ^{13}C NMR (126 MHz, $CDCl_3$) δ 207.1, 134.3, 130.2, 129.4, 127.8, 75.9, 67.9, 46.9, 15.7.

1-isoproxy-3-phenylpropan-2-one (8)

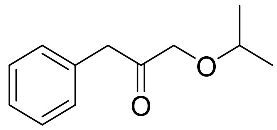

Following the procedure above, hydroxy ketone (**8**) was isolated as colorless oil (2 mg, 7%). ^{1}H NMR (500 MHz, $CDCl_3$) δ 7.30 (dd, *J* = 7.5, 7.3 Hz, 2H), 7.28 – 7.23 (m, 1H), 7.21 (d, *J* = 7.5 Hz, 2H), 4.05 (s, 2H), 3.78 (s, 2H), 3.57 (p, *J* = 6.1 Hz, 1H), 1.16 (d, *J* = 6.1 Hz, 6H). ^{13}C NMR (126 MHz, $CDCl_3$) δ 130.3, 129.4, 127.7, 73.7, 73.4, 46.9, 22.6. Quaternary carbon not detected.

Synthesis of *cis*-β-deuterstyrene (*cis-d*-1)

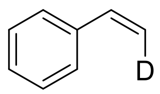

The cis-β-deutero-styrene was prepared in two steps from phenylacetylene following the literature procedure (J. Am. Chem. Soc. 2018, 140, 1649−1662) in (200 mg, 45% yields over two steps) as slightly yellow oil.

^{1}H NMR (500 MHz, $CDCl_3$) δ 7.38 (d, *J* = 7.5 Hz, 2H), 7.30 (t, *J* = 7.5 Hz, 2H), 7.27 – 7.19 (m, 1H), 6.75 – 6.62 (m, 1H), 5.20 (d, *J* = 10.9 Hz, 1H). Confirmed with the literature value (*J. Am. Chem. Soc.* ***2018****; 140 (5) 1649–1662*).

2-phenyl-1-((1S,2S,3R)-2-phenylcyclopropyl-3-deutero)ethan-1-one (*d*-3)

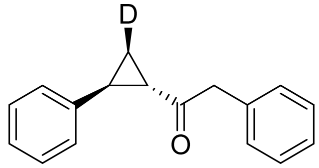

Four different procedures with four different catalysts were used to generate deuterated cyclopropane ***d*-3**. Mb-catalyzed reactions were carried out with 11 mM *cis*-β-deuterostyrene (***cis-d*-1**), 10 mM BDK (**2**), 10mM $Na_2S_2O_4$ and 20 μM Mb(H64G,v68A) in sodium borate buffer (50

mM, pH 9) with 10% ethanol. Fe(TPP)Cl-catalyzed reactions were carried out with 11 mM *cis*-β-deuterostyrene (***cis*-*d*-1**), 10 mM BDK (**2**), 20 mM $Na_2S_2O_4$ and 0.5 mM Fe(TPP)Cl in toluene with 10% water. Co(TPP) -catalyzed reactions were carried out with 11 mM *cis*-β-deuterostyrene (***cis*-*d*-1**), 10 mM BDK (**2**) and 0.2 mM Co(TPP)Cl in DCM. Hemin-catalyzed reactions were carried out with 11 mM *cis*-β-deuterostyrene (***cis*-*d*-1**), 10 mM BDK (**2**), 10 mM $Na_2S_2O_4$ and 60 μM Hemin in sodium borate buffer (50 mM, pH 9) with 10% ethanol. In a typical reaction, 18 mL of the catalyst in argon-purged buffer or solvent was added to the round bottom flask and appropriate amount of sodium dithionite dissolved in water was added under argon. Reaction was initiated by addition of 1 mL of 220 mM styrene solution in ethanol, followed by addition 1 mL of 200 mM diazoketone solution in ethanol. The reaction product was extracted with dichloromethane (20 mL, three times), and the organic layers were collected and dried over anhydrous sodium sulfate. The organic solvent was removed by rotary evaporation and the reaction mixture was purified via flash column chromatography using silica gel and 5% EtOAc/hexanes as the eluent to afford the desired product as a clear, colorless oil. Yields for each catalytic reaction are listed on **Supplementary Table 4**.

^{1}H NMR (500 MHz, $CDCl_3$) δ 7.28 (d, *J* = 7.3 Hz, 2H), 7.26 – 7.11 (m, 6H), 6.96 (d, *J* = 7.5 Hz, 2H), 3.83 (s, 2H), 2.46 (dd, *J* = 9.0, 4.5 Hz, 1H), 2.17 (dd, *J* = 5.2, 4.5 Hz, 1H), 1.63 (dd, *J* = 9.0, 5.2 Hz, 1H). ^{2}H NMR (61 MHz, D_2O) δ 1.35 (s, 1H).

Computational Methods

All calculations were performed using the program Gaussian 16.[21] All models investigated in this work were subject to full geometry optimizations without any symmetry constraints using the PCM method[22] with a typical dielectric constant of 4.0 applied to simulate the protein environment effect as done previously.[23] The frequency analysis was used to verify the nature of the stationary points on respective potential energy surfaces and to provide zero-point energy corrected electronic energies (E_{ZPE}'s), enthalpies (H's), and Gibbs free energies (G's) at 1 atm and experimental reaction temperatures, i.e. room temperature (RT). The atomic charges and spin densities reported here are from the Natural Population Analysis (NPA) and Mulliken schemes respectively, as implemented in Gaussian 16. The relative spin state energies, absolute values of electronic energies (E's), zero-point energy corrected electronic energies (E_{ZPE}'s), enthalpies (H's), Gibbs free energies (G's), key geometric parameters, charges, spin densities, 3D structures and coordinates of optimized structures are in **Supplementary Tables 5-9** and its subsequent section.

All calculations were done using a range-separated hybrid DFT method with dispersion correction, ωB97XD,[24] based on its excellent performance on heme carbenes and other catalytic systems (among several DFT functionals considered) from previous methodological studies.[4,25-27] This ωB97XD method was found to yield accurate predictions of various experimental spectroscopic properties, structural features, and reactivity results of heme carbenes.[4,26-31] Calculations were performed with the heme core part [Fe(Por)(5-MeIm] as the model for the myoglobin biocatalyst (called $\mathbf{R_{heme}}$) with diazoketone [$CHCOCH_2PhN_2$] (called $\mathbf{R_2}$), where Por is non-substituted porphyrin and 5-MeIm is 5-methylimidazole to represent axial His ligand, which are the same as used in recent heme carbene reaction studies.[26,28,30,31] The basis set includes the effective core potential (ECP) basis LanL2DZ[32] for iron and the triple-zeta basis 6-311G(d) for all

other elements, which was found to provide accurate predictions of various experimental reaction properties of heme carbenes.[26-31] The use of a much larger 6-311++G(2d,2p) basis for all non-metal atoms was found to yield similar results for heme carbene reactions[27] and thus further support the efficient use of the current basis set here. The use of an ECP basis for metal is common in many reaction studies involving transition metal carbenoids, such as Ir porphyrin carbene[33], Ru porphyrin carbene,[34] and Rh carbene.[35] The advantage of an ECP basis is the inclusion of relativistic effect basically absent in an all-electron basis set. In addition, it is available for all transition metals, which may allow direct comparisons of effects of a vast amount of metal centers. The alternative use of an all-electron basis for the metal center[28] was recently found to yield qualitatively same conclusions of geometric, electronic, and energetic features for heme carbene reactions, and therefore supports the use of LanL2DZ basis here.

The chirality of the product and corresponding transition state was based on the experimental results as described in the main text, i.e. *S, S* chirality of C and C_2, respectively, for the cyclopropanation product. The absolute and relative energies are shown in Tables S6 and S7 respectively. The selected geometric and charge data are listed in Tables S8 and S9 respectively. The 3D structures and coordinates of these optimized species are shown further below.

For $^{1}\mathbf{Int_{IPC}}$ conformations, the benzene ring of diazoketone can be either perpendicular or parallel to the porphyrin plane, see **Supplementary Figure 11**, with the former conformation being slightly more favorable by ΔG of 0.99 kcal/mol. Therefore, this conformation was used for this intermediate and related transition state calculations.

The ^{57}Fe quadrupole splitting arises from the non-spherical nuclear charge distribution in the I*=3/2 excited state in the presence of an electric field gradient at the ^{57}Fe nucleus, while the isomer shift arises from differences in the electron density at the nucleus between the absorber (the

molecule or system of interest) and a reference compound (usually α-Fe at 300K). The former effect is related to the components of the electric field gradient (EFG) tensor at the nucleus as follows:[6]

$$\Delta E_Q = \frac{1}{2} eQV_{zz}\left(1+\frac{\eta^2}{3}\right)^{1/2} \qquad (1)$$

where *e* is the electron charge, *Q* is the quadrupole moment of the E*=14.4 keV excited state, and the principal components of the EFG tensor are labeled according to the convention:

$$|V_{zz}|>|V_{yy}|>|V_{xx}| \qquad (2)$$

with the asymmetry parameter being given by:

$$\eta = \frac{V_{xx}-V_{yy}}{V_{zz}} \qquad (3)$$

The isomer shift in ^{57}Fe Mössbauer spectroscopy is given by:[7]

$$\delta_{\mathrm{Fe}} = E_A - E_{\mathrm{Fe}} = \frac{2\pi}{3}\mathrm{Z}e^2(<R^2>* - <R^2>)(|\psi(0)|_A^2 - |\psi(0)|_{\mathrm{Fe}}^2) \qquad (4)$$

where Z represents the atomic number of the nucleus of interest (iron) and R, R* are average nuclear radii of the ground and excited states of ^{57}Fe. Since $|\psi(0)|_{\mathrm{Fe}}^2$ is a constant, the isomer shift (from Fe) can be written as:

$$\delta_{\mathrm{Fe}} = \alpha\,[\rho(0) - c] \qquad (5)$$

where α is the so-called calibration constant and ρ(0) is the computed charge density at the iron nucleus. Both α and c can be obtained from the correlation between experimental δ_{Fe} values and the corresponding computed ρ(0) data in a training set. Then, one can use equation (5) to predict δ_{Fe} for a new molecule from its computed ρ(0), basically as described in detail elsewhere for a wide variety of heme and other model systems.[36] The hybrid functional B3LYP[37] with a Wachter's basis for Fe,[38] 6-311G* for all the other heavy atoms and 6-31G* for hydrogens was used to predict

Mössbauer quadrupole splittings and isomer shifts, the same approach used in the previous work for >50 iron-containing proteins and models with experiment-versus-theory linear correlation coefficients R^2=0.98 and 0.97, respectively.[3-17] These systems cover a broad range of iron systems, including all iron spin states and all coordination states. To calculate ΔE_Q, we first evaluated the principal components of the electric field gradient tensor at the ^{57}Fe nucleus (V_{ii}), then we used equation (1) to deduce ΔE_Q, using a precise recent determination of Q = 0.16 (±5%) x 10^{-28}m^2,[39] a value previously found to permit excellent accord between theory and experiment in a broad range of systems.[3-17] To calculate δ_{Fe} values, we read the Kohn-Sham orbitals from the *Gaussian 16*[21] results into the AIM 2000 program,[40] to evaluate the charge density at the iron nucleus, ρ(0). Then, we evaluated the isomer shifts by using the equation derived previously:[7]

$$\delta_{Fe} = -0.404\ [\rho(0) - 11614.16] \qquad (6)$$

A tighter statistical evaluation of different models was also performed using the reduced χ^2 analysis and the Z-surface technique used before for protein structure refinement and determination.[8,17,18] The reduced χ^2 is calculated using the following formula:

$$\chi^2 = 1/2\ [(\delta_{Fe}^{calc} - \delta_{Fe}^{expt})^2/SD(\delta_{Fe})^2 + (\Delta E_Q^{calc} - \Delta E_Q^{expt})^2/SD(\Delta E_Q)^2]$$

where SD are standard deviations of such property calculations obtained previously for model systems.[3-17] The Z-surface involves the calculation of conditional probabilities. First, each Z_1 value is calculated using the following expression:[8]

$$Z_1\ (\delta_{Fe}) = \exp\ [-(\delta_{Fe}^{calc} - \delta_{Fe}^{expt})^2/W(\delta_{Fe})^2]$$

$$Z_1\ (\Delta E_Q) = \exp\ [-(\Delta E_Q^{calc} - \Delta E_Q^{expt})^2/W(\Delta E_Q)^2]$$

where W is a search width parameter. Then, the overall probability is computed as follows,

$$Z = [Z_1(\delta_{Fe}) \times Z_1(\Delta E_Q)]^{1/2}$$

The W parameters were chosen to yield Z values in the ~0.1-0.9 range over the entire Z surfaces in each case, to not over-emphasize the contributions of any given property to the final result. The used parameters are 0.07 mm/s for $W(\delta_{Fe})$ and 0.3 mm/s for $W(\Delta E_Q)$.[17]

3D structures and Cartesian coordinates of the optimized species in the studied reaction pathways

$^{5}R_{heme}$

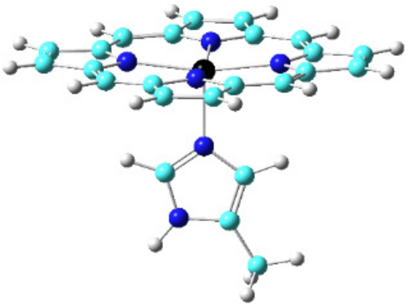

N	-1.80773000	-1.21219200	-0.21888600
N	-1.41834900	1.66747400	-0.54520700
N	1.46271100	1.28839700	-0.84925400
N	1.05607700	-1.60181400	-0.65941100
C	-1.77960700	-2.56998000	-0.17275700
C	-3.10391200	-0.82016800	-0.10801000
C	0.67449800	-2.90256600	-0.53013100
C	2.40263700	-1.58164200	-0.86022800
C	2.74693900	0.88996200	-1.04303300
C	1.42328100	2.63947500	-0.98294400
C	-1.04044400	2.96661100	-0.69967900
C	-2.76832900	1.64615600	-0.36422900
C	0.26811400	3.41567000	-0.89666100
H	0.39475500	4.48762600	-1.01359000
C	-3.54402600	0.50253300	-0.15761700
H	-4.61185300	0.66189200	-0.04257300
C	-0.62954400	-3.34883400	-0.29993900
H	-0.76326200	-4.42428900	-0.23310400
C	3.18267000	-0.43457900	-1.03029400
H	4.24422900	-0.59628200	-1.19078600
C	2.75917900	3.13353200	-1.25294000
C	3.57711100	2.05261100	-1.28971300
C	2.90079000	-2.93684100	-0.87120400
C	1.83354100	-3.75253500	-0.66765300
C	-3.12833000	-3.07077100	0.00217500
C	-3.94662000	-1.98959300	0.04224400
C	-3.27522600	2.99704800	-0.41869900
C	-2.20836900	3.81222100	-0.62590200
N	0.16015000	0.17064300	1.83673800
C	1.16870400	0.82191400	2.50787600
C	-0.58441300	-0.40171100	2.75358400
H	-1.46357500	-0.99750700	2.56356400
C	1.02975900	0.63930300	3.85270700
N	-0.09752100	-0.14511100	3.98505200

H	1.92480100	1.37628800	1.97308400
C	1.82992100	1.11378000	5.01494800
H	2.67461900	1.70694500	4.66380000
H	1.23433700	1.74027000	5.68482900
H	2.22546300	0.27797100	5.59882100
H	-0.49188500	-0.47203600	4.85137300
Fe	-0.14000100	0.05435100	-0.30587700
H	-2.20473000	4.88968900	-0.72245700
H	-4.31474200	3.27747800	-0.31352300
H	-5.02323600	-1.97434100	0.14757900
H	-3.40307700	-4.11503300	0.06894700
H	1.82378300	-4.83306800	-0.61634800
H	3.93439900	-3.21982600	-1.01900000
H	4.64196900	2.03067400	-1.47934000
H	3.02170200	4.17170400	-1.40659400

R_2

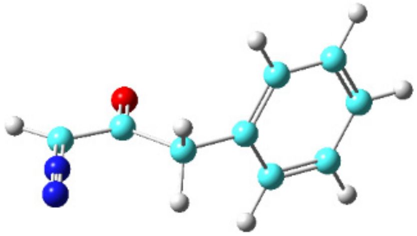

C	-0.63134200	0.28042800	3.10720600
C	0.72905300	0.53346000	3.56388500
O	1.10880900	0.07856400	4.62224300
N	-1.05957500	0.75864800	1.97008100
N	-1.41048000	1.17787600	0.98861400
H	-1.33820200	-0.30471100	3.67679500
C	1.61868000	1.38433000	2.65536900
C	3.01057600	1.56643800	3.19378700
C	3.30418800	2.62079700	4.05601900
C	4.02374800	0.67094400	2.85847200
C	4.58484400	2.78008000	4.57129900
H	2.52233900	3.32382100	4.32860000
C	5.30618000	0.82611100	3.37125700
H	3.80751400	-0.15776000	2.19036000
C	5.59032700	1.88253600	4.22931800
H	4.79815400	3.60689400	5.24075900
H	6.08476800	0.12091600	3.09953500
H	6.59104800	2.00594900	4.62978300
H	1.64947300	0.91758900	1.66451600
H	1.13286100	2.35660800	2.51434300

^{1}Int$_{C1}$

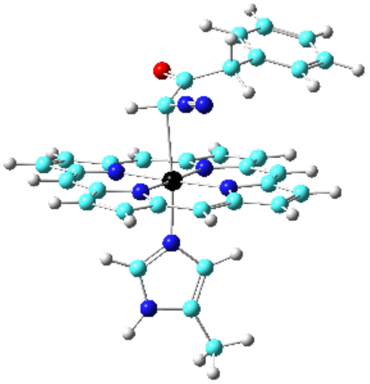

N	-1.82903400	-1.14680900	-0.21048800
N	-1.45852900	1.65010300	-0.59817600
N	1.26204400	1.19976500	-1.28739200
N	0.89422200	-1.60088000	-0.90619600
C	-1.83974300	-2.50838200	-0.14023300
C	-3.06053300	-0.73427000	0.20321800
C	0.51900200	-2.90221500	-0.75496100
C	2.22275500	-1.61886200	-1.19704800
C	2.54504900	0.80354300	-1.51387700
C	1.25042400	2.55233400	-1.44621900
C	-1.11291300	2.94185000	-0.86064900
C	-2.74065100	1.68705700	-0.13226200
C	0.14443300	3.36761200	-1.26651600
H	0.28086400	4.43151400	-1.43026000
C	-3.48337300	0.58569500	0.26267200
H	-4.49391600	0.76833900	0.61341700
C	-0.75671900	-3.33234500	-0.41705500
H	-0.91203900	-4.40270000	-0.32648800
C	3.00162600	-0.50206700	-1.46025100
H	4.05185300	-0.66950800	-1.67577200
C	2.56644900	3.02827000	-1.80302200
C	3.37118900	1.94332100	-1.84049300
C	2.71044600	-2.97727900	-1.22612200
C	1.65028200	-3.77599400	-0.96021400
C	-3.12613200	-2.97479100	0.31728900
C	-3.88082900	-1.87195800	0.54059800
C	-3.22964000	3.04446400	-0.12372300
C	-2.22232200	3.82315400	-0.58457000
N	0.25015600	0.22739100	1.19347500
C	0.59297300	1.38515900	1.85081200
C	0.33935800	-0.74254200	2.07235900
H	0.13709300	-1.78406800	1.88096500
C	0.89516500	1.10793800	3.15142400
N	0.72594500	-0.25483800	3.26998700

H	0.60028600	2.33658000	1.34341800
C	1.32473100	1.97628300	4.28159400
H	1.38112500	3.01307700	3.94886900
H	0.62018600	1.92940100	5.11684800
H	2.31137800	1.69017700	4.65703400
H	0.86651400	-0.79914100	4.10416600
Fe	-0.27269300	0.02802400	-0.72202400
H	-2.20926100	4.89666600	-0.71729200
H	-4.21870100	3.34464700	0.19512200
H	-4.90180400	-1.81742700	0.89396000
H	-3.39682300	-4.01321100	0.45386000
H	1.61945900	-4.85567200	-0.90111000
H	3.73197200	-3.26451000	-1.43583700
H	4.42485600	1.89627200	-2.07948700
H	2.81524200	4.05906600	-2.01340000
C	-0.89402700	-0.44812900	-3.01686000
C	0.09905500	0.19980600	-3.89673900
N	-2.14035000	0.00807400	-3.04331700
O	1.10834400	-0.39994300	-4.18750000
C	-0.25808500	1.58641800	-4.41293700
N	-3.16979500	0.43395700	-3.02612300
C	0.86484700	2.49469700	-4.85439000
H	-0.94287400	1.41766600	-5.25697700
H	-0.84847300	2.10506100	-3.65246400
C	0.74669200	3.86074600	-4.59456000
C	1.98849700	2.04567300	-5.54804200
C	1.72922000	4.75661100	-4.99398100
H	-0.12327900	4.22662700	-4.05748700
C	2.97883600	2.94026000	-5.94045700
H	2.10119400	0.99178800	-5.76078300
C	2.85700700	4.29654700	-5.66422600
H	1.61580300	5.81387800	-4.77616800
H	3.85186500	2.57044600	-6.46869700
H	3.63206000	4.99054600	-5.97291000
H	-0.83312100	-1.51367900	-2.83755200

^{1}TS$_{Carbene}$

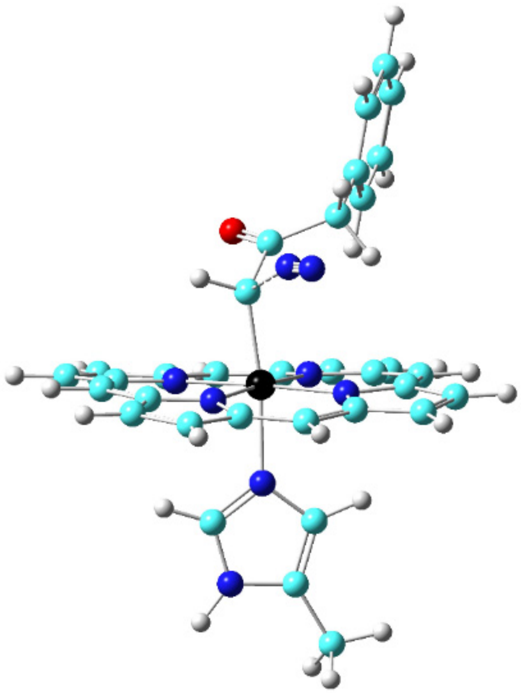

N	-1.50184000	-0.40723200	-0.39373000
N	-0.11975000	2.07616800	-0.46353700
N	2.37329100	0.67400000	-0.62403000
N	0.98407400	-1.80833900	-0.53609200
C	-1.97850700	-1.67126400	-0.22238200
C	-2.58761700	0.41581000	-0.37977000
C	0.17029000	-2.88195800	-0.34453500
C	2.24354200	-2.30141800	-0.68505300
C	3.44752400	-0.14735400	-0.77550000
C	2.86227400	1.94097300	-0.54759000
C	0.71569300	3.14347600	-0.34684900
C	-1.38495300	2.56923900	-0.37563500
C	2.10100800	3.08860500	-0.39274500
H	2.63557100	4.03009600	-0.31942200
C	-2.54317300	1.80334600	-0.39122900
H	-3.48980900	2.33168600	-0.34223000
C	-1.20601100	-2.82396300	-0.17856000
H	-1.72849500	-3.76445300	-0.03616900
C	3.39252900	-1.53370200	-0.82984100
H	4.33256900	-2.06377100	-0.94467300
C	4.30300600	1.92385200	-0.65426600
C	4.66604600	0.62909800	-0.80627600
C	2.22704900	-3.74392500	-0.60191900
C	0.94076600	-4.10355200	-0.37856600
C	-3.41915600	-1.65248400	-0.12194300

C	-3.79850800	-0.35761500	-0.23173900
C	-1.35327000	4.00288300	-0.20649800
C	-0.04663100	4.35860700	-0.18079600
N	0.54695000	0.14750900	1.49650400
C	-0.37915200	0.62596700	2.39279300
C	1.54556200	-0.31362900	2.20999700
H	2.44387800	-0.76190400	1.81657900
C	0.06896400	0.45035600	3.66900300
N	1.30087600	-0.15203400	3.52843400
H	-1.30669600	1.06434000	2.06011200
C	-0.52869400	0.78261300	4.99131900
H	-1.50326600	1.25029600	4.84826700
H	-0.67229200	-0.11108800	5.60544100
H	0.09854700	1.47983200	5.55427600
H	1.91684700	-0.42423100	4.27566900
Fe	0.43285300	0.13151600	-0.55535100
H	0.37839900	5.34677700	-0.06762000
H	-2.22499000	4.63722900	-0.11942300
H	-4.79775200	0.05524400	-0.19729900
H	-4.04270600	-2.52566500	0.01531400
H	0.53389500	-5.09785300	-0.25186500
H	3.09684600	-4.38095200	-0.69124800
H	5.66045900	0.21878800	-0.91994500
H	4.93679600	2.79975100	-0.62276600
C	0.57712100	0.13859100	-2.50746900
C	0.33036300	1.35378100	-3.33698300
N	-0.63752200	-1.08755100	-3.16792000
O	1.28270800	2.08183200	-3.50550600
C	-1.07518300	1.67983000	-3.81410300
N	-1.33611900	-1.92658100	-3.22965700
C	-1.19957400	3.00641700	-4.51028100
H	-1.40600800	0.87844600	-4.48223800
H	-1.72979900	1.62561500	-2.93651100
C	-1.52555900	3.07298200	-5.86260900
C	-0.99466800	4.19483700	-3.80982900
C	-1.65248100	4.30077400	-6.50504300
H	-1.68391300	2.15592100	-6.42338100
C	-1.12052900	5.42166400	-4.44692500
H	-0.73111000	4.15754700	-2.75883300
C	-1.45133000	5.47933700	-5.79769400
H	-1.90889000	4.33415400	-7.55897000
H	-0.95745000	6.33699100	-3.88697700
H	-1.54971600	6.43840900	-6.29567800
H	1.42173200	-0.43575100	-2.89754400

^{1}Int$_{IPC}$

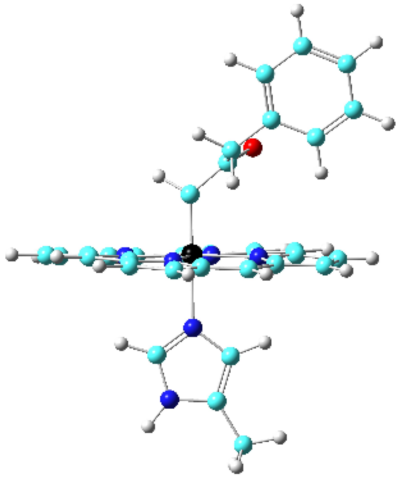

C	0.31280500	-0.09361800	-1.68390600
C	-0.65432800	-0.43281500	-2.74582900
O	-1.54317500	0.34282700	-3.02355700
H	1.13840900	0.53225700	-2.05010400
Fe	0.21120200	-0.50786800	0.02081300
N	1.98265400	0.40162900	0.33545900
N	1.15621100	-2.27274000	-0.17519800
N	-0.75071700	1.20604000	0.43156900
N	-1.56929600	-1.45117400	-0.18217700
C	2.17991300	1.70628600	0.66778500
C	3.21890700	-0.15441600	0.21131600
C	2.50280200	-2.47209800	-0.21423900
C	0.58120800	-3.49840900	-0.32046800
C	-2.09237900	1.42185800	0.36660500
C	-0.18244700	2.39810000	0.75752900
C	-1.77687500	-2.79170600	-0.30180500
C	-2.80368000	-0.87717800	-0.14589000
C	1.17623000	2.63865300	0.88026400
C	3.59196100	1.98871500	0.75967400
C	4.23806200	0.83426600	0.46937200
C	3.46932200	-1.49112300	-0.06081300
C	2.79170400	-3.87430200	-0.39788400
C	1.59988500	-4.51185100	-0.45551600
C	-0.78025100	-3.75289000	-0.36353300
C	-3.05274500	0.46637400	0.08066300
C	-2.38773300	2.80110000	0.67419700
C	-1.20386100	3.40532400	0.92315800
C	-3.18894000	-3.07562800	-0.36429100
C	-3.82737000	-1.88369600	-0.27817300
H	1.48021000	3.64681100	1.14176500
H	4.01634400	2.95095200	1.01227300

H	5.30333800	0.64982500	0.43710800
H	4.50710300	-1.80201600	-0.12073100
H	3.78490200	-4.29748300	-0.46229500
H	1.40696800	-5.56854400	-0.58093100
H	-1.09010800	-4.78660800	-0.47404600
H	-4.08821300	0.78937500	0.07315500
H	-3.38002400	3.23048100	0.69291900
H	-1.01692400	4.43703100	1.18807000
H	-3.61818300	-4.06309900	-0.46542100
H	-4.89084000	-1.68783400	-0.28749200
N	0.11243100	-0.92678300	2.10670700
C	1.12087900	-0.91426900	2.94461100
C	-0.99633600	-1.26448800	2.84635100
H	2.14453900	-0.68813100	2.69341600
N	0.70797500	-1.23038900	4.19112100
C	-0.65049000	-1.45811400	4.15168400
H	-1.97121800	-1.34422300	2.39232600
H	1.29489200	-1.28638600	5.00631400
C	-1.44932900	-1.82950500	5.35174700
H	-2.49517600	-1.96040800	5.07238600
H	-1.10035500	-2.76678000	5.79457700
H	-1.40239700	-1.05535900	6.12299800
C	-0.43728400	-1.77225000	-3.43126400
C	-1.48548800	-2.10963800	-4.45347300
C	-1.19594000	-2.10469100	-5.81516900
C	-2.77949400	-2.42943400	-4.04139200
C	-2.17577200	-2.42144700	-6.75103100
H	-0.19379900	-1.85241400	-6.15013800
C	-3.75988600	-2.74498900	-4.97215800
H	-3.01850700	-2.42534100	-2.98213800
C	-3.46048800	-2.74368600	-6.33155800
H	-1.93323700	-2.41605100	-7.80878100
H	-4.76212100	-2.99115000	-4.63623200
H	-4.22624800	-2.99093900	-7.05952200
H	0.56386700	-1.75013600	-3.87813500
H	-0.38871500	-2.53399200	-2.64751900

N_2

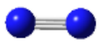

N	-1.10516000	0.42671500	4.30570300
N	-1.63644400	1.03842500	5.03790400

Styrene

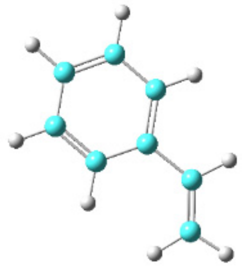

C	0.82071900	-0.14163400	-0.02315400
C	2.16642900	0.21117700	0.03589200
C	2.54034400	1.54639900	0.06972900
C	1.57687500	2.56164100	0.04104400
C	0.22964700	2.19165200	-0.00928300
C	-0.14740200	0.85443100	-0.04404300
H	0.53108100	-1.18678800	-0.04765000
H	2.92876900	-0.56055200	0.06102000
H	3.59404900	1.79771800	0.13014300
H	-0.53364000	2.96436700	-0.02522700
H	-1.19927100	0.59177600	-0.08586100
C	1.92313000	3.99524600	0.06408400
C	3.13373600	4.52965200	-0.08139000
H	1.07842700	4.66748900	0.20472600
H	4.02583500	3.93274000	-0.24467500
H	3.27330300	5.60448600	-0.04817400

$^{1}TS_{CP}$

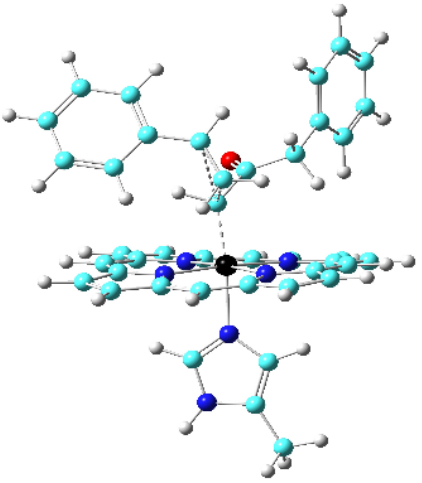

C	-0.05359500	-0.04564100	-1.88496700

C	-1.19552000	-0.45582600	-2.75613900
O	-2.10457700	0.33954700	-2.87681400
H	0.26586400	0.96280300	-2.15468100
Fe	0.10283000	-0.44059000	-0.03996000
N	1.71735700	0.76392800	0.12790900
N	1.29396000	-2.05558200	-0.04905600
N	-1.09600300	1.17804800	0.11524400
N	-1.51620000	-1.65089800	-0.04178400
C	1.72586100	2.12275900	0.15770400
C	3.01051900	0.36795900	0.28218300
C	2.64692700	-2.06723300	0.09948800
C	0.91396900	-3.35474000	-0.19505500
C	-2.45363900	1.17289500	0.19785800
C	-0.71301400	2.48471500	0.10446700
C	-1.52566600	-3.00885100	-0.12091800
C	-2.81130200	-1.26714000	0.11752900
C	0.59864600	2.93337700	0.10205200
C	3.07490600	2.61089800	0.32143200
C	3.87318700	1.51979000	0.40839400
C	3.44903000	-0.94877100	0.27864200
C	3.14652100	-3.41989400	0.01833900
C	2.07154500	-4.21886200	-0.17642300
C	-0.39691100	-3.80722600	-0.24628600
C	-3.25420800	0.04295300	0.22641900
C	-2.95228300	2.52816400	0.24305800
C	-1.87430800	3.34186300	0.17437300
C	-2.87717500	-3.50662000	-0.02386800
C	-3.67548900	-2.42395600	0.13455300
H	0.75711500	4.00674400	0.11085200
H	3.35511200	3.65442800	0.37296700
H	4.94597700	1.48041900	0.54120300
H	4.51452100	-1.11736000	0.39675800
H	4.18726700	-3.70225000	0.10267800
H	2.04339500	-5.29535500	-0.27864100
H	-0.55089800	-4.87806100	-0.33107000
H	-4.32440500	0.19730500	0.31624600
H	-3.99676700	2.80059600	0.30979500
H	-1.84625300	4.42308000	0.17810900
H	-3.15956700	-4.54983300	-0.06581100
H	-4.75076300	-2.39276200	0.24783200
N	0.06654100	-0.56735600	2.03528200
C	0.15761500	0.44026300	2.86871700
C	-0.05174400	-1.69777800	2.80829600
H	0.26206200	1.47703600	2.59113300
N	0.10286300	0.00580700	4.14654800
C	-0.03187900	-1.36613100	4.13124800

H	-0.14318500	-2.67499800	2.36101200
H	0.14925700	0.58773500	4.96570000
C	-0.12363700	-2.18888200	5.36857100
H	-0.22077400	-3.24235200	5.10431000
H	0.76813800	-2.08045600	5.99267600
H	-0.99245800	-1.91342000	5.97329100
C	-1.26268600	-1.85646800	-3.34954500
C	-2.48143900	-2.08955500	-4.19690400
C	-2.39938300	-2.04717000	-5.58689400
C	-3.72035400	-2.33384800	-3.60603700
C	-3.52827600	-2.24834300	-6.37429600
H	-1.44055300	-1.85560900	-6.06164100
C	-4.85024600	-2.53621700	-4.38766800
H	-3.79477300	-2.36404900	-2.52353500
C	-4.75798200	-2.49445600	-5.77560600
H	-3.44591600	-2.21372100	-7.45587800
H	-5.80732400	-2.72646500	-3.91243700
H	-5.64055700	-2.65294600	-6.38672200
H	-0.35957000	-2.04969700	-3.93092100
H	-1.22053400	-2.55467600	-2.50908100
C	1.86162000	-0.91941200	-2.89950800
C	1.59353700	-0.12793600	-3.95587900
H	1.58769100	-1.96701800	-2.89008300
C	1.95840000	1.28439000	-4.09715400
H	1.01169800	-0.53425000	-4.78087900
C	2.75131600	1.95298900	-3.15647100
C	1.45760000	2.00661100	-5.18533700
C	3.02919700	3.30229800	-3.30181400
H	3.15059100	1.42360500	-2.29904000
C	1.73969600	3.35815400	-5.33317300
H	0.83166000	1.50396100	-5.91669700
C	2.52569600	4.01010000	-4.39013400
H	3.63883900	3.80332900	-2.55741700
H	1.33976000	3.90360000	-6.18107800
H	2.74351900	5.06713800	-4.50028100
H	2.48426800	-0.59180300	-2.08057500

P_{CP}

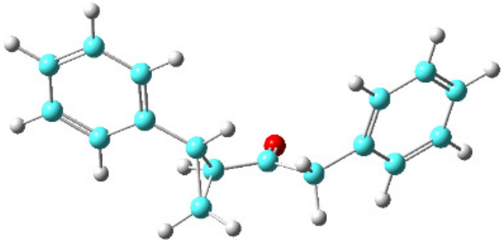

C	2.88185400	-8.08653100	-2.11413700
C	1.80491200	-7.90348800	-3.12451700
O	0.96522600	-8.76083700	-3.27798700
H	2.87711300	-9.08326400	-1.68699600
C	4.21163800	-7.37767000	-2.19095400
H	5.08352500	-7.94570100	-1.88788600
H	4.38644100	-6.69602700	-3.01508700
C	3.23620900	-6.96347300	-1.13583600
H	2.72835000	-6.02091000	-1.31280200
H	4.78477400	-9.56980300	2.41813400
C	4.26851200	-8.67009600	2.10001400
C	2.94973100	-6.38054400	1.26639500
C	3.77999300	-7.77518600	3.04641300
C	4.09912500	-8.41964500	0.74489500
C	3.43673000	-7.26987800	0.30799500
C	3.11940800	-6.62848100	2.62372000
H	3.91314100	-7.97210100	4.10474400
H	4.48799600	-9.13566900	0.02682000
H	2.73236800	-5.92280000	3.35134900
H	2.43042600	-5.48174600	0.94701400
C	0.48896400	-6.29513900	-4.58437100
C	0.01212300	-6.97881700	-5.70186300
C	-0.30435300	-5.29932200	-4.01845000
C	-1.22888300	-6.67109400	-6.24449000
H	0.61762800	-7.76116000	-6.14841700
C	-1.54780200	-4.98837600	-4.55799900
H	0.05303700	-4.75896500	-3.14616900
C	-2.01322100	-5.67395300	-5.67368000
H	-1.58556800	-7.21163000	-7.11511700
H	-2.15216600	-4.20862000	-4.10618600
H	-2.98234300	-5.43251400	-6.09748900
C	1.82417700	-6.63924600	-3.98356200
H	2.21395400	-5.79208400	-3.41354900
H	2.56339000	-6.82775400	-4.77235300

TS$_{non}$

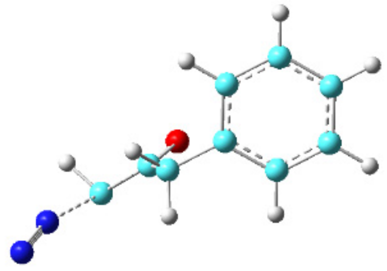

C	-0.26378880	-0.59504866	0.22934572
N	-0.03659978	1.16525651	1.44842943
C	1.06259925	-1.07839901	0.02890652
N	-0.20162452	1.82506417	2.30322799
O	0.99426667	-1.98430589	-0.79902961
H	-0.62600454	0.02824176	-0.59341342
C	2.30644229	-0.69995311	0.80135808
H	2.06411183	-0.76414074	1.86754061
H	2.52017781	0.35555386	0.60489173
C	3.49168186	-1.56210020	0.45997175
C	4.45112807	-1.12514389	-0.44997445
C	3.63214386	-2.82528715	1.03260403
C	5.53587458	-1.93095404	-0.77787492
H	4.35095373	-0.14496625	-0.90724939
C	4.71379407	-3.63345740	0.70764559
H	2.88636726	-3.17958506	1.73812590
C	5.66990461	-3.18723765	-0.19894017
H	6.27683566	-1.57597893	-1.48647125
H	4.81051351	-4.61385467	1.16212632
H	6.51584070	-3.81718385	-0.45291107

Carbene

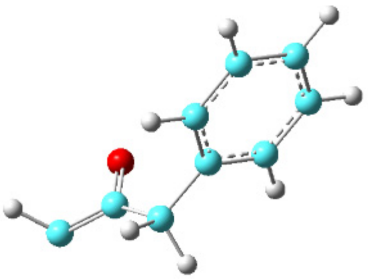

C	-0.26198430	-0.58466269	0.05639492
C	1.04380143	-1.03026537	0.16093597

O	0.77765058	-2.10588581	-0.41773357
H	-0.48038521	-0.09415743	-0.89693733
C	2.31922560	-0.61906498	0.81281925
H	2.12466682	-0.62833617	1.89164466
H	2.51173071	0.42650591	0.55557282
C	3.48286017	-1.51009791	0.46022044
C	4.46153604	-1.07897826	-0.43182311
C	3.58261837	-2.78759295	1.00997586
C	5.52746756	-1.90718840	-0.76568833
H	4.39341695	-0.08692658	-0.86858333
C	4.64499300	-3.61750488	0.67642250
H	2.82339624	-3.13469033	1.70406814
C	5.62098115	-3.17844118	-0.21225646
H	6.28510769	-1.55751083	-1.45890831
H	4.71142266	-4.60899100	1.11149660
H	6.45204654	-3.82565313	-0.47148872

^{1}Int$_{N1}$

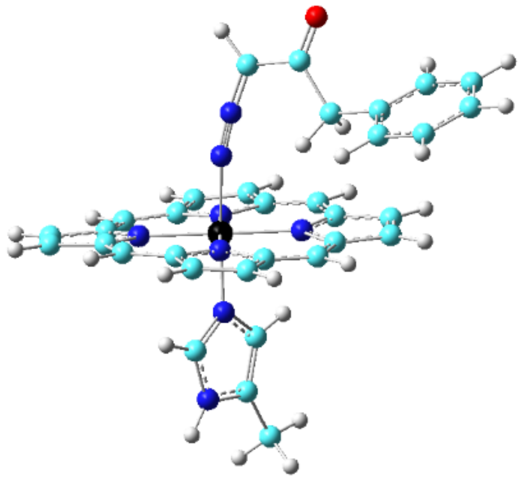

N	-1.78788700	-1.19225500	-0.10034500
N	-1.12001100	1.40731500	-1.05366900
N	1.61252200	0.62139100	-1.28469600
N	0.94343300	-1.98452100	-0.34750100
C	-1.90295800	-2.44921600	0.40872600
C	-3.04194500	-0.66274200	-0.10706200
C	0.45688600	-3.13538400	0.18942600
C	2.26112000	-2.20671600	-0.60589500
C	2.83923500	0.04735600	-1.42642300
C	1.76004900	1.93133600	-1.62636500
C	-0.60345900	2.60744100	-1.43843400
C	-2.46507600	1.58580800	-0.94372700

C	0.73708400	2.86545800	-1.68344300
H	1.00217400	3.87176200	-1.99043900
C	-3.36868700	0.62232500	-0.51652100
H	-4.41452200	0.90799400	-0.46948100
C	-0.86191200	-3.35292000	0.56115300
H	-1.10567600	-4.32468100	0.97818100
C	3.14467300	-1.27378300	-1.12976000
H	4.16932400	-1.59398300	-1.28796400
C	3.12718100	2.19803500	-2.00142500
C	3.79596000	1.02592200	-1.88678800
C	2.62454800	-3.55003400	-0.21863700
C	1.50612500	-4.12367400	0.28357500
C	-3.28093700	-2.72704100	0.74058800
C	-3.98922800	-1.62080800	0.41370100
C	-2.81724700	2.94439300	-1.28320100
C	-1.66038500	3.58033900	-1.58414200
N	0.25603100	0.31250800	1.18192300
C	1.44232400	0.23321300	1.87265300
C	-0.61450100	0.86327800	1.99466500
H	-1.64678100	1.07079800	1.76345700
C	1.28381500	0.74565100	3.12606800
N	-0.03576200	1.14092100	3.18082000
H	2.32685000	-0.18641000	1.42088700
C	2.22832900	0.90267100	4.26570600
H	3.20916400	0.51646200	3.98725100
H	2.34822700	1.95251500	4.54765800
H	1.88921700	0.35349600	5.14857700
H	-0.49467400	1.56645200	3.96840100
Fe	-0.08890500	-0.29055600	-0.68683000
H	-1.51716400	4.61067700	-1.88072900
H	-3.82244600	3.34375400	-1.28044700
H	-5.05279800	-1.45026700	0.51210100
H	-3.64213800	-3.65586400	1.16099800
H	1.38258500	-5.12332300	0.67766700
H	3.61239900	-3.97875200	-0.32006300
H	4.84012300	0.82643800	-2.08598200
H	3.50382200	3.15792000	-2.32694500
N	-0.35158200	-0.80945700	-2.56097800
N	-0.31441400	-0.97994100	-3.67042900
C	-0.19533700	-1.03652000	-4.96898500
C	0.54447600	0.02106700	-5.65165700
H	-0.64966300	-1.87382000	-5.47644400
O	0.67019300	0.00557700	-6.85891000
C	1.14797000	1.09174400	-4.74689200
C	1.69755900	2.29406500	-5.45688700
H	0.39680600	1.41203100	-4.01997800

H	1.93662800	0.60981100	-4.15557600
C	2.88480400	2.21709000	-6.18391400
C	1.03765100	3.51838900	-5.37772600
C	3.40019200	3.33918900	-6.81940400
H	3.40917600	1.26893600	-6.25010800
C	1.55033500	4.64512600	-6.01206700
H	0.11582300	3.59174200	-4.80734800
C	2.73400700	4.55809400	-6.73487100
H	4.32526400	3.26356700	-7.38167500
H	1.02459600	5.59173900	-5.93943300
H	3.13740700	5.43552800	-7.22959700

$^{1}Int_{N2}$

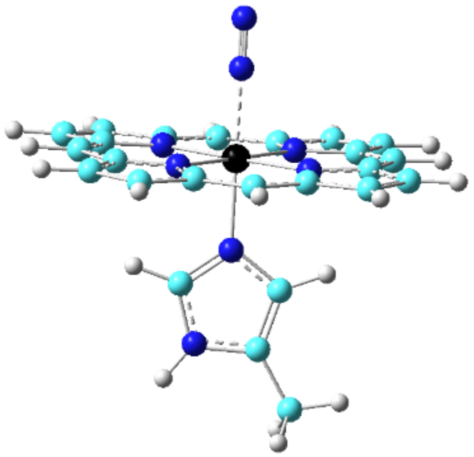

N	-1.45576391	-0.91792277	1.99883891
N	-1.04858873	-0.64305844	1.02147343
Fe	-0.31379802	-0.14709886	-0.74114891
N	-2.17580414	-0.19406892	-1.51655255
N	-0.54672086	1.80159466	-0.27699511
N	1.53793720	-0.10757862	0.05611528
N	-0.09085722	-2.10306847	-1.18418152
C	-2.81901293	-1.27179662	-2.04506249
C	-4.12932567	-0.88954087	-2.51544225
C	-4.25822085	0.43499376	-2.26666767
C	-3.02978159	0.85918771	-1.63778548
C	-2.77666894	2.15350790	-1.20863557
C	-1.62333044	2.58160448	-0.56864458
C	-1.40573303	3.92857081	-0.09615444
C	-0.18518363	3.94171650	0.48879720
C	0.34529755	2.60439313	0.36591014
C	1.59877155	2.21291765	0.81368365
C	2.15151603	0.95042886	0.65403015
C	3.48274576	0.58251790	1.07511820

C	3.65893626	-0.71018539	0.71463356
C	2.43232798	-1.13323624	0.08074744
C	2.20603298	-2.40961233	-0.41178807
C	1.02696458	-2.85543268	-0.99004255
C	0.80792600	-4.20339485	-1.45838527
C	-0.45986156	-4.24869154	-1.93111376
C	-1.01217401	-2.92612053	-1.75730003
C	-2.28861391	-2.54983641	-2.15069531
H	-3.55557111	2.89123514	-1.37232703
H	2.20652210	2.96263507	1.30974519
H	3.01565679	-3.12657289	-0.32133805
H	-2.91717695	-3.31388898	-2.59641864
H	-4.84309763	-1.56000603	-2.97490508
H	-5.10081783	1.07950312	-2.47786366
H	-2.10853800	4.74375149	-0.20325257
H	0.32517692	4.77014189	0.96124862
H	4.17864596	1.24188030	1.57602779
H	4.52929989	-1.33584471	0.85945734
H	1.53893613	-4.99962521	-1.41829977
H	-0.98720297	-5.08905780	-2.36207037
N	0.43209026	0.35735881	-2.52499014
C	1.35458726	1.34122246	-2.79383267
C	0.12837483	-0.19387186	-3.67646406
H	-0.56966051	-1.00236794	-3.82215039
C	1.61124630	1.38402690	-4.13221338
N	0.81830491	0.39556622	-4.67459658
H	1.76965930	1.95115651	-2.00762450
C	2.51139518	2.24429105	-4.94787490
H	3.02885330	2.95378766	-4.30162673
H	3.26810963	1.65330487	-5.47156042
H	1.95381197	2.81557126	-5.69547598
H	0.75959083	0.15066784	-5.64855942

^{1}Int$_{O1}$

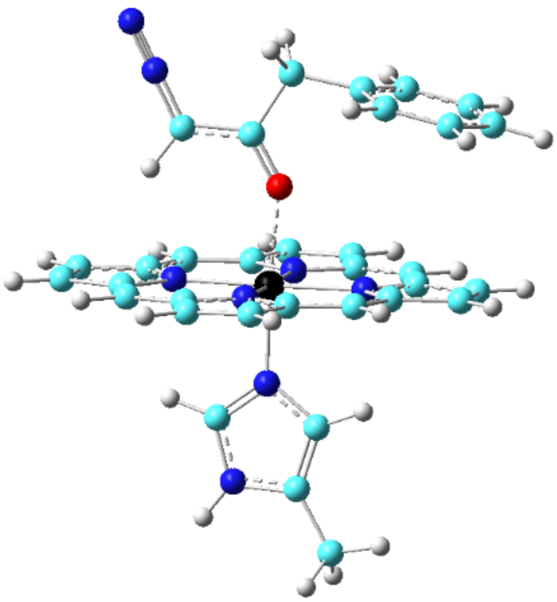

N	-1.58068400	-1.08577800	-0.37375400
N	-1.21379300	1.74326400	-0.40604800
N	1.60829700	1.38190000	-0.59290000
N	1.24281500	-1.44631300	-0.57248800
C	-1.56213500	-2.44922100	-0.39608800
C	-2.89053000	-0.72189900	-0.25937200
C	0.88046300	-2.75842200	-0.57106400
C	2.59812900	-1.42570200	-0.66763100
C	2.91556200	1.02003400	-0.67864700
C	1.58911600	2.74133100	-0.60200600
C	-0.85288900	3.05270800	-0.44870300
C	-2.57110700	1.72525500	-0.29776600
C	0.44918500	3.52630200	-0.53779600
H	0.58524000	4.60309300	-0.55566100
C	-3.35657900	0.58581400	-0.22149300
H	-4.42941800	0.72680100	-0.13454800
C	-0.41926000	-3.23272700	-0.49049900
H	-0.55660500	-4.30950100	-0.49963100
C	3.38511100	-0.28332900	-0.70959400
H	4.45906600	-0.42298500	-0.78309800
C	2.93570400	3.26280400	-0.67821000
C	3.76014200	2.19269300	-0.72599200
C	3.11847200	-2.77377400	-0.72673700
C	2.05074300	-3.60244900	-0.66789100
C	-2.90396000	-2.96675800	-0.30062700
C	-3.72986500	-1.89286800	-0.21310700
C	-3.09049500	3.07437600	-0.27603400

C	-2.02238700	3.89942300	-0.37145200
N	0.14075500	0.14621900	1.56308100
C	1.00939500	0.87384400	2.34448700
C	-0.59052900	-0.56234900	2.39163300
H	-1.37299800	-1.24756100	2.11213400
C	0.79733800	0.59871200	3.66330000
N	-0.22819100	-0.32014700	3.66967600
H	1.72860000	1.54584500	1.90648900
C	1.45083400	1.10027600	4.90316300
H	2.23449200	1.81289800	4.64427400
H	0.73766300	1.60885500	5.55818800
H	1.91069100	0.28798000	5.47333700
H	-0.64210600	-0.74242400	4.48338400
Fe	0.01735400	0.14955200	-0.45643300
H	-2.00760900	4.98113100	-0.38685600
H	-4.13752500	3.33540200	-0.19742200
H	-4.80830200	-1.88144400	-0.12721200
H	-3.16647100	-4.01637400	-0.29966900
H	2.03664200	-4.68412800	-0.68714200
H	4.16638000	-3.03145100	-0.80450500
H	4.83914100	2.17702900	-0.80140900
H	3.19341800	4.31313400	-0.70563200
O	0.02428000	0.23605200	-2.52218100
C	-0.55993800	-0.05221800	-3.55318000
C	-1.79389600	-0.79692900	-3.54863200
C	0.02688800	0.38416400	-4.89147800
N	-2.38414900	-1.08854300	-4.67890200
H	-2.28051900	-1.14037200	-2.64854000
C	1.28115700	1.19390100	-4.70655500
H	0.22458000	-0.51053100	-5.49236000
H	-0.73028200	0.95808500	-5.43694200
N	-2.88844400	-1.33857800	-5.64923800
C	1.20770700	2.57164100	-4.51608100
C	2.52519600	0.57140000	-4.65677200
C	2.35709900	3.31601300	-4.28644700
H	0.23988600	3.06461300	-4.53200700
C	3.67789000	1.31336800	-4.42958800
H	2.59237600	-0.50526100	-4.78312500
C	3.59623900	2.68795400	-4.24451900
H	2.28366500	4.38708000	-4.12935000
H	4.64040500	0.81418000	-4.38668000
H	4.49394300	3.26683500	-4.05510000

^{1}TS$_{O}$

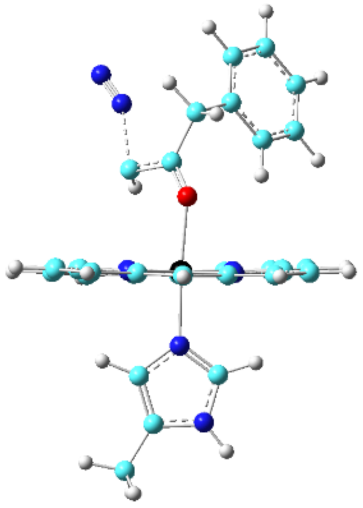

C	0.31885832	-0.85955214	-3.49417701
C	-0.00485595	0.52678792	-3.50493528
O	-0.27727267	1.09059091	-2.43589592
N	0.85510747	-1.30701931	-5.47169763
N	0.93303074	-1.94414840	-6.35599160
H	1.32790246	-1.16177797	-3.22476053
C	-0.12011912	1.33403937	-4.78335211
C	1.24841677	1.77177629	-5.26797242
C	2.19179386	2.26361961	-4.36390413
C	1.58356218	1.69832034	-6.61825692
C	3.44550766	2.66694163	-4.80388222
H	1.94957559	2.32738676	-3.30764219
C	2.83658413	2.10914672	-7.06117375
H	0.85988617	1.31588077	-7.33250614
C	3.77224355	2.59233920	-6.15422877
H	4.16677342	3.03633840	-4.08258637
H	3.08171774	2.04632307	-8.11632685
H	4.75196624	2.90757427	-6.49768586
H	-0.64341845	0.77318984	-5.56019683
H	-0.73092644	2.20364756	-4.52855606
N	-1.75143508	-0.02270376	-0.19793884
N	-0.54238646	2.56386277	-0.10845451
N	1.97590122	1.40426111	-0.75709540
N	0.76800782	-1.18130970	-0.82161464
C	-2.16228822	-1.31404328	-0.31309158
C	-2.85379830	0.69835524	0.14277689
C	0.02720313	-2.32916904	-0.81186904
C	2.06752605	-1.56862791	-0.99893340
C	3.09492175	0.67610851	-1.01775341
C	2.36548627	2.70921099	-0.74215313

C	0.18384561	3.71266493	-0.17340874
C	-1.80677961	2.93159578	0.22433719
C	1.53515662	3.78963289	-0.47741122
H	1.98516373	4.77724628	-0.49699603
C	-2.88529701	2.06727524	0.35586367
H	-3.84145897	2.50531962	0.62452627
C	-1.34198396	-2.39473629	-0.60124686
H	-1.80466989	-3.37554381	-0.64109685
C	3.14957468	-0.70915482	-1.10540475
H	4.12160445	-1.15667294	-1.28697622
C	3.77955037	2.81307270	-1.01601884
C	4.23360740	1.54808836	-1.18476331
C	2.14498046	-3.00211085	-1.12761145
C	0.87850521	-3.47287532	-1.01669892
C	-3.58000640	-1.41704687	-0.04360922
C	-4.00858649	-0.16723672	0.24374796
C	-1.88712475	4.36579515	0.38895028
C	-0.64992248	4.85241651	0.13644206
N	0.50659412	0.44300476	1.50447443
C	0.18240379	-0.63487016	2.29411844
C	1.12855054	1.29786713	2.28073917
H	1.51868819	2.25508539	1.97413429
C	0.61766127	-0.42638426	3.56982112
N	1.21853948	0.81372207	3.53812985
H	-0.34202538	-1.48650361	1.89067727
C	0.52991601	-1.25758405	4.80172955
H	0.01514313	-2.19274271	4.57933799
H	1.52085557	-1.50477851	5.19329357
H	-0.02689131	-0.74828590	5.59352460
H	1.65106102	1.28314067	4.31549539
Fe	0.11420002	0.69103247	-0.45066827
H	-0.31527406	5.88108345	0.15180081
H	-2.78320645	4.91128110	0.65309519
H	-5.00790912	0.15914167	0.49900094
H	-4.15193863	-2.33478463	-0.07275787
H	0.53823814	-4.49872065	-1.06019391
H	3.05745888	-3.56222091	-1.28244956
H	5.24057913	1.21912853	-1.40391768
H	4.33589956	3.73957788	-1.06594259

^{3}Int$_{O2}$

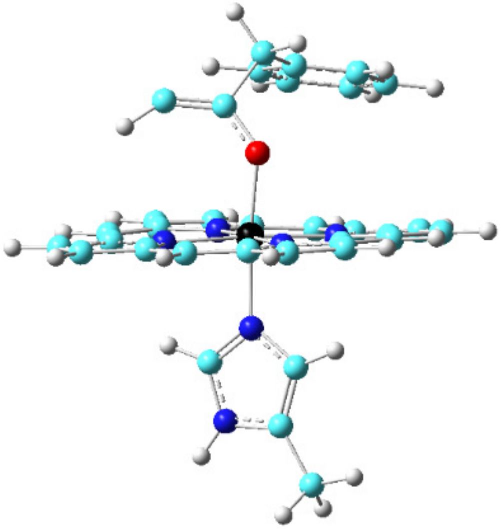

C	1.15790095	-1.26796119	-3.78371310
C	0.41692608	-0.18805549	-3.56920370
O	-0.20762278	0.16143827	-2.44835164
H	1.47627877	-2.15165995	-3.25343904
C	0.16907728	0.79354025	-4.70194924
C	0.88106403	2.11304132	-4.50196287
C	0.17712503	3.28888267	-4.25470640
C	2.27337810	2.17392302	-4.57252658
C	0.84262633	4.50132035	-4.10463545
H	-0.90504619	3.25388007	-4.16953103
C	2.94225461	3.38112806	-4.42745063
H	2.83456431	1.25703821	-4.73062552
C	2.22751962	4.55333926	-4.19834909
H	0.27473216	5.40534341	-3.90832243
H	4.02591927	3.40688431	-4.48719649
H	2.74878033	5.49901211	-4.08624626
H	0.49317391	0.34401884	-5.64531173
H	-0.90998851	0.96309294	-4.77138336
N	-1.62189945	-0.37733387	-0.23890548
N	-0.27083819	2.06786907	-0.59204481
N	2.17821423	0.64243895	-1.03962356
N	0.84641217	-1.79014366	-0.61010654
C	-2.08913073	-1.64408625	-0.07036726
C	-2.70189198	0.44546521	-0.14102567
C	0.03163966	-2.85555956	-0.36775595
C	2.09847006	-2.30031385	-0.79441044
C	3.24804435	-0.18173268	-1.22086507
C	2.65058428	1.91359561	-1.16422996
C	0.55341964	3.13366714	-0.77371940

C	-1.52480405	2.57340075	-0.44385422
C	1.91091282	3.07316704	-1.02720581
H	2.43173575	4.01246398	-1.17039384
C	-2.67068259	1.82572098	-0.23700663
H	-3.60971012	2.35895314	-0.13780240
C	-1.32897663	-2.79907486	-0.11857210
H	-1.84310949	-3.74034003	0.04245758
C	3.22831694	-1.55936531	-1.09138709
H	4.15989859	-2.09463015	-1.23752789
C	4.06105726	1.89254243	-1.45270401
C	4.43234622	0.59207567	-1.49104524
C	2.07449992	-3.73263845	-0.66745833
C	0.79091577	-4.07763329	-0.40492674
C	-3.51331917	-1.62322262	0.15213038
C	-3.89423089	-0.32554061	0.10440497
C	-1.49294440	4.01280271	-0.51833631
C	-0.20066694	4.36028870	-0.71702233
N	0.65807004	0.25230656	1.30867486
C	-0.16783869	0.77354394	2.27858687
C	1.74768158	-0.15542050	1.92025890
H	2.60075109	-0.61525180	1.44907894
C	0.43471826	0.67666577	3.49574794
N	1.65339331	0.08136762	3.24035523
H	-1.13505500	1.18143169	2.03541332
C	-0.00459496	1.07804062	4.85954778
H	-0.99794063	1.52434642	4.81003800
H	-0.05486524	0.21911614	5.53422050
H	0.67387521	1.81404472	5.29954956
H	2.35888907	-0.13990896	3.92311002
Fe	0.27403698	0.13240333	-0.67061389
H	0.22137064	5.34837923	-0.83714214
H	-2.35890205	4.65467876	-0.43127885
H	-4.88552552	0.09043501	0.22109261
H	-4.12586655	-2.49967414	0.31333403
H	0.37825016	-5.06507407	-0.25006364
H	2.93653357	-4.37649158	-0.77557343
H	5.41215222	0.17521797	-1.67936076
H	4.66878372	2.77256587	-1.61079013

$^{1}TS_{Bridging}$

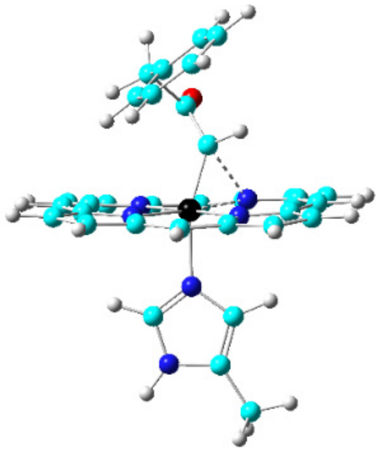

C	32.38743800	-0.05179800	7.99839200
C	33.26982600	1.11132100	8.28934600
N	34.08986300	1.69055000	7.34465400
C	33.48543900	1.83407800	9.42607000
C	34.75381300	2.71193900	7.92710500
N	34.40870100	2.82415300	9.18701800
N	34.90350700	5.65776500	9.25392000
N	35.44566900	2.73112900	11.95458100
C	37.82749200	2.81131500	10.14898300
C	35.83801500	6.16764200	8.40361200
C	32.36230300	5.34311800	10.78647500
C	34.53724100	2.29855500	12.89667000
C	39.04599100	2.88646500	9.37919200
C	35.29021100	7.27722200	7.66311900
C	31.14094000	5.26127900	11.55271300
C	35.06904900	1.14895300	13.57721700
C	38.94331300	3.98862100	8.59920900
C	34.00490000	7.41417000	8.06964500
C	31.34466800	4.31829800	12.50156400
C	36.26255200	0.86046100	13.00470700
C	35.81155900	4.77719400	12.13650300
C	37.19547600	5.09143100	12.51574200
C	37.45455800	6.59661800	12.34306800
C	36.24151800	7.46823600	12.56899600
C	35.54223400	8.02267500	11.49981300
C	34.38610500	8.76311100	11.71502800
C	33.91464100	8.96180600	13.00679300
C	34.61276100	8.42409500	14.08391800
C	35.76802400	7.68512900	13.86452800
C	37.65082100	4.56720100	8.87761700
C	33.77752900	6.39737000	9.06586100

C	32.68777500	3.82198800	12.30553200
C	36.49540000	1.83954000	11.97804200
C	37.57950900	1.85552100	11.13013700
C	37.12590000	5.67537700	8.23586000
C	32.58475700	6.24009200	9.75909800
C	33.27479800	2.82230100	13.08235200
N	36.99822500	3.83278100	9.82317400
N	33.27540800	4.44892400	11.25745800
O	38.07487300	4.33894100	12.86792200
Fe	35.14303200	4.16586000	10.55072400
H	31.83193600	-0.32671600	8.89546400
H	31.66274300	0.17856000	7.21220900
H	33.03850500	1.71012200	10.40011500
H	35.46642600	3.33768900	7.41368300
H	37.81998500	6.71332400	11.31682500
H	38.27412800	6.85451900	13.01854200
H	35.89436300	7.85786000	10.48710700
H	33.84944900	9.17345500	10.86611800
H	33.00786300	9.53321800	13.17590800
H	34.25467600	8.57852300	15.09659200
H	36.30475800	7.25976200	14.70854600
H	38.33133600	1.08763000	11.27699600
H	37.75814800	6.17855500	7.51134400
H	31.76965400	6.90690000	9.49663500
H	32.67199600	2.39117800	13.87457500
H	32.96329900	-0.92519400	7.67926800
H	34.17901100	1.40657700	6.38360800
H	39.66137900	4.38221000	7.89250700
H	39.87019400	2.19019500	9.45358200
H	35.82916800	7.85788400	6.92678700
H	33.26800000	8.13329300	7.73828100
H	30.25842700	5.86213700	11.37988200
H	30.66447000	3.97985100	13.27137600
H	34.56419700	0.61903400	14.37325200
H	36.93648300	0.04829500	13.24039700
H	35.10512300	4.94663300	12.95436000

^{1}IPC$_{Bridging}$

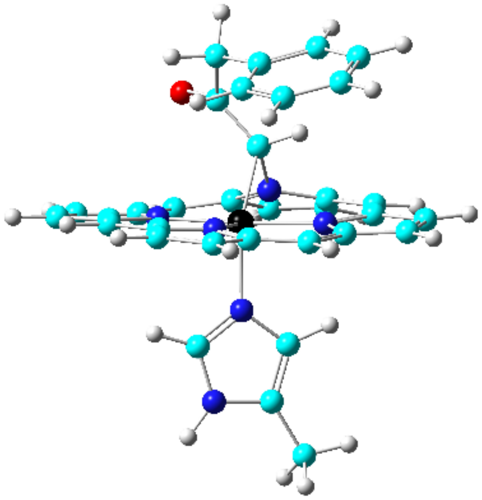

C	32.40657600	0.04007700	7.89876800
C	33.38967500	1.10001900	8.25338300
N	34.38874200	1.51752400	7.40124300
C	33.55132700	1.85720000	9.37636700
C	35.10183800	2.48233000	8.01906100
N	34.61844700	2.71133800	9.21777100
N	35.14264800	5.51380900	9.31186800
N	35.73762900	2.78041300	12.35118700
C	38.08514200	2.75366600	10.40617200
C	36.08656700	5.99709800	8.45984500
C	32.55828800	5.20270200	10.77785400
C	34.67235700	2.19966900	13.07298600
C	39.33242900	2.82990200	9.68310300
C	35.54567600	7.06916700	7.66489300
C	31.32695300	5.14697200	11.52510700
C	35.11286600	0.93358800	13.56470700
C	39.22340800	3.87931100	8.83558500
C	34.24720100	7.20276100	8.03455400
C	31.51695000	4.23742100	12.50999000
C	36.35192700	0.69142900	13.06736100
C	35.96186700	4.23840900	12.48090900
C	37.23350000	4.79310600	12.84021400
C	37.23536400	6.32407200	13.04014700
C	35.92988200	7.06096000	12.90331400
C	35.62024400	7.76507000	11.74124100
C	34.39703300	8.40947700	11.60205300
C	33.45510300	8.35005700	12.62194900
C	33.75259900	7.65524200	13.78947100
C	34.98448200	7.02872400	13.93056000
C	37.89947100	4.42131400	9.02650600

C	34.01062600	6.22225400	9.06135000
C	32.86362900	3.73884400	12.35623100
C	36.74703200	1.79880800	12.25380600
C	37.82199300	1.81449200	11.41224700
C	37.37816300	5.49288200	8.33194000
C	32.79991200	6.06118000	9.72799300
C	33.40888100	2.71666900	13.15738200
N	37.22717300	3.70483000	9.98244600
N	33.47090100	4.32538800	11.30085600
O	38.30484700	4.20999000	12.96153500
Fe	35.33346200	4.00794900	10.58145200
H	31.71606500	-0.11494800	8.72822000
H	31.81804800	0.31511100	7.01904400
H	32.97045600	1.84994400	10.28517700
H	35.94652500	2.98483900	7.57593400
H	37.96387500	6.70817500	12.31992400
H	37.66903900	6.49254600	14.03148900
H	36.33525400	7.78819900	10.92391500
H	34.17511800	8.94315300	10.68380400
H	32.49352400	8.84009500	12.50808800
H	33.02537800	7.60437900	14.59356700
H	35.21253400	6.48982300	14.84661900
H	38.54202000	1.01314400	11.53860600
H	38.02498800	5.97405800	7.60498700
H	31.98312200	6.70731500	9.42191900
H	32.73055200	2.22657800	13.84792100
H	32.89976200	-0.91323800	7.68923300
H	34.55939900	1.16842700	6.47313900
H	39.95998700	4.26722900	8.14534700
H	40.18053600	2.17761700	9.83971100
H	36.09454000	7.62857700	6.91960100
H	33.50957800	7.89870900	7.65843500
H	30.44848800	5.74354200	11.32060800
H	30.82520400	3.92657500	13.28105000
H	34.49445900	0.26302900	14.14475000
H	36.93345200	-0.21443400	13.16789200
H	35.12195500	4.69084600	12.98586300

H_3O^+

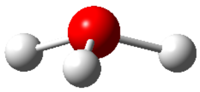

O	-0.64926700	-3.20836900	-0.03947000
H	0.28053200	-3.29475400	-0.30232600
H	-1.03725500	-2.36063600	-0.30857800
H	-1.18766000	-3.96912900	-0.30961400

H_2O

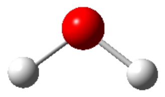

O	-0.64980100	-3.20833000	0.00000000
H	0.30753900	-3.17784400	0.00000000
H	-0.94062000	-2.29573700	0.00000000

$^1Int_{N\text{-}AlkylationOH2}$

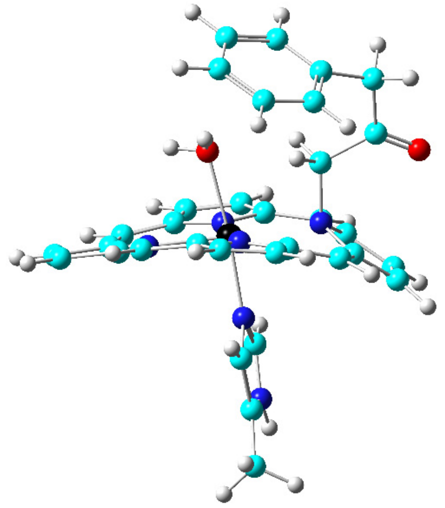

C	32.27164200	0.08592500	8.22059000
C	33.40284300	0.97723200	8.59545100
N	34.72354800	0.65052100	8.38230600
C	33.43871500	2.20871000	9.17824000
C	35.49350600	1.66235100	8.82527000
N	34.74484500	2.62477500	9.31928600
N	34.82429400	5.39733500	8.46138000
N	35.96785600	3.27811800	11.92223200
C	38.26118000	3.47652700	9.94096000
C	35.57795300	5.62805000	7.35638100
C	32.60596700	5.52331200	10.45754400
C	34.90801300	2.52855300	12.53263000
C	39.44207700	3.58240400	9.11219500
C	34.83343300	6.41090100	6.40247000
C	31.46060500	5.50055600	11.33651000
C	35.38774200	1.23752000	12.83499300
C	39.08305400	4.28661800	8.01531500
C	33.63253200	6.67621500	6.97548000
C	31.74381800	4.61333400	12.31800400
C	36.67913200	1.13329400	12.39541100

C	36.31091500	4.47836600	12.73242600
C	36.64454300	4.27759000	14.20216000
C	36.51589100	5.53568900	15.07150200
C	35.69743600	6.64641400	14.46398000
C	36.30935000	7.71722900	13.81686100
C	35.55029100	8.65766900	13.12668000
C	34.16630400	8.53163300	13.06965200
C	33.54320200	7.47665200	13.73206200
C	34.30382200	6.54916700	14.43060800
C	37.68509900	4.61468300	8.17344300
C	33.64719000	6.04648100	8.27151300
C	33.07256500	4.11559800	12.05370200
C	37.04796600	2.35002100	11.78397300
C	38.16960400	2.59686700	11.03013000
C	36.91893600	5.26652200	7.22464700
C	32.62745700	6.13717200	9.21802200
C	33.64850900	3.03284500	12.73447500
N	37.22213800	4.13487200	9.36793200
N	33.58977700	4.70693000	10.94684500
O	37.01972100	3.23514600	14.67124100
Fe	35.36600200	4.37097500	10.07381700
H	31.32534500	0.56255100	8.47694000
H	32.26419900	-0.12184700	7.14741800
H	32.61339900	2.82179000	9.50009800
H	36.56902400	1.66408200	8.76871400
H	37.53747200	5.87098600	15.27931100
H	36.10309800	5.20255600	16.02730800
H	37.39131800	7.80694200	13.83112500
H	36.04270900	9.48434600	12.62588000
H	33.57391900	9.25811700	12.52412800
H	32.46488100	7.37151500	13.69598800
H	33.81330000	5.71680400	14.92720700
H	39.01122200	1.92837400	11.17716400
H	37.41570700	5.55148100	6.30323300
H	31.75293500	6.71659700	8.94107100
H	32.99861600	2.47539500	13.40001900
H	32.32202300	-0.87000500	8.74869200
H	35.06348300	-0.19646200	7.95810300
H	39.69004400	4.55631600	7.16247700
H	40.40395400	3.14897300	9.34758300
H	35.19967500	6.72513300	5.43521200
H	32.80951700	7.24947300	6.57259700
H	30.55259500	6.06780300	11.18687200
H	31.11998200	4.30455500	13.14503700
H	34.78790200	0.45689100	13.28037500
H	37.30465100	0.25246000	12.41996100

H	35.46150100	5.14591500	12.66977600
O	36.01774000	6.28577400	10.71440000
H	36.26402600	6.75491300	9.91279700
H	35.33083300	6.80963800	11.14192100
H	37.14071100	5.00401800	12.25874700

$^{1}P_{N\text{-}Alkylation}$

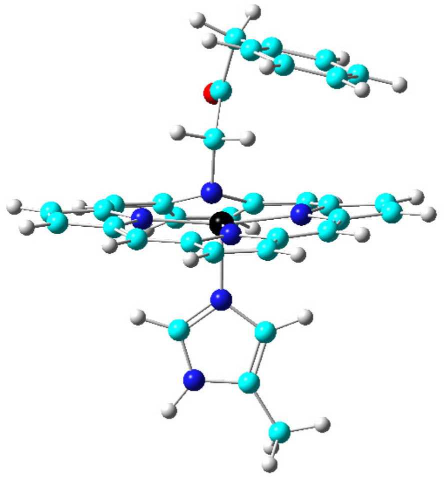

C	32.13290200	0.14180400	8.02819300
C	33.28122200	0.99717300	8.43290400
N	34.57998300	0.74909900	8.04741100
C	33.35282600	2.12158800	9.19956500
C	35.37592000	1.69884500	8.57077000
N	34.66107700	2.54837900	9.27865000
N	34.89738300	5.46454400	8.83313700
N	35.96503900	3.05695500	12.02328600
C	38.22054700	3.18060300	10.03465500
C	35.68633900	5.81962000	7.78203500
C	32.60324800	5.36885600	10.72478500
C	34.95353400	2.40089400	12.79132900
C	39.39191500	3.29937300	9.20335600
C	34.99519300	6.75039400	6.92812200
C	31.47366000	5.34041400	11.62060600
C	35.43724700	1.13697200	13.18322500
C	39.07055400	4.15110400	8.20213500
C	33.78021400	6.96014100	7.48978000
C	31.77330200	4.45255800	12.59494600
C	36.68806200	0.96252100	12.65807800
C	36.36564100	4.39597400	12.52865800
C	36.71217600	4.50470800	14.01114600
C	36.81482000	5.93739600	14.52670600
C	35.81761900	6.86308500	13.87219100

C	36.19524500	7.69311700	12.81768800
C	35.24260800	8.41893200	12.11133700
C	33.89963800	8.32728900	12.45842500
C	33.51637700	7.51987100	13.52364900
C	34.46938300	6.79223300	14.22496300
C	37.70283300	4.54932200	8.41994000
C	33.73400100	6.15314400	8.68145200
C	33.09057600	3.94651500	12.29811900
C	37.02888300	2.10916400	11.91358900
C	38.11982300	2.28003000	11.09814600
C	36.99291700	5.39234000	7.58548500
C	32.66192300	6.11471100	9.56540600
C	33.70188200	2.91791800	13.01671500
N	37.20801300	3.95466000	9.54869900
N	33.57981600	4.51817200	11.16403500
O	36.90996400	3.54278800	14.70241800
Fe	35.32177800	4.12667800	10.22965700
H	31.20992700	0.53913400	8.45027600
H	32.02116600	0.11065500	6.94127900
H	32.55546100	2.65105300	9.69361000
H	36.44078600	1.74402800	8.41666400
H	35.55454400	5.10102900	12.33101400
H	37.23430800	4.74322800	11.96231800
H	37.83967400	6.27318000	14.33091400
H	36.69549800	5.89360200	15.61090900
H	37.24110700	7.75527500	12.53099400
H	35.55000500	9.05237800	11.28631600
H	33.15444200	8.88576100	11.90250700
H	32.47098900	7.44450700	13.80064000
H	34.16085100	6.14573500	15.04161600
H	38.93962200	1.58253400	11.22895700
H	37.50937200	5.77282900	6.71079400
H	31.80166600	6.73093700	9.32779300
H	33.09695000	2.41495200	13.76283800
H	32.25353500	-0.88453900	8.38453300
H	34.88902300	-0.01045000	7.46348800
H	39.69024100	4.48636500	7.38249600
H	40.32844400	2.78878900	9.37785500
H	35.40338100	7.18534700	6.02680500
H	32.98260100	7.60287200	7.14483500
H	30.57425200	5.92748100	11.49981800
H	31.17401700	4.15620200	13.44414200
H	34.86074100	0.41806900	13.74707200
H	37.30301800	0.07623100	12.71765400

$^{5}P_{N\text{-}Alkylation}$

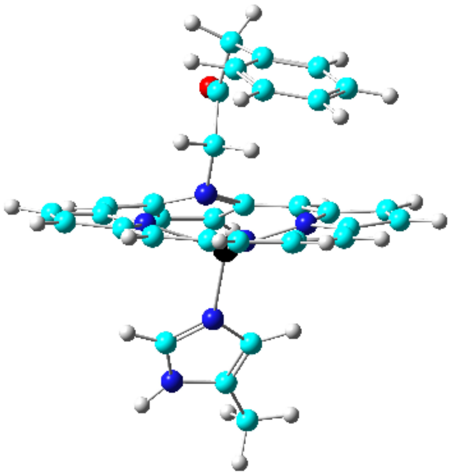

C	32.53165900	-0.94104000	8.83612400
C	33.46567900	0.21640200	8.83560700
N	34.73560000	0.15998900	8.29825500
C	33.34367500	1.48704700	9.30903400
C	35.32706700	1.35372900	8.45495000
N	34.50844200	2.18714500	9.06668200
N	34.83285400	5.93645100	8.98968200
N	35.73933700	2.94199400	11.89525400
C	38.04692600	3.28565900	9.93071600
C	35.75515200	6.53338400	8.18680500
C	32.45669100	5.55840100	10.77369300
C	34.68274500	2.24831300	12.51090400
C	39.26721700	3.52823300	9.20254000
C	35.20834300	7.75167700	7.63832400
C	31.31293500	5.45954400	11.64835100
C	35.10212300	0.91117500	12.71040400
C	39.03281300	4.58925500	8.39073700
C	33.95554800	7.87864500	8.14333700
C	31.54577200	4.41913200	12.48404800
C	36.36122800	0.76904400	12.19441900
C	36.08783200	4.27234900	12.40560500
C	36.42880200	4.33865700	13.88497500
C	36.67121600	5.75352800	14.40683000
C	35.95150500	6.80989600	13.60313700
C	36.58513600	7.45952700	12.54470900
C	35.87119300	8.30161100	11.69963700
C	34.51508400	8.51445400	11.91373400
C	33.87778800	7.88783000	12.97925100
C	34.59174600	7.04062800	13.81708800
C	37.66296300	4.98580700	8.60562300
C	33.73804600	6.73976900	9.00236100

C	32.84038100	3.88626200	12.13290600
C	36.76806700	2.01294500	11.65302900
C	37.87926800	2.25739900	10.86546300
C	37.04972100	6.06652800	7.97701000
C	32.61250500	6.53294300	9.79654200
C	33.42216100	2.77146200	12.74331000
N	37.08522300	4.17079900	9.53701200
N	33.36475900	4.58690800	11.08677800
O	36.51835000	3.36085600	14.57893600
Fe	34.98762700	4.05555700	9.83140800
H	31.58224400	-0.64892000	9.28431500
H	32.33191700	-1.29280500	7.82094500
H	32.50685700	1.94650700	9.81085500
H	36.32900500	1.58121500	8.12785700
H	35.26300800	4.95855300	12.21589000
H	36.93209700	4.66873100	11.84042200
H	37.75517100	5.91315700	14.37774000
H	36.38029200	5.76162500	15.45938800
H	37.64165200	7.28421500	12.36174900
H	36.37262100	8.78392600	10.86735700
H	33.95578300	9.16305400	11.24861700
H	32.81875800	8.04781500	13.15067500
H	34.08348700	6.53366800	14.63248100
H	38.67460800	1.52160800	10.91997900
H	37.66491600	6.63315600	7.28548700
H	31.80238000	7.24668200	9.69071900
H	32.78448100	2.19755500	13.40730800
H	32.93458700	-1.77844800	9.41134200
H	35.15145200	-0.64209800	7.85284100
H	39.71909200	5.06896100	7.70702400
H	40.18481500	2.96946500	9.32299000
H	35.72903700	8.42407500	6.97107600
H	33.24598000	8.67617900	7.97272300
H	30.45081800	6.11127200	11.61896000
H	30.91701300	4.04860900	13.28140500
H	34.47635600	0.13412000	13.12441700
H	36.93209100	-0.14470400	12.11375600

Supplementary References

1 Nam, D., Steck, V., Potenzino, R. J. & Fasan, R. A Diverse Library of Chiral Cyclopropane Scaffolds via Chemoenzymatic Assembly and Diversification of Cyclopropyl Ketones. *J. Am. Chem. Soc.* **143**, 2221-2231, (2021).

2 Yang, H. J., Park, K. H., Shina, S., Lee, J. H., Park, S., Kim, H. S. & Kim, J. Characterization of heme ions using MALDI-TOF MS and MALDI FT-ICR MS. *International Journal of Mass Spectrometry* **343**, 37-44, (2013).

3 Tian, S., Fan, R., Albert, T., Khade, R. L., Dai, H., Harnden, K. A., Hosseinzadeh, P., Liu, J., Nilges, M. J., Zhang, Y., Moënne-Loccoz, P., Guo, Y. & Lu, Y. Stepwise nitrosylation of the nonheme iron site in an engineered azurin and a molecular basis for nitric oxide signaling mediated by nonheme iron proteins. *Chemical Science* **12**, 6569–6579, (2021).

4 Khade, R. L., Fan, W., Ling, Y., Yang, L., Oldfield, E. & Zhang, Y. Iron Porphyrin Carbenes as Catalytic Intermediates: Structures, Mossbauer and NMR Spectroscopic Properties, and Bonding. *Angewandte Chemie International Edition* **53**, 7574-7578, (2014).

5 Chakraborty, S., Reed, J., Ross, M., Nilges, M. J., Petrik, I. D., Ghosh, S., Hammes-Schiffer, S., Sage, J. T., Zhang, Y., Schultz, C. E. & Lu, Y. Spectroscopic and Computational Study of a Nonheme Iron Nitrosyl Center in a Biosynthetic Model of Nitric Oxide Reductase. *Angewandte Chemie-International Edition* **53**, 2417-2421, (2014).

6 Zhang, Y., Mao, J. H., Godbout, N. & Oldfield, E. Mossbauer quadrupole splittings and electronic structure in heme proteins and model systems: A density functional theory investigation. *Journal of the American Chemical Society* **124**, 13921-13930, (2002).

7 Zhang, Y., Mao, J. H. & Oldfield, E. Fe-57 Mossbauer isomer shifts of heme protein model systems: Electronic structure calculations. *Journal of the American Chemical Society* **124**, 7829-7839, (2002).

8 Zhang, Y., Gossman, W. & Oldfield, E. A density functional theory investigation of Fe-N-O bonding in heme proteins and model systems. *Journal of the American Chemical Society* **125**, 16387-16396, (2003).

9 Zhang, Y. & Oldfield, E. An investigation of the unusual Fe-57 Mossbauer quadrupole splittings and isomer shifts in 2 and 3-coordinate Fe(II) complexes. *Journal of Physical Chemistry B* **107**, 7180-7188, (2003).

10 Zhang, Y. & Oldfield, E. Fe-57 Mossbauer quadrupole splittings and isomer shifts in spin-crossover complexes: A density functional theory investigation. *Journal of Physical Chemistry A* **107**, 4147-4150, (2003).

11 Zhang, Y. & Oldfield, E. On the Mossbauer spectra of isopenicillin N synthase and a model {FeNO}(7) (S=3/2) system. *Journal of the American Chemical Society* **126**, 9494-9495, (2004).

12 Zhang, Y. & Oldfield, E. Cytochrome P450: An investigation of the Mossbaluer spectra of a reaction intermediate and an Fe(IV)=O model system. *Journal of the American Chemical Society* **126**, 4470-4471, (2004).

13 Ling, Y. & Zhang, Y. Mossbauer, NMR, Geometric, and Electronic Properties in S=3/2 Iron Porphyrins. *Journal of the American Chemical Society* **131**, 6386-6388, (2009).

14 Ling, Y., Davidson, V. L. & Zhang, Y. Unprecedented Fe(IV) Species in a Diheme Protein MauG: A Quantum Chemical Investigation on the Unusual Mossbauer Spectroscopic Properties. *Journal of Physical Chemistry Letters* **1**, 2936-2939, (2010).

15 Ling, Y. & Zhang, Y. in *Annual Reports in Computational Chemistry* Vol. 6 *Quantum Chemistry* (ed R. A. Wheeler) Ch. 5, 65-77 (Elsevier, 2010).

16 Fu, R., Gupta, R., Geng, J., Dornevil, K., Wang, S., Zhang, Y., Hendrich, M. P. & Liu, A. Enzyme Reactivation by Hydrogen Peroxide in Heme-based Tryptophan Dioxygenase. *Journal of Biological Chemistry* **286**, 26541-26554, (2011).

17 Katigbak, J. & Zhang, Y. Iron Binding Site in a Global Regulator in Bacteria - Ferric Uptake Regulator (Fur) Protein: Structure, Mössbauer Properties, and Functional Implication. *Journal of Physical Chemistry Letters* **3**, 3503-3508, (2012).

18 Yang, L., Ling, Y. & Zhang, Y. HNO Binding in a Heme Protein: Structures, Spectroscopic Properties, and Stabilities. *Journal of the American Chemical Society* **133**, 13814-13817, (2011).

19 Sharon, D. A., Mallick, D., Wang, B. & Shaik, S. Computation Sheds Insight into Iron Porphyrin Carbenes' Electronic Structure, Formation, and N−H Insertion Reactivity. *Journal of the American Chemical Society* **138**, 9597-9610, (2016).

20 Dzik, W. I., Xu, X., Zhang, X. P., Reek, J. N. H. & de Bruin, B. 'Carbene Radicals' in CoII(por)-Catalyzed Olefin Cyclopropanation. *Journal of the American Chemical Society* **132**, 10891-10902, (2010).

21 Gaussian Program v. Frisch, M. J.; Trucks, G. W.; Schlegel, H. B.; Scuseria, G. E.; Robb, M. A.; Cheeseman, J. R.; Scalmani, G.; Barone, V.; Petersson, G. A.; Nakatsuji, H.; Li, X.; Caricato, M.; Marenich, A. V.; Bloino, J.; Janesko, B. G.; Gomperts, R.; Mennucci, B.; Hratchian, H. P.; Ortiz, J. V.; Izmaylov, A. F.; Sonnenberg, J. L.; Williams-Young, D.; Ding, F.; Lipparini, F.; Egidi, F.; Goings, J.; Peng, B.; Petrone, A.; Henderson, T.; Ranasinghe, D.; Zakrzewski, V. G.; Gao, J.; Rega, N.; Zheng, G.; Liang, W.; Hada, M.; Ehara, M.; Toyota, K.; Fukuda, R.; Hasegawa, J.; Ishida, M.; Nakajima, T.; Honda, Y.; Kitao, O.; Nakai, H.; Vreven, T.; Throssell, K.; Montgomery, J. A., Jr.; Peralta, J. E.; Ogliaro, F.; Bearpark, M. J.; Heyd, J. J.; Brothers, E. N.; Kudin, K. N.; Staroverov, V. N.; Keith, T. A.; Kobayashi, R.; Normand, J.; Raghavachari, K.; Rendell, A. P.; Burant, J. C.; Iyengar, S. S.; Tomasi, J.; Cossi, M.; Millam, J. M.; Klene, M.; Adamo, C.; Cammi, R.; Ochterski, J. W.; Martin, R. L.; Morokuma, K.; Farkas, O.; Foresman, J. B.; Fox, D. J.; Gaussian 16, Revision C.01. (Gaussian, Inc., Wallingford CT, 2019).

22 Mennucci, B. & Tomasi, J. Continuum solvation models: A new approach to the problem of solute's charge distribution and cavity boundaries. *Journal of Chemical Physics* **106**, 5151-5158, (1997).

23 Torres, R. A., Lovell, T., Noodleman, L. & Case, D. A. Density Functional and Reduction Potential Calculations of Fe4S4 Clusters. *Journal of the American Chemical Society* **125**, 1923-1936, (2003).

24 Chai, J.-D. & Head-Gordon, M. Long-range corrected hybrid density functionals with damped atom-atom dispersion corrections. *Physical Chemistry Chemical Physics* **10**, 6615-6620, (2008).

25 Yang, K., Zheng, J., Zhao, Y. & Truhlar, D. G. Tests of the RPBE, revPBE, tHCTHhyb, wB97X-D, and MOHLYP density functional approximations and 29 others against representative databases for diverse bond energies and barrier heights in catalysis. *Journal of Chemical Physics* **132**, 164117, (2010).

26 Khade, R. L. & Zhang, Y. Catalytic and Biocatalytic Iron Porphyrin Carbene Formation: Effects of Binding Mode, Carbene Substituent, Porphyrin Substituent, and Protein Axial Ligand. *Journal of the American Chemical Society* **137**, 7560-7563, (2015).

27 Khade, R. L. & Zhang, Y. C-H Insertions by Iron Porphyrin Carbene: Basic Mechanism and Origin of Substrate Selectivity. *Chemistry - A Euopean Journal* **23**, 17654-17658 (2017).

28 Wei, Y., Tinoco, A., Steck, V., Fasan, R. & Zhang, Y. Cyclopropanations via Heme Carbenes: Basic Mechanism and Effects of Carbene Substituent, Protein Axial Ligand, and Porphyrin Substitution. *Journal of the American Chemical Society* **140**, 1649-1662 (2018).

29 Tinoco, A., Wei, Y., Bacik, J. P., Moore, E. J., Ando, N., Zhang, Y. & Fasan, R. Origin of high stereocontrol in olefin cyclopropanation catalyzed by an engineered carbene transferase. *ACS Catalysis* **9**, 1514-1524 (2019).

30 Vargas, D. A., Khade, R. L., Zhang, Y. & Fasan, R. Biocatalytic strategy for highly diastereo- and enantioselective synthesis of 2,3-dihydrobenzofuran based tricyclic scaffolds. *Angewandte Chemie International Edition* **58**, 10148-10152, (2019).

31 Khade, R., L., Chandgude, A. L., Fasan, R. & Zhang, Y. Mechanistic Investigation of Biocatalytic Heme Carbenoid Si-H Insertions. *ChemCatChem* **11**, 3101-3108, (2019).

32 Hay, P. J. & Wadt, W. R. Ab initio effective core potentials for molecular calculations. Potentials for the transition metal atoms scandium to mercury. *Journal of Chemical Physics* **82**, 270-283, (1985).

33 Wang, J.-C., Xu, Z.-J., Guo, Z., Deng, Q.-H., Zhou, C.-Y., Wan, X.-L. & Che, C.-M. Highly enantioselective intermolecular carbene insertion to C–H and Si–H bonds catalyzed by a chiral iridium(III) complex of a D4-symmetric Halterman porphyrin ligand. *Chemical Communications* **48**, 4299-4301, (2012).

34 Chan, K.-H., Guan, X., Lo, V. K.-Y. & Che, C.-M. Elevated Catalytic Activity of Ruthenium(II)–Porphyrin-Catalyzed Carbene/Nitrene Transfer and Insertion Reactions with N-Heterocyclic Carbene Ligands. *Angewandte Chemie-International Edition* **53**, 2982-2987, (2014).

35 Nakamura, E., Yoshikai, N. & Yamanaka, M. Mechanism of C-H bond activation/C-C bond formation reaction between diazo compound and alkane catalyzed by dirhodium tetracarboxylate. *Journal of the American Chemical Society* **124**, 7181-7192, (2002).

36 Zhang, Y., Mao, J. & Oldfield, E. ^{57}Fe Mössbauer Isomer Shifts of Heme Protein Model Systems: Electronic Structure Calculations. *Journal of the American Chemical Society* **124**, 7829-7839, (2002).

37 Becke, A. D. Density-functional thermochemistry. III. The role of exact exchange. *The Journal of Chemical Physics* **98**, 5648-5652, (1993).

38 Wachters, A. J. H. Gaussian Basis Set for Molecular Wavefunctions Containing Third-Row Atoms. *The Journal of Chemical Physics* **52**, 1033-1036, (1970).

39 Dufek, P., Blaha, P. & Schwarz, K. Determination of the Nuclear Quadrupole Moment of 57Fe. *Physical Review Letters* **75**, 3545-3548, (1995).

40 Biegler–König;, F., Schönbohm;, J. & Bayles., D. AIM2000—A Program to Analyze and Visualize Atoms in Molecules. *Journal of Computational Chemistry* **22**, 545-559, (2001).
