# Supplementary material for: Mechanistic manifold in a hemoprotein-catalyzed cyclopropanation reaction with diazoketone
Source: Nat Commun. 2023 Dec 2;14:7985. doi: 10.1038/s41467-023-43559-7 (PMC10693563; doi:10.1038/s41467-023-43559-7)
Supplement: Supplementary file 3 — Reporting Summary [file 41467_2023_43559_MOESM3_ESM.pdf]

nature research

Corresponding author(s): Rudi Fasan

Last updated by author(s): 2022/11/04

Reporting Summary

Nature Research wishes to improve the reproducibility of the work that we publish. This form provides structure for consistency and transparency in reporting. For further information on Nature Research policies, see our Editorial Policies and the Editorial Policy Checklist.

Statistics

For all statistical analyses, confirm that the following items are present in the figure legend, table legend, main text, or Methods section.

n/a	Confirmed	
☐	☒	The exact sample size (*n*) for each experimental group/condition, given as a discrete number and unit of measurement
☐	☒	A statement on whether measurements were taken from distinct samples or whether the same sample was measured repeatedly
☒	☐	The statistical test(s) used AND whether they are one- or two-sided<br>*Only common tests should be described solely by name; describe more complex techniques in the Methods section.*
☒	☐	A description of all covariates tested
☒	☐	A description of any assumptions or corrections, such as tests of normality and adjustment for multiple comparisons
☐	☒	A full description of the statistical parameters including central tendency (e.g. means) or other basic estimates (e.g. regression coefficient) AND variation (e.g. standard deviation) or associated estimates of uncertainty (e.g. confidence intervals)
☒	☐	For null hypothesis testing, the test statistic (e.g. *F*, *t*, *r*) with confidence intervals, effect sizes, degrees of freedom and *P* value noted<br>*Give P values as exact values whenever suitable.*
☒	☐	For Bayesian analysis, information on the choice of priors and Markov chain Monte Carlo settings
☒	☐	For hierarchical and complex designs, identification of the appropriate level for tests and full reporting of outcomes
☒	☐	Estimates of effect sizes (e.g. Cohen's *d*, Pearson's *r*), indicating how they were calculated

Our web collection on statistics for biologists contains articles on many of the points above.

Software and code

Policy information about availability of computer code

Data collection

- NMR data were acquired on a Bruker DPX-500 (500 MHz) and Bruker DPX-400 (400 MHz) spectrometers running Topspin X version 1.3.10
- GCMS data were acquired using GCsolution version 2.50 SU3 (Shimadzu)
- GC-FID data were acquired using GCsolution version 231.00 (Shimadzu)
- UV-VIS absorption data were acquired using UVProbe version 2.10 (Shimadzu)
- Stopped flow UV-VIS absorption data were acquired using an Applied Photophysics SX20 stopped-flow UV/vis/fluorescence spectrometer equipped with a double mixing system with absorbance and fluorescence detection, double channel, direct coupled photodiode array (185-725 nm), and anaerobic capabilities.
- Fe-57 Mossbauer data were acquired on a See Co. MS4 Mössbauer spectrometer integrated with a Janis SVT-400T He/N2 cryostat.
- Density Functional Theory (DFT) calculations were carried out using Gaussian16, Revision C.01 software. AIM2000 program was used to evaluate the charge density at the iron nucleus for the isomer shift calculation.
- Protein crystallography was acquired remotely at Stanford Synchrotron Radiation Lightsource (SSRL) and the Advanced Photon Source (APS).
- Detailed information regarding all data collection are provided in the Supporting Information.

Data analysis

- NMR spectra were analyzed using MNova version 14.2.1-27684
- GC-MS data were analyzed using GCsolution version 2.50 SU3
- GC-FID data were analyzed using GCsolution version 231.00 (Shimadzu)
- UV-VIS absorption data were analyzed using UVProbe version 2.10 (Shimadzu)
- Stopped flow UV/vis data were analyzed using Applied Photophysics SX20 Data Analysis software and Pro-Kineticist version IV software (Pro K IV) 2012.
- Fe-57 Mossbauer data were analyzed using WMoss (See Co.)
- Protein crystallographic data were analyzed using XDS, CCP4 programs AIMLESS and POINTLESS via an autoXDS script and CrysAlisPro (version 171.41).
- Model building and refinement was done using Phenix (1.19.2), and Coot (0.8.9) and PYMOL 2.5.

• Detailed information regarding all data collection is provided in the Supporting Information.

For manuscripts utilizing custom algorithms or software that are central to the research but not yet described in published literature, software must be made available to editors and reviewers. We strongly encourage code deposition in a community repository (e.g. GitHub). See the Nature Research guidelines for submitting code & software for further information.

Data

Policy information about availability of data

All manuscripts must include a data availability statement. This statement should provide the following information, where applicable:
- Accession codes, unique identifiers, or web links for publicly available datasets
- A list of figures that have associated raw data
- A description of any restrictions on data availability

Data supporting the findings of this study are included in this published article and the supplementary information files. Additional datasets generated during and/or analysed during the current study are available from the corresponding author on reasonable request. Protein crystal structures reported in this manuscript have been deposited in the Protein Data Bank (PDB) under accession codes 8ESS (http://doi.org/10.2210/pdb8ESS/pdb) and 8ESU (http://doi.org/10.2210/pdb8ESU/pdb).

Field-specific reporting

Please select the one below that is the best fit for your research. If you are not sure, read the appropriate sections before making your selection.

☒ Life sciences ☐ Behavioural & social sciences ☐ Ecological, evolutionary & environmental sciences

For a reference copy of the document with all sections, see nature.com/documents/nr-reporting-summary-flat.pdf

Life sciences study design

All studies must disclose on these points even when the disclosure is negative.

Sample size	Enzymatic reactions were repeated in triplicate from independent experiments with different batches of enzyme. Assay yields were typically within 10% error among each run. Stopped-flow and Fe-57 Mossbauer experiments were performed at least in duplicate and using different batches of enzyme and diazo reagent. All presented data are representative results for at least two experiments that were performed independently on different days.
Data exclusions	No data collected in this data were excluded.
Replication	All samples were measured as technical replicates ($n \geq 3$) and are representative results for at least two experiments that were performed independently on different days and that successfully replicated the presented results.
Randomization	Randomization was not applicable to this study. All reagents and catalysts were selected and the reaction conditions were carefully designed.
Blinding	Blinding was not applicable to this study. All experimental data were acquired using automated equipment and analyzed using computational software, eliminating human error.

Reporting for specific materials, systems and methods

We require information from authors about some types of materials, experimental systems and methods used in many studies. Here, indicate whether each material, system or method listed is relevant to your study. If you are not sure if a list item applies to your research, read the appropriate section before selecting a response.

Materials & experimental systems

n/a	Involved in the study	
☒	☐	Antibodies
☒	☐	Eukaryotic cell lines
☒	☐	Palaeontology and archaeology
☒	☐	Animals and other organisms
☒	☐	Human research participants
☒	☐	Clinical data
☒	☐	Dual use research of concern

Methods

n/a	Involved in the study	
☒	☐	ChIP-seq
☒	☐	Flow cytometry
☒	☐	MRI-based neuroimaging
